# Using electronic health records to predict costs and outcomes in stable coronary artery disease

**DOI:** 10.1136/heartjnl-2015-308850

**Published:** 2016-02-10

**Authors:** Miqdad Asaria, Simon Walker, Stephen Palmer, Chris P Gale, Anoop D Shah, Keith R Abrams, Michael Crowther, Andrea Manca, Adam Timmis, Harry Hemingway, Mark Sculpher

**Affiliations:** 1 Centre for Health Economics, University of York, York, UK; 2 Faculty of Medicine and Health, Leeds Institute of Cardiovascular and Metabolic Medicine, University of Leeds, Leeds, UK; 3 Farr Institute of Health Informatics Research, UCL Institute of Health Informatics, University College, London, UK; 4 Department of Health Sciences, University of Leicester, Leicester, UK; 5 NIHR Biomedical Research Unit, Barts and the London NHS Trust, London, UK

## Abstract

**Objectives:**

To use electronic health records (EHR) to predict lifetime costs and health outcomes of patients with stable coronary artery disease (stable-CAD) stratified by their risk of future cardiovascular events, and to evaluate the cost-effectiveness of treatments targeted at these populations.

**Methods:**

The analysis was based on 94 966 patients with stable-CAD in England between 2001 and 2010, identified in four prospectively collected, linked EHR sources. Markov modelling was used to estimate lifetime costs and quality-adjusted life years (QALYs) stratified by baseline cardiovascular risk.

**Results:**

For the lowest risk tenth of patients with stable-CAD, predicted discounted remaining lifetime healthcare costs and QALYs were £62 210 (95% CI £33 724 to £90 043) and 12.0 (95% CI 11.5 to 12.5) years, respectively. For the highest risk tenth of the population, the equivalent costs and QALYs were £35 549 (95% CI £31 679 to £39 615) and 2.9 (95% CI 2.6 to 3.1) years, respectively. A new treatment with a hazard reduction of 20% for myocardial infarction, stroke and cardiovascular disease death and no side-effects would be cost-effective if priced below £72 per year for the lowest risk patients and £646 per year for the highest risk patients.

**Conclusions:**

Existing EHRs may be used to estimate lifetime healthcare costs and outcomes of patients with stable-CAD. The stable-CAD model developed in this study lends itself to informing decisions about commissioning, pricing and reimbursement. At current prices, to be cost-effective some established as well as future stable-CAD treatments may require stratification by patient risk.

## Introduction

Cardiovascular disease (CVD) is a leading cause of mortality in England with approximately a third of all deaths attributed to it.^1^ The combination of an ageing population and improvements in survival after acute coronary syndrome^2^ has resulted in a large and growing number of patients with stable coronary artery disease (stable-CAD). CVD has, therefore, also become a major source of morbidity and healthcare resource use: there are >5 million people living with CVD in England costing the National Health Service (NHS) more than £30 billion per year.^3^ ^4^ The stable-CAD population serves as an important example of a patient population suffering from a long-term condition. With such conditions becoming increasingly prevalent, questions regarding their prognosis have become increasingly important.^5^ ^6^ The prognosis for patients with stable-CAD is particularly topical with new treatments,^7^ and new applications of existing treatments,^8^ currently undergoing phase III trials in this patient population.

Thus far, the majority of models to estimate the costs and health effects of CVD have focused on primary prevention,^9^ ^10^ have made predictions only over relatively short time horizons (up to 10 years)^11^ so are unable to estimate lifetime costs and health effects, are based on selected samples^12^ potentially biasing baseline risk and cost estimates hence limiting their generalisability or fail to model all relevant endpoints and their interdependence.^13^ The use of linked electronic health records (EHR) can help to address many of these limitations in modelling the costs and outcomes in chronic diseases providing a source of long-term data, capturing a wide range of clinical endpoints and recording resource use in a real-world setting. As far as we are aware, there has been limited use of EHR in decision modelling.

The availability of primary care data linked with hospitalisation data, disease-specific registries and mortality data makes the English NHS an attractive setting in which to develop and demonstrate our approach for modelling the long-term costs and outcomes of chronic disease. The CALIBER (CArdiovascular disease research using Linked BEspoke studies and Electronic Health Records) data platform^14^ used in this study combines these key datasets and has been shown to be a valuable resource for cardiovascular epidemiology.^12^ ^15–17^ This paper reports on the use of CALIBER to model prognosis in patients with stable-CAD, estimating their baseline risk of experiencing further CVD events and then predicting both costs and key health outcomes over the lifetime of these patients stratified by their baseline CVD risk. In doing so, the model provides a better understanding of the implications of this growing population under current standards of care as well as a framework for the evaluation of the cost-effectiveness of new treatment strategies, potentially differentiated by risk group.

## Methods

### Patient population

The model was based on the analysis of 94 966 patients with stable-CAD from the CALIBER collaboration. CALIBER links primary care data from the Clinical Practice Research Datalink with EHR from the Myocardial Ischaemia National Audit Project Registry, hospital inpatient records from Hospital Episode Statistics and cause-specific mortality from the Office for National Statistics. The CALIBER dataset has been described in detail by Denaxas *et al.*^14^ Patients with stable-CAD were defined as those patients in the CALIBER dataset who were event free for at least 6 months after having had unstable angina, ST elevation myocardial infarction (STEMI) or non-STEMI (NSTEMI) or those patients with stable angina or other coronary heart disease (CHD) diagnoses. The median follow-up of these patients was 4.2 (IQR 1.9–6.9) years, during which 16 783 patients died and 8203 patients experienced one or more non-fatal coronary outcomes.

### Endpoints

The primary clinical endpoints were first occurrences of non-fatal myocardial infarction (MI), ischaemic stroke and haemorrhagic stroke, as well as CVD and non-CVD mortality. Other clinical endpoints were CVD and non-CVD mortality following a non-fatal event. These were combined to produce the primary economic outputs from the model which were quality-adjusted life years (QALYs) as well as total and CVD-specific costs, each predicted over the remaining lifetime of the patient. The model was also used to produce estimates of event rates and disease progression over time stratified by baseline CVD risk.

### Model

**Figure 1:**
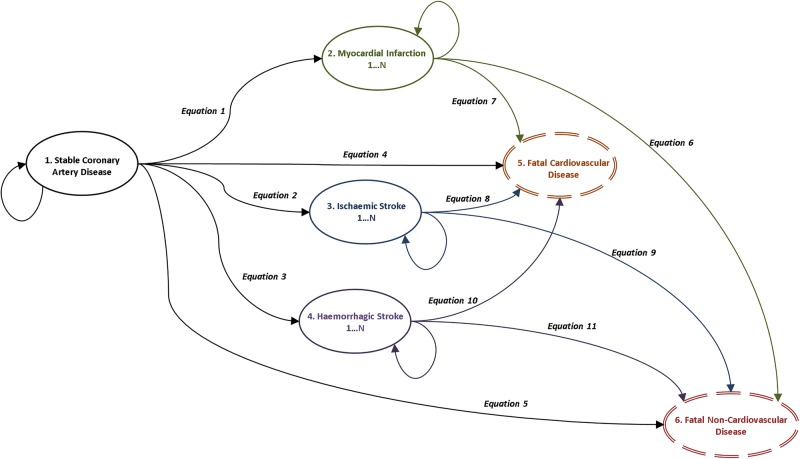
Structure of the Markov model and the role played by the 11 risk equations that we use to model disease progression.

A state transition model (shown in figure 1) was developed to capture the natural history of patients with stable-CAD. The structure of the model was determined with reference to both previous models in CVD^13^ and expert clinical advice. All patients entered the model in the stable-CAD state and progressed through the model until they experienced either CVD or non-CVD mortality. The time horizon of the model was, therefore, the patient’s remaining lifetime. The model captured time varying and age-dependent risks, costs and health-related quality of life (HRQoL) in 90-day segments. Costs and HRQoL were attached to model states and, in order to stratify by patients’ baseline risk, adjusted for patient covariates at baseline as well as for age and for time elapsed following non-fatal events. Model predicted costs, life years and QALYs were discounted at 3.5% per annum in keeping with the guidelines in England.^18^ While only first occurrences of non-fatal CVD events were explicitly modelled, further non-fatal events were implicitly captured in the time varying risk, cost and HRQoL estimates.

### Statistical modelling of risk equations

Rapsomaniki *et al*^19^ developed, tested and validated a range of prognostic models for patients with stable-CAD using the CALIBER dataset. We built on their recommended prognostic model, using it as the basis for the risk equations underpinning the prediction of the five primary clinical endpoints. Using the prognostic factors and missing data imputation algorithm of Rapsomaniki *et al*^19^ we estimated various parametric survival models (generalised gamma, lognormal, Weibull, exponential) for each of the five endpoints. For each endpoint the best fitting parametric model was selected as determined by the Akaike information criteria. Predictions resulting from the selected models were assessed for plausibility by clinical experts (AT, CPG, ADS, HH). Key prognostic factors included in the models were demographic measures (age, sex, social deprivation), stable-CAD subtype (stable angina, unstable angina, STEMI, NSTEMI and other CHD), use of long-acting nitrates, whether coronary artery bypass graft or percutaneous coronary intervention (PCI) had been performed in the 6 months following CAD diagnosis, previous MI, smoking, blood pressure, diagnosis of hypertension, diabetes, lipids, CVD comorbidities (heart failure, peripheral arterial disease, atrial fibrillation, stroke), non-CVD comorbidities (chronic renal disease, chronic obstructive pulmonary disease, cancer, chronic liver disease), psychosocial factors (depression, anxiety) and clinically assessed biomarkers (heart rate, white cell count, haemoglobin, creatinine).

Risk equations for the six subsequent events, namely, CVD and non-CVD mortality following non-fatal MI, ischaemic stroke and haemorrhagic stroke, were estimated in a similar way. However, due to the greatly reduced numbers of events observed, these use only sex and age at time of non-fatal event as covariates. Non-CVD mortality beyond the maximum follow-up in the CALIBER dataset (10 years) was based on age/sex-specific non-CVD mortality from national life tables.^20^

These risk equations were developed into cumulative incidence functions which were then combined using a competing risks framework to account for the interdependence of the outcomes. We used methods outlined by Putter *et al*^21^ that acknowledge state transition probabilities are affected by the event being modelled and also by the other events that could occur from a given health state. Survival models were estimated using R (V.3.1.0) and the R package flexsurv (V.0.3).

### Resource use and costs

Healthcare resource use was estimated directly from the CALIBER dataset. A panel was constructed using a 90-day cycle length for patients with stable-CAD in CALIBER capturing resource use in terms of hospital episodes, use of drugs, diagnostic tests and primary care consultations. Costs were attached to this resource use using the NHS reference costs,^22^ NHS prescription cost analysis^23^ and Personal Social Services Research Unit (PSSRU) unit costs for primary care^24^ datasets. All costs were calculated from a health systems perspective and based on the price year 2011/2012. Panel data models were used to estimate patient costs adjusted for the prognostic factors used in the model, as well as for the key CVD events in the model. This allowed us to attach costs to model states adjusted for baseline patient characteristics and event history.

### Health-related quality of life

HRQoL estimates were not available from the CALIBER dataset. Instead a catalogue of EQ-5D scores for the UK^25^ was used to calculate age-specific, condition-specific and event-specific HRQoL. These were attached to states in the model to calculate patient-specific estimates of remaining lifetime QALYs.

### Analysis

Given that the model was designed to be used with a heterogeneous population, results were produced stratified by risk group. The 5 year baseline risk of experiencing at least one CVD event for each patients with stable-CAD in the CALIBER dataset was predicted based on the estimated risk equations given the patient's baseline covariate values as input parameters. The baseline values were those from the prognostic factors used in the risk equations measured at the point that the patient entered into the stable-CAD cohort. Patients were ranked by risk predictions and grouped into 10 equally sized risk groups. Model results were calculated at the mean baseline covariate value across patients within each risk group. In addition, estimates were predicted for a representative patient within each of the 10 risk groups demonstrating both the population-level and patient-level results produced by the model. The model was evaluated probabilistically by means of a Monte Carlo simulation run for 1000 iterations in order to incorporate and characterise the uncertainty in the model inputs.^26^

The model was used to calculate life expectancy, QALYs, total healthcare costs and CVD-specific healthcare costs for standard care, as well as for indicative new treatments assumed to reduce CVD risks by 10%, 20%, 30% and 40%. The indicative treatments were assumed to have constant costs and treatment effects, no direct effect on the risk of non-CVD mortality and no side-effects. When interpreting the results of this analysis it should be recognised that these assumptions may not hold in practice. The results were used to estimate the maximum price that could be charged for the new treatments in each of the risk groups assuming a range of cost-effectiveness thresholds between £10 000 and £40 000 per QALY. National Institute for Health and Care Excellence (NICE) employ a threshold ranging between £20 000 and £30 000 per QALY^18^ for considering an intervention cost-effective in England, and recent empirical evidence provides a central estimate of the threshold in England of approximately £13 000 per QALY.^27^

Further details about the (a) patients with stable-CAD in the CALIBER dataset, (b) the economic model, (c) the estimation of costs and transition probabilities for use in the model, (d) the risk equations used to estimate model transition probabilities, (e) patient profiles for the 10 representative patients and (f) extended tables of results can be found in the accompanying online supplementary material appendices. The full model source code detailing all calculations performed in the model, including the model input parameters for the 10 risk groups and 10 representative patients as well as detailed instructions on how to run the model, are available from: https://github.com/miqdadasaria/caliber-scad-model.

## Results

The average baseline patient covariates by risk group are shown in table 1. For the cohort, the mean age at cohort entry was 67 years for males and 72 years for females. Stable angina (47%) was the most frequent stable-CAD subgroup and STEMI (7%) the least. One in 10 patients had received PCI within the previous 6 months, over a quarter had heart failure, nearly one in five had depression at the time of stable-CAD diagnosis and one in six had atrial fibrillation.

**Table 1:** Patient characteristics by risk group

Risk group	Lowest risk	2	3	4	5	6	7	8	9	Highest risk	Overall
Patient average covariate profiles based on tenths of patient population grouped by 5-year risk of composite CVD event estimated at baseline											
Number of patients in dataset	10 035	9903	9797	9626	9516	9455	9382	9335	9249	8668	94 966
5-year risk (%; average across patients)	3.69	5.70	7.37	9.15	11.20	13.71	17.14	22.14	30.42	52.37	16.68
5-year risk (%; at average covariate values)	3.46	5.43	6.95	8.53	10.36	12.57	15.64	20.07	27.23	44.18	11.64
Sociodemographic characteristics											
Sex (% female)	64	48	42	39	37	37	38	42	44	46	44
Age (years if male)	49	55	59	62	65	67	71	74	77	81	67
Age (years if female)	53	62	67	70	73	75	78	80	83	87	72
Age (weighted average)	52	59	62	65	68	70	73	76	80	84	69
Most deprived quintile (%)	15	17	18	19	20	21	21	22	22	24	20
Stable-CAD diagnosis (%)											
NSTEMI	0	1	3	5	8	10	12	17	23	43	10
STEMI	1	4	8	12	13	14	13	9	6	4	7
Unstable angina	10	13	12	12	12	12	13	15	17	15	14
Stable angina	78	65	56	49	43	39	37	34	29	18	47
Non-specific CHD	11	17	20	22	24	24	25	26	25	20	23
Stable-CAD severity (%)											
PCI in past 6 months	9	12	13	14	13	13	11	9	6	4	9
CABG in past 6 months	9	7	6	5	5	4	4	3	2	1	4
Previous/recurrent MI	2	6	10	14	18	23	26	29	32	43	18
Use of nitrates	10	16	19	21	24	28	33	37	43	56	28
CVD risk factors											
Smoking status (%)											
Current smoker	31	35	36	37	38	38	37	35	32	30	35
Ex-smoker	27	30	31	32	32	33	34	34	34	34	32
Never smoked	41	35	33	31	30	29	29	31	33	36	33
Hypertension (%)	69	70	71	71	72	74	76	79	83	87	76
Diabetes (%)	4	8	10	12	14	16	18	21	24	32	16
Total cholesterol (mmol/L)	4.95	4.91	4.84	4.79	4.74	4.74	4.70	4.68	4.64	4.54	4.79
HDL (mmol/L)	1.41	1.37	1.35	1.35	1.35	1.35	1.36	1.37	1.37	1.35	1.37
CVD comorbidities (%)											
Heart failure	5	7	9	12	15	19	27	37	52	73	26
Peripheral arterial disease	1	2	3	4	6	8	10	13	16	25	8
Atrial fibrillation	3	5	7	9	10	13	16	21	29	43	15
Stroke	0	1	1	2	3	5	8	14	22	39	9
Non-CVD comorbidities (%)											
Chronic kidney disease	2	2	3	4	4	5	7	9	12	20	7
Chronic obstructive pulmonary disease	20	20	20	21	22	23	25	27	28	30	23
Cancer	4	5	6	7	8	9	11	13	14	12	9
Chronic liver disease	0	1	1	1	1	1	1	1	1	1	1
Psychosocial characteristics											
Depression at diagnosis (%)	20	17	15	15	14	14	15	17	18	21	17
Anxiety at diagnosis (%)	7	6	6	7	7	7	8	8	10	12	8
Biomarkers											
Heart rate (bpm)	72	71	71	71	71	71	72	73	74	76	72
Creatinine (mmol/L)	88	92	95	96	98	100	101	104	109	125	100
White cell count (10^9^/L)	6.81	7.05	7.19	7.31	7.44	7.54	7.62	7.76	7.88	8.22	7.46
Haemoglobin (g/100 mL)	1.43	1.43	1.42	1.41	1.39	1.37	1.35	1.32	1.28	1.22	1.36

Deprivation measured by index of multiple deprivation, 2010. All values in table are means. Percentage of missing data imputed: smoking status 32%, total cholesterol 54%, HDL 55%, heart rate 78%, creatinine 38%, white cell count 56% and haemoglobin 53%.

CABG, coronary artery bypass graft; CAD, coronary artery disease; CHD, coronary heart disease; CVD, cardiovascular disease; HDL, high-density lipoprotein; MI, myocardial infarction; NSTEMI, non-ST segment elevation myocardial infarction; PCI, percutaneous coronary intervention; stable-CAD, stable coronary artery disease; STEMI, ST segment elevation myocardial infarction.

There was large variation in CVD risk between the lowest and highest risk groups, with an absolute difference in 5-year risk between the lowest and highest risk group of 40.7%. The risk of clinical events positively correlated with age, higher levels of CVD risk factors (such as hypertension and diabetes) and higher prevalence of CVD comorbidities. There were no obvious trends in the key modifiable CVD risk factors such as the lipid profile.

**Figure 2:**
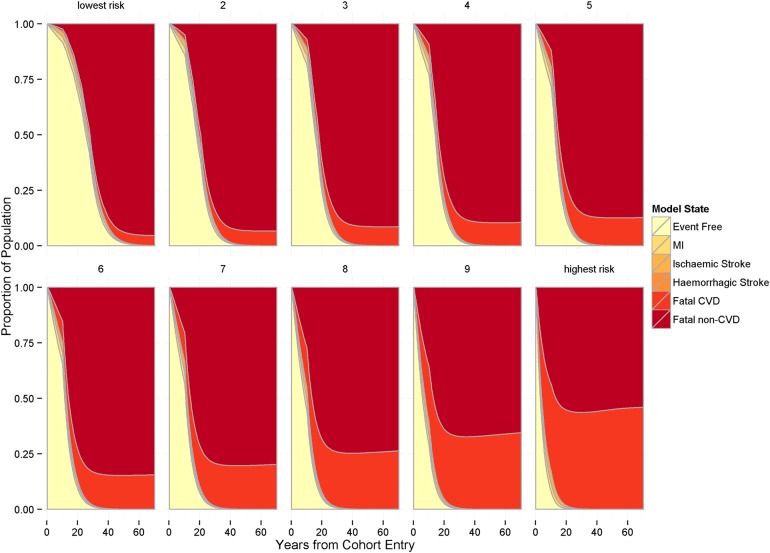
Proportion of patients in each of the six model states over time as predicted by the Markov model used in this study. Each plot within the panel represents a risk decile as categorised by the baseline 5-year CVD event risk ranging from the lowest risk decile (1) to the highest risk decile (10). As can be seen in the plots the model is run until all the patients in the cohort have experienced either a fatal CVD or a fatal non-CVD event. CVD, cardiovascular disease; MI, myocardial infarction.

The modelled progression of CVD over time by risk group is shown in figure 2. Higher risk groups were predicted to have much higher levels of CVD mortality compared with lower risk groups, whereas the latter were predicted to remain event free for a much longer period and were more likely to die of non-CVD-related causes.

Summary model results by risk group are shown in table 2. The risk of all non-fatal events increased with overall CVD risk, and the risk of non-CVD mortality declined with overall CVD risk. Lower risk patients were estimated to have greater remaining life expectancy, QALYs and healthcare costs. For low risk patients (5-year CVD risk 3.5%), the remaining expected discounted lifetime healthcare costs were £62 210, and patients had 12.0 expected discounted QALYs remaining. For the highest risk group (5-year CVD risk 44.2%), the remaining expected discounted lifetime healthcare costs were £35 549, and patients had 2.8 remaining expected discounted QALYs.

**Table 2:** Model results by risk group

Risk group (95% CI)	Lowest risk	2	3	4	5	6	7	8	9	Highest risk
Model results split by 5-year risk of composite CVD event										
Life years	26.81 (26.63 to 26.98)	19.62 (19.48 to 19.80)	17.34 (17.18 to 17.53)	15.63 (15.47 to 15.84)	14.26 (14.08 to 14.49)	13.03 (12.83 to 13.28)	11.92 (11.69 to 12.21)	10.48 (10.21 to 10.84)	8.52 (8.19 to 8.94)	5.51 (5.09 to 6.02)
Discounted life years*	16.77 (16.69 to 16.85)	13.66 (13.58 to 13.75)	12.5 (12.41 to 12.61)	11.56 (11.46 to 11.68)	10.76 (10.65 to 10.89)	9.99 (9.87 to 10.15)	9.26 (9.11 to 9.44)	8.27 (8.10 to 8.50)	6.90 (6.67 to 7.17)	4.67 (4.38 to 5.01)
QALYs	19.11 (18.06 to 19.93)	13.97 (13.26 to 14.54)	12.29 (11.66 to 12.80)	11.01 (10.45 to 11.48)	9.97 (9.44 to 10.41)	9.03 (8.53 to 9.45)	8.13 (7.65 to 8.53)	6.99 (6.54 to 7.40)	5.50 (5.09 to 5.89)	3.34 (3.01 to 3.72)
Discounted QALYs*	12.04 (11.45 to 12.53)	9.77 (9.31 to 10.17)	8.9 (8.47 to 9.25)	8.18 (7.78 to 8.51)	7.55 (7.17 to 7.87)	6.95 (6.58 to 7.25)	6.34 (5.98 to 6.63)	5.55 (5.21 to 5.84)	4.47 (4.16 to 4.76)	2.85 (2.60 to 3.13)
Total costs (£,1000s)	117 (65 to 168)	81 (55 to 108)	73 (54 to 92)	68 (54 to 83)	65 (53 to 76)	62 (54 to 71)	61 (55 to 69)	59 (54 to 65)	54 (49 to 60)	43 (38 to 49)
Discounted total costs (£,1000s)*	62 (34 to 90)	51 (34 to 67)	48 (36 to 60)	47 (37 to 56)	45 (38 to 53)	45 (39 to 51)	45 (41 to 50)	45 (41 to 49)	42 (39 to 46)	36 (32 to 40)
CVD costs (£,1000s)	72 (29 to 114)	52 (30 to 74)	48 (31 to 64)	45 (33 to 58)	43 (34 to 53)	42 (35 to 50)	42 (36 to 48)	41 (37 to 46)	38 (34 to 43)	31 (27 to 35)
Discounted CVD costs (£,1000s)*	38 (15 to 60)	32 (19 to 46)	31 (21 to 41)	31 (23 to 39)	31 (24 to 37)	31 (26 to 36)	31 (27 to 35)	31 (28 to 34)	30 (27 to 33)	26 (23 to 29)
Time to first event (years)	24.55 (24.31 to 24.76)	17.80 (17.64 to 17.95)	15.62 (15.47 to 15.75)	13.98 (13.85 to 14.11)	12.67 (12.54 to 12.8)	11.49 (11.36 to 11.62)	10.43 (10.29 to 10.57)	9.00 (8.85 to 9.15)	7.06 (6.91 to 7.22)	4.07 (3.90 to 4.23)
MI as primary endpoint (%)	6.00 (5.55 to 6.49)	7.11 (6.73 to 7.49)	8.06 (7.72 to 8.43)	8.94 (8.61 to 9.29)	9.84 (9.50 to 10.15)	10.70 (10.39 to 11.01)	11.59 (11.28 to 11.90)	12.33 (12.01 to 12.64)	12.89 (12.57 to 13.22)	14.3 (13.87 to 14.73)
Ischaemic stroke as primary endpoint (%)	5.51 (5.01 to 6.06)	5.70 (5.34 to 6.11)	6.06 (5.73 to 6.43)	6.39 (6.07 to 6.74)	6.80 (6.48 to 7.11)	7.37 (7.05 to 7.68)	8.29 (7.95 to 8.63)	9.31 (8.96 to 9.68)	10.07 (9.72 to 10.43)	9.97 (9.58 to 10.38)
Haemorrhagic stroke as primary endpoint (%)	0.67 (0.48 to 0.89)	0.67 (0.54 to 0.81)	0.71 (0.59 to 0.82)	0.72 (0.62 to 0.84)	0.74 (0.65 to 0.84)	0.76 (0.67 to 0.86)	0.79 (0.70 to 0.89)	0.78 (0.69 to 0.88)	0.7 (0.61 to 0.81)	0.48 (0.40 to 0.57)
CVD mortality (%)	4.48 (3.45 to 5.55)	6.60 (5.45 to 7.51)	8.52 (7.22 to 9.47)	10.39 (8.97 to 11.44)	12.63 (11.07 to 13.85)	15.48 (13.78 to 17.07)	20.17 (18.17 to 22.63)	26.29 (23.61 to 30.18)	34.46 (30.65 to 39.32)	45.95 (41.34 to 50.07)
Non-CVD mortality (%)	95.46 (94.40 to 96.49)	93.40 (92.49 to 94.55)	91.48 (90.53 to 92.78)	89.60 (88.56 to 91.03)	87.37 (86.15 to 88.93)	84.52 (82.93 to 86.22)	79.83 (77.37 to 81.83)	73.71 (69.82 to 76.39)	65.54 (60.68 to 69.35)	54.05 (49.93 to 58.66)

*Discounted figures presented in this table are discounted at 3.5% per annum.

CVD, cardiovascular disease; MI, myocardial infarction; QALYs, quality-adjusted life years.

**Figure 3:**
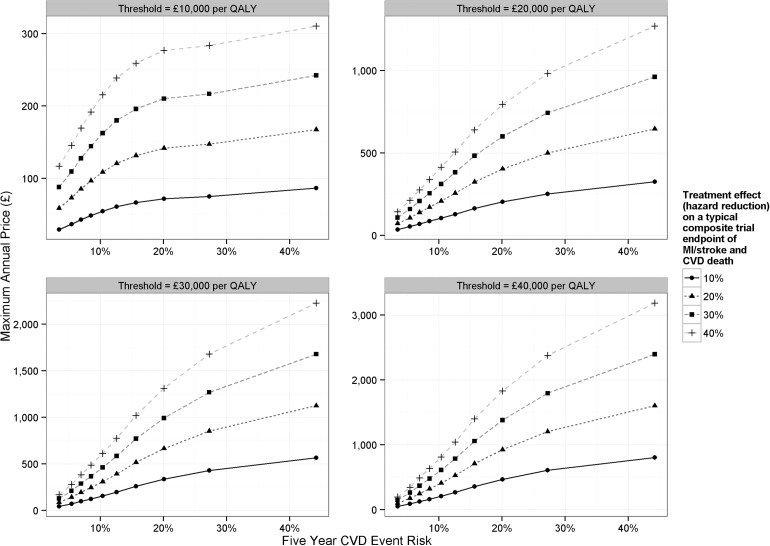
Maximum annual price for therapies as a function of baseline 5-year CVD event risk. Each plot within the panel shows the results at a given cost-effectiveness threshold ranging from £10 000 to £40 000 per QALY. The lines within the plots represent the different efficacies of our modelled treatments having hazard reductions on CVD endpoints associated with them ranging from 10% to 40%. CVD, cardiovascular disease; MI, myocardial infarction; QALYs, quality-adjusted life years.

Figure 3 shows the maximum price that the health system should be willing to pay for new treatments targeted at each risk group that reduce CVD hazards by between 10% and 40%. This maximum price increased with both increasing baseline risk and with larger treatment effects in terms of proportionate risk reduction.

More detailed breakdowns of these results as well as results presented for the representative patients drawn from each risk group can be found in online supplementary appendix (f).

## Discussion

We report the first comprehensive lifetime model of stable-CAD based on long-term EHR data. The model encompasses a full range of CVD endpoints and accounts for the interdependence of CVD risks among patients with stable-CAD. The sample sizes, duration of follow-up and the large number of endpoints and risk factors captured by the multisource EHR dataset (CALIBER) provided the opportunity to build a model which more fully and accurately captured the biological and medical nuances of such a condition. In quantifying the expected costs, life expectancy and quality-adjusted life expectancy of patients with stable-CAD, this analysis provides a means to plan budgets and services for such patients in the NHS in particular, and in health systems in developed countries more generally.

We found that at NICE's lower bound cost-effectiveness threshold (£20 000 per QALY), a treatment aimed at the lowest risk patients (5-year risk of 3.5%), would be cost-effective with annual prices up to £36, £72, £108 or £143 if the treatment was able to reduce CVD risk by 10%, 20%, 30% and 40%, respectively. For the highest risk patients (5-year risk of 44.2%), the respective maximum prices would be £325, £645, £961 or £1269. For comparison, statins commonly used by these patients reduce CVD risk by approximately a third^28^ and cost £16 per patient per year,^29^ whereas the annual cost of new antiplatelet agents can be up to £712 per patient per year.^29^ These estimates provide a basis for developers of new medications and health technologies for stable-CAD to define necessary effect sizes that they will need to demonstrate to be considered value for money by health systems.

In this study it has been shown that using EHR data, in combination with an analytical model such as that used by NICE in the English NHS, provides a powerful framework within which to assess the cost-effectiveness of new technologies. In the many healthcare systems with constrained budgets, cost-effectiveness analysis provides a means of comparing the additional health benefits from a new intervention with the health other patients forgo because expenditure on other types of treatments is necessarily curtailed in order to finance the new intervention (opportunity costs).^30^ The current analysis uses this approach as a basis for identifying the minimum treatment effect a new intervention for stable-CAD will have to achieve at a given price (or the maximum price for a given treatment effect) and cost-effectiveness threshold. These necessary treatment effects and prices will inevitably vary according to patients’ underlying risk of CVD events.

There are very few comparable studies that focus on modelling the costs and health effects over the lifetime of patients with stable-CAD. Studies that we are aware of in this area^13^ are typically based on short-term trial data, model only a subset of the relevant CVD endpoints and make predictions over short time horizons. Models suitable for the economic evaluation of health technologies in disease areas such as CVD where there are substantial mortality impacts need to estimate all relevant healthcare costs and health outcomes over the remaining lifetimes of patients. This is why in our study, despite having 10 years of follow-up data, we still required a model to extrapolate up to a maximum of 60 years beyond our data to estimate total lifetime costs and consequences for the full cohort of modelled patients. Limitations of our study are that HRQoL data were not recorded in the CALIBER dataset and so had to be drawn from external studies; that changes in prognostic risk factors over time were not explicitly modelled; instead the equations underpinning our model were informed by the baseline values of these risk factors; the dataset we used did not contain left ventricular ejection fraction which is an important prognostic factor in this patient population; and that the long follow-up period of our dataset may mean that the modelled risk equations may not fully reflect contemporary risk levels in the population. Additionally a number of structural assumptions had to be made for modelling purposes and these are detailed in online supplementary appendix (b).

The model we have produced allows policy makers to quantify and understand both the health and the cost burden of stable-CAD and serves as a basis for evaluating the cost-effectiveness of new treatments targeted at reducing CVD risk in this population. Our results suggest that, for the vast majority of patients with stable-CAD, it is likely that low cost interventions to improve adherence to existing secondary prevention drugs should be prioritised over high cost new treatments. It is also notable from our results that, even among the groups with the highest CVD risk, more patients are predicted to die of non-CVD-related causes than of CVD-related causes. This highlights the vital role of primary care in the holistic management of both CVD and non-CVD risk for these patients.

**Key messages**

**What is already known on this subject?**

- Electronic health records have been shown to be useful in prognosis, but thus far their use in decision analytic models and cost-effectiveness analysis has been limited.
- The recent improvement in acute coronary syndrome survivorship means that a growing number of people are living with cardiovascular disease.

**What might this study add?**

- This study provides the first lifetime model of the costs and health effects of patients with stable coronary artery disease based on long-term linked electronic health records, predicting key cardiovascular endpoints for these patients and capturing the interdependence of these endpoints.

**How might this impact on clinical practice?**

- This model can be used to evaluate and to target appropriately new treatments as they emerge for this patient population as well as to inform commissioning, pricing and reimbursement decisions.

## Supplementary Material

Web appendix A

Web appendix B

Web appendix C

Web appendix D

Web appendix E

Web appendix F
