# Supplementary material for: Using electronic health records to predict costs and outcomes in stable coronary artery disease
Source: Heart. 2016 Feb 10;102(10):755–62. doi: 10.1136/heartjnl-2015-308850 (PMC4849559; doi:10.1136/heartjnl-2015-308850)
Supplement: Web appendix A [file heartjnl-2015-308850-s1.pdf]

Modelling lifetime costs and health outcomes for patients with stable coronary artery disease

Appendix A: CALIBER dataset

Patient population consists of patients with stable coronary artery disease (SCAD) in our linked dataset who have had no event in the 180 days post SCAD diagnosis. This is a total of 94,966 patients observed between January 2001 and March 2010. This comprises 12,839 patients with unstable angina as their index event, 6,276 patients with STEMI as their index event, 9,304 patients with NSTEMI as their index event, 45,038 patients with stable angina and 21,509 patients with other CHD diagnoses. Median follow up for these patients was 4.2 (IQR 1.9 to 6.9) years though patients were censored from the dataset throughout the 10 year follow up period as described in the figure.

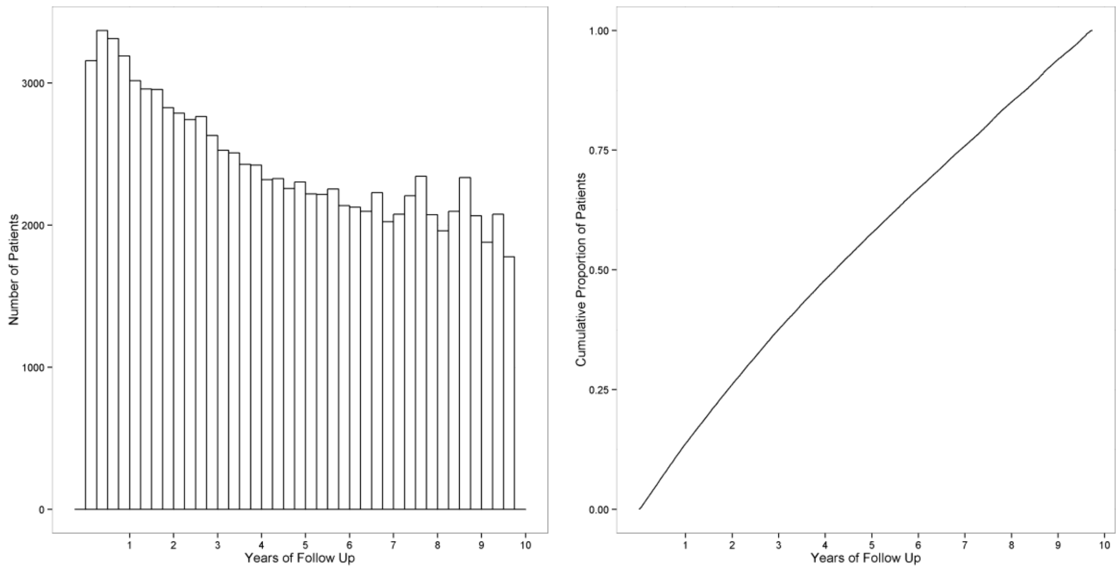

Reasons for leaving the dataset were either death (16,783 of which 6,800 were from cardiovascular causes) or administrative censoring both during (16,790) and at the end of (61,393) the period of observation. Administrative censoring during the period of observation was due to patients moving away from a primary care practice that contribute data to the CPRD dataset.

Not all patients entered the cohort at the start of the study in 2001, rather patients entered the cohort once they had experienced a qualifying event during the study period. We counted events and the time from cohort entry to experience each event at the patient level. Where multiple records for a death were recorded in the datasets constituting CALIBER the earliest date attributed to the death was attributed as the patient’s date of death. For other events repeated recordings of the same event within a 30 day window were considered to be records for the same event and the earliest recorded event date was attributed to that event for the patient.

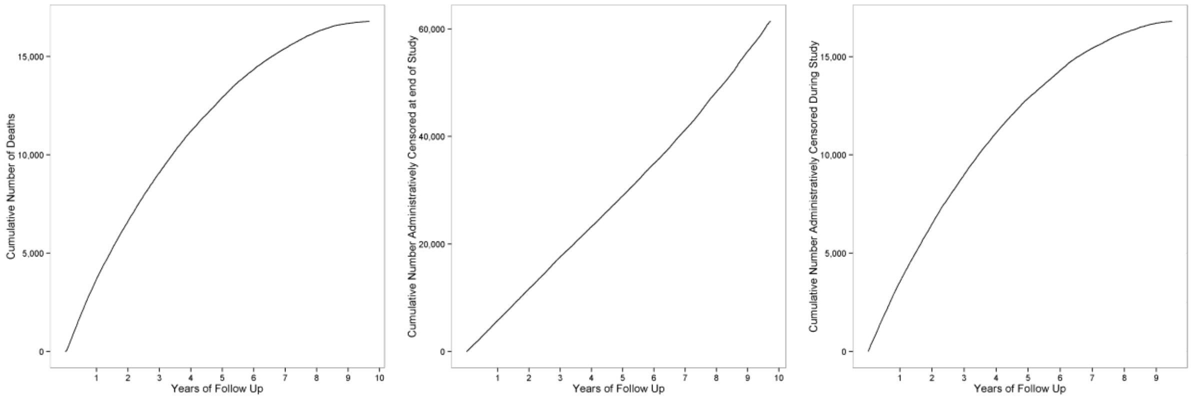

We looked at primary endpoints of type myocardial infarction (4,719), ischaemic stroke (3,222), haemorrhagic stroke (262), death from cardiovascular causes (5,536) and death from non-cardiovascular causes (8,663) as first events experienced subsequent to cohort entry. The validation study by Herrett E, Shah A. D, Boggon R, et al. (Completeness and diagnostic validity of recording acute myocardial infarction events in primary care, hospital care, disease registry, and national mortality records: cohort study. BMJ 2013 http://www.ncbi.nlm.nih.gov/pubmed/23692896) demonstrates the importance of using information from the multiple sources across the linked EHR datasets to determine the occurrence of events, we follow this recommendation with our events being defined using the CPRD, HES, ONS and MINAP codes described on the CALIBER data portal: https://www.caliberresearch.org/portal.

The distributions of times to primary endpoints measured in the time from entry into the cohort are shown in the figures below.

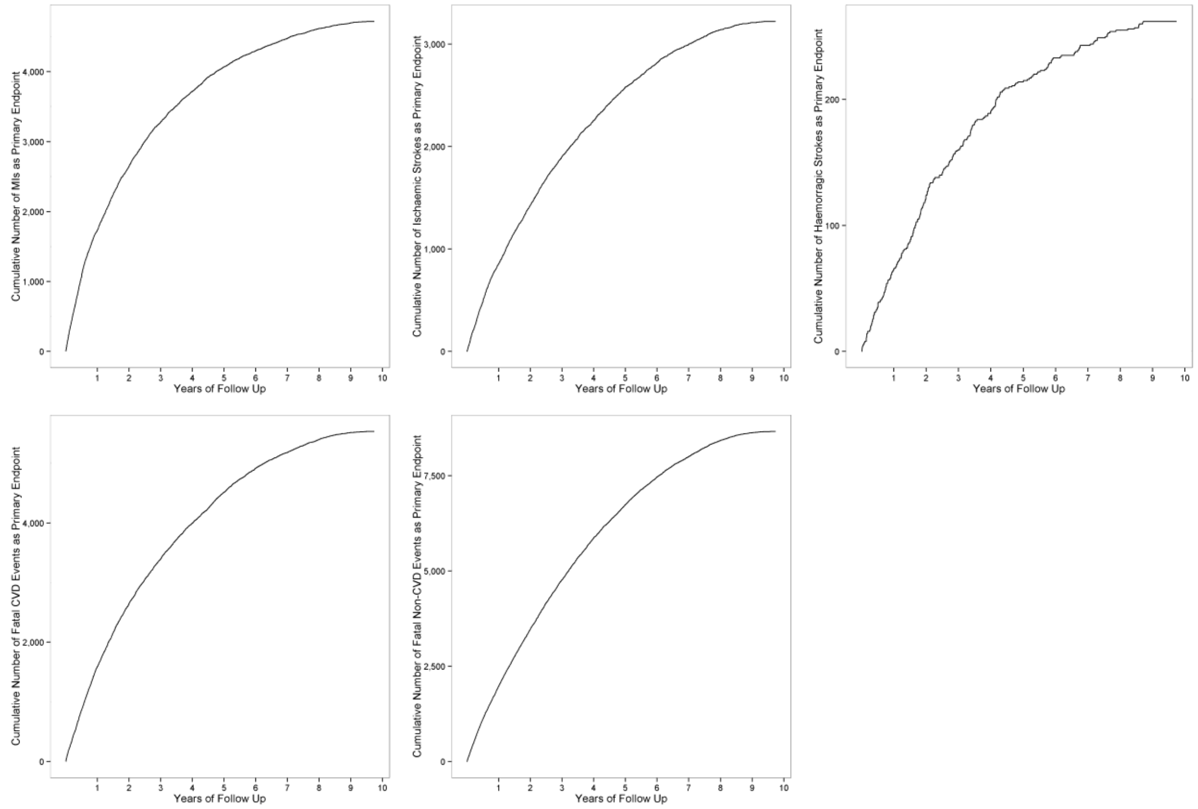

We also looked at deaths from CVD and non-CVD causes following a non-fatal primary endpoint. After an MI we observe 813 CVD deaths and 760 non-CVD deaths, after ischaemic stroke we observe 410 CVD deaths and 525 non-CVD deaths and after haemorrhagic stroke we observe 41 CVD deaths and 35 non-CVD deaths in the CALIBER dataset.

Multiple imputation was used to handle missing covariate values in the CALIBER dataset that was used in estimating the models. Full details about the imputation model used can be found in this technical appendix:
http://eurheartj.oxfordjournals.org/content/ehj/suppl/2013/12/01/eht533.DC1/eht533supp1.pdf

Our study protocol was submitted to CPRD and approved by the Independent Scientific Advisory Committee (ISAC) on the 4th December 2012. Protocol title “Cost effectiveness analyses of treatments for patients with chronic stable angina”, protocol number 12_132R.
