# Supplementary material for: Using electronic health records to predict costs and outcomes in stable coronary artery disease
Source: Heart. 2016 Feb 10;102(10):755–62. doi: 10.1136/heartjnl-2015-308850 (PMC4849559; doi:10.1136/heartjnl-2015-308850)
Supplement: Web appendix B [file heartjnl-2015-308850-s2.pdf]

Modelling lifetime costs and health outcomes for patients with stable coronary artery disease

Appendix B: CALIBER economic model

A Markov state transition model was constructed to model the pathway of stable coronary artery disease (SCAD) patients. The model captured the primary endpoints of first MI, ischaemic stroke, haemorragic stroke. fatal CVD event and non-fatal CVD events after the cohort entry date as well as any subsequent CVD or non-CVD mortality. All patients start the model in the SCAD state and progress through the model until they die of either CVD related or non-CVD related causes. While only first occurrences of non-fatal CVD events are explicitly modelled, further non-fatal events are implicitly captured in the time varying risk, cost and HRQoL estimates used in the model.

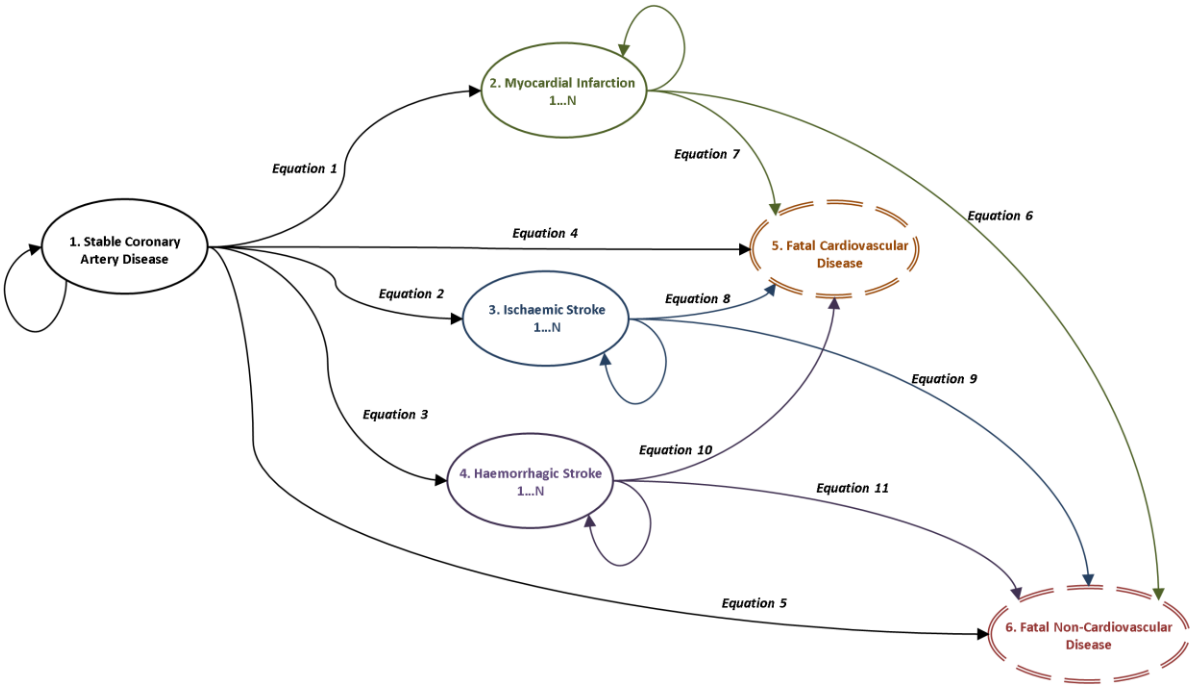

The eleven risk equations corresponding to the model transitions were estimated using flexible parametric survival models. The detail of these estimated risk equations is provided in supplementary appendix (d). These risk equations were combined in a competing risks framework to account for the interdependence of the modelled events following the methods outlined in Putter et al (2007) [Tutorial in biostatistics: Competing risks and multi-state model in Statistics in Medicine 26:2389-2430]. This was used to estimate cumulative incidences of the transitions modelled which in turn was used to compute the transition probabilities in the Markov model.

Given that the risk equations for these events captured the time varying nature of the hazards (i.e. did not display constant hazards) we modelled the non-fatal primary endpoints as tunnel states. We

implemented our model with a 90 day cycle length and attached costs and utilities to the states in the model. The 90 day cycle length we felt gave a good trade-off between capturing the time varying hazards and the granularity of resource use captured.

The non-linear nature of our model meant that we needed to run it probabilistically and average over the results to capture the uncertainty in the model input parameters appropriately. We ran the model for 1,000 iterations for each patient profile and treatment scenario combination. For each simulation of the model the coefficients in the risk-equations, cost equations and HRQL equations were resampled and model results were computed. The average across these simulated results comprise the central estimate for each patient profile and treatment combination with the variance in these simulated results providing the confidence intervals around these results.

A number of assumptions were made in the modelling process these include:

(a) Only first events were explicitly modelled with recurrent event implicitly captured in the time varying nature of costs and risks following events
(b) We assume current estimates of event rates are valid as predictions of future event rates
(c) For simulation in the PSA we assign a multivariate normal distribution to the costs and beta and gamma distributions to the constant level and event specific decrements in HRQL respectively.
(d) The following parametric models were assigned to the risk equations to extrapolate them and multivariate normal distributions were used to simulate the coefficients from these equations in the PSA

**Risk Equation**	**Parametric Model**
Equation 1: Stable-CAD to MI	Weibull
Equation 2: Stable-CAD to Stroke I	Weibull
Equation 3: Stable-CAD to Stroke H	Exponential
Equation 4: Stable-CAD to Fatal CVD	Weibull
Equation 5: Stable-CAD to Fatal non-CVD	Weibull
Equation 6: MI to Fatal CVD	Log Normal
Equation 7: MI to Fatal non-CVD	Generalised Gamma
Equation 8: Stroke I to Fatal CVD	Generalised Gamma
Equation 9: Stroke I to Fatal non-CVD	Generalised Gamma
Equation 10: Stroke H to Fatal CVD	Log Normal
Equation 11: Stroke H to Fatal non-CVD	Weibull

The model was run for a range of different patient and population profiles and a range of indicative treatment effects. To handle the computational burden involved the N8 supercomputer was used to run all iterations and scenarios in parallel.

The full model code in R along with UNIX shell scripts to run the model in parallel on a sun grid engine supercomputer is available at: https://github.com/miqdadasaria/caliber-scad-model

To run the model for a new patient / population profile the following patient characteristics must be defined in a csv file, with one patient per row and headings following the variable name column:

Variable Name	Variable Description	Example Value Individual	Example Value Population
Sex	Female=1, Male=0	1	0.398146
IMD5	Whether person lives in most deprived fifth of LSOAs	TRUE	0.190781
dx7CHD	SCAD index event other CHD	FALSE	0
dx7NSTEMI	SCAD index event NSTEMI	TRUE	0.641551
dx7STEMI	SCAD index event STEMI	FALSE	0.358449
dx7UA	SCAD index event Unstable Angina	FALSE	0
earlyPCI	PCI in last 6 months	TRUE	0.231131
earlyCABG	CABG in last 6 months	FALSE	0.064769
recurrent_mi	Previous/recurrent MI	TRUE	0.267824
nitrates_long	Use of Nitrates		0.270175
Smcatcurrent	Current Smoker	FALSE	0.279943
Smcatex	Ex-Smoker	FALSE	0.354583
Hypertension	Hypertension	TRUE	0.680935
Diabetes	Diabetes	TRUE	0.220554
hist_hf	History of Heart failure	FALSE	0.279316
hist_pad	History of Peripheral arterial disease	TRUE	0.107208
hist_af	History of Atrial fibrillation	FALSE	0.196657
hist_stroke	History of Stroke	FALSE	0
hist_renal	History of Chronic kidney disease	FALSE	0.10982
hist_copd	History of Chronic obstructive pulmonary disease	FALSE	0.235962
hist_cancer	History of Cancer	TRUE	0.112562

hist_liver	History of Chronic liver disease	FALSE	0.010969
Depression	Depression at diagnosis	TRUE	0.141029
hist_anxiety	Anxiety at diagnosis	FALSE	0.073257
age0_ori	Age	70	75.1106
pulse_rate_ori	Heart rate (b.p.m.)	75	70.00349
HDL_ori	HDL (mmol/L)	1.4	1.32259
TCHOL_ori	Total cholesterol (mmol/L)	4.8	4.218232
CREAT_ori	Creatinine (mmol/L)	90	105.7011
WCC_ori	White cell count (10^9/L)	7	7.638091
HGB_ori	Haemoglobin (g/100ml)	14	13.27026
sex:age0	Average age difference between men and women in population	NA	3.239488

Where all the SCAD index events are set to false the index event is taken to be stable angina, where all the smoking status variables are set to false the smoking status is taken to be never smoked. Population level values for these sets of grouped variables including the excluded category must sum to 1.

The model is then run by calling: *"RScript run_model.R <patient> <iteration> manual <path to csv file>"* from the command line.

Where <patient> indicates the patient profile to select from the csv file starting from 1, <iteration> represents the PSA iteration that you want the model to run for ranging between 1 and 10,000 (this will reference pre-computed realisations from the underlying input parameter distributions), manual indicates that you want to provide patient information using a csv file other options here are deciles and clinical to load up the patient profiles used to generate the results in the paper, finally <path to csv file> indicates the path from the working directory to the file where the patient profiles have been saved.
