# Supplementary material for: Using electronic health records to predict costs and outcomes in stable coronary artery disease
Source: Heart. 2016 Feb 10;102(10):755–62. doi: 10.1136/heartjnl-2015-308850 (PMC4849559; doi:10.1136/heartjnl-2015-308850)
Supplement: Web appendix C [file heartjnl-2015-308850-s3.pdf]

Modelling lifetime costs and health outcomes for patients with stable coronary artery disease

Appendix C: Cost and Health Related Quality of Life Input Parameters

Panel data methods with time invariant covariates were used to estimate patient costs over each 90 day period. The costs for each individual in the CALIBER data set were calculated and partitioned into equal time periods, with the length of each period matching the 90 days cycle length of the model, to create a longitudinal data set of costs. Panel data estimates based on linear regression were then applied to estimate the costs for use in the model. Using these methods both an underlying background cost as well as an event specific cost for the events (captured using a dummy variable in the time period in which the event occurred) were calculated. Costs were adjusted based on important patient risk factors and co-morbidities to allow for the appropriate capturing of heterogeneity within the model. Aggregate costs were also estimated using the generalised linear model using a log link function to compare with the results generated from the linear model. Both methods gave similar results reassuring us that the linear model was appropriate to use for estimating costs for use in the model.

Mean costs were used in the model along with the cholesky decomposition of the estimated variance covariance matrix from the regression for use in the probabilistic sensitivity analysis.

Costs were assumed to follow a beta distribution.Costs were allocated to states in the model and adjusted for baseline co-variates as well as for patient age and time elapsed since previous non-fatal CVD event.

HRQL estimates were taken from the Sullivan et al (2011) catalogue. The uncertainty around the HRQL was inferred from the standard errors reported in the catalogue and was assumed to follow a gamma distribution.

As with costs the HRQL values were attached to model states and adjusted for baseline patient covariates and updated for patient age as patients progressed through the model.

Total Costs Mean Estimates

fatalCVD	fatalNONCVD	firsteventMI	MIdiabetes	firsteventMI2	MIdiabetes2	firsteventMI3	MIdiabetes3	firsteventMI4
2008	2240	5028	776	1282	1100	675	785	692

MIdiabetes4	feMI	feMIdiabetes	firsteventStroke_I	firsteventStroke_I2	firsteventStroke_I3	firsteventStroke_I4	feSTROKE_I	firsteventStroke_H
550	521	369	6215	1239	795	654	564	7011

firsteventStroke_H2	firsteventStroke_H3	firsteventStroke_H4	feSTROKE_H	age0	timeperiod	diabetes	hist_liver	hist_hf
1767	947	751	927	7	10	338	530	364

hist_af	hist_pad	hist_copd	hist_cancer	hist_renal	sex	CHD	NSTEMI	STEMI
186	327	231	331	756	-7	-20	157	-26

UA	_cons
153	341

Total Costs Cholesky Decomposition of Variance Covariance Matrix

	fatalCVD	fatalNONCVD	firsteventMI	MIdiabetes	firsteventMI2	MIdiabetes2	firsteventMI3	MIdiabetes3	firsteventMI4
fatalCVD	24.878								
fatalNONCVD	0.158	20.161							
firsteventMI	0.128	0.106	34.413						
MIdiabetes	0.019	0.045	-34.375	67.544					
firsteventMI2	-0.598	-0.417	3.408	0.020	36.673				
MIdiabetes2	-0.164	-0.051	-3.367	7.123	-36.638	72.441			
firsteventMI3	-0.276	-0.154	3.406	0.021	3.299	0.018	38.865		
MIdiabetes3	-0.099	-0.337	-3.361	7.102	-3.259	6.874	-38.830	77.096	
firsteventMI4	-0.245	-0.136	3.433	0.023	3.325	0.020	3.230	0.018	40.428
MIdiabetes4	-0.335	-0.057	-3.384	7.049	-3.281	6.823	-3.191	6.622	-40.392
feMI	-0.255	-0.194	3.554	0.040	3.443	0.037	3.345	0.035	3.214
feMIdiabetes	-0.131	-0.124	-3.471	7.076	-3.365	6.845	-3.273	6.641	-3.145
firsteventStroke_I	0.130	0.146	0.062	0.038	0.057	0.034	0.052	0.031	0.050
firsteventStroke_I2	-0.430	-0.498	0.066	0.040	0.061	0.036	0.057	0.033	0.055
firsteventStroke_I3	-0.279	-0.224	0.071	0.042	0.065	0.038	0.061	0.035	0.059
firsteventStroke_I4	-0.222	-0.239	0.075	0.044	0.070	0.040	0.066	0.037	0.064
feSTROKE_I	-0.277	-0.200	0.111	0.061	0.106	0.057	0.101	0.054	0.099
firsteventStroke_H	0.121	0.127	0.060	0.033	0.055	0.030	0.051	0.027	0.048
firsteventStroke_H2	-0.501	-0.572	0.061	0.034	0.056	0.031	0.053	0.029	0.051
firsteventStroke_H3	-0.703	-0.189	0.065	0.035	0.061	0.032	0.057	0.030	0.055
firsteventStroke_H4	0.003	-0.088	0.069	0.034	0.065	0.032	0.062	0.030	0.060
feSTROKE_H	-0.314	-0.153	0.106	0.050	0.101	0.048	0.097	0.046	0.095
age0	-0.012	-0.013	-0.003	-0.001	-0.002	-0.001	-0.001	-0.001	-0.001
timeperiod	-0.006	-0.008	-0.005	-0.002	-0.005	-0.002	-0.005	-0.002	-0.005
diabetes	-0.066	-0.025	0.196	-0.551	0.164	-0.468	0.141	-0.406	0.125
hist_liver	-0.087	-0.328	-0.026	-0.051	0.004	-0.023	-0.006	-0.013	-0.010
hist_hf	-0.271	-0.193	-0.035	-0.035	-0.023	-0.023	-0.017	-0.013	-0.012
hist_af	-0.098	-0.068	0.006	0.015	0.003	0.008	0.009	0.009	0.008
hist_pad	-0.146	-0.097	-0.077	-0.063	-0.063	-0.043	-0.052	-0.042	-0.046
hist_copd	0.003	-0.113	-0.023	-0.008	-0.020	-0.008	-0.013	-0.005	-0.013
hist_cancer	0.011	-0.470	-0.014	0.004	-0.011	0.005	-0.004	0.004	-0.002
hist_renal	-0.078	-0.066	-0.007	-0.046	-0.002	-0.018	0.002	-0.003	0.001
sex	0.071	0.063	0.040	0.016	0.034	0.011	0.030	0.011	0.026
CHD	-0.036	0.043	-0.047	-0.013	-0.040	-0.012	-0.038	-0.011	-0.035
NSTEMI	-0.220	-0.045	-0.240	-0.195	-0.219	-0.160	-0.191	-0.136	-0.170
STEMI	-0.025	-0.001	-0.171	-0.053	-0.157	-0.049	-0.145	-0.046	-0.131
UA	-0.048	-0.002	-0.083	-0.034	-0.068	-0.033	-0.063	-0.029	-0.057
_cons	-0.075	-0.075	-0.084	0.056	-0.061	0.051	-0.049	0.044	-0.038

	MIdiabetes4	feMI	feMIdiabetes	firsteventStroke_I	firsteventStroke_I2	firsteventStroke_I3	firsteventStroke_I4	feSTROKE_I	firsteventStroke_H
MIdiabetes4	80.331								
feMI	0.034	17.361							
feMIdiabetes	6.365	-17.053	35.516						
firsteventStroke_I	0.029	0.201	0.104	36.008					
firsteventStroke_I2	0.031	0.225	0.114	3.121	37.899				
firsteventStroke_I3	0.033	0.247	0.124	3.103	3.004	40.821			
firsteventStroke_I4	0.035	0.268	0.133	3.115	3.017	3.007	42.796		
feSTROKE_I	0.053	0.435	0.208	3.303	3.199	3.188	3.113	18.906	
firsteventStroke_H	0.026	0.192	0.093	0.062	0.058	0.053	0.051	0.177	125.411
firsteventStroke_H2	0.027	0.210	0.102	0.066	0.062	0.058	0.055	0.196	11.091
firsteventStroke_H3	0.029	0.232	0.111	0.070	0.066	0.062	0.060	0.216	10.719
firsteventStroke_H4	0.029	0.255	0.116	0.074	0.071	0.066	0.064	0.236	10.546
feSTROKE_H	0.046	0.420	0.188	0.114	0.111	0.105	0.102	0.385	11.324
age0	0.000	-0.002	-0.001	-0.004	-0.004	-0.003	-0.002	-0.006	-0.001
timeperiod	-0.002	-0.024	-0.011	-0.005	-0.006	-0.005	-0.005	-0.022	-0.001
diabetes	-0.359	0.309	-0.955	-0.040	-0.032	-0.029	-0.026	-0.061	-0.003
hist_liver	0.001	-0.001	-0.065	-0.032	-0.032	-0.030	-0.015	0.014	0.022
hist_hf	-0.009	-0.031	-0.039	-0.034	-0.025	-0.018	-0.017	-0.043	0.004
hist_af	0.009	0.018	0.031	-0.072	-0.057	-0.042	-0.035	-0.094	-0.034
hist_pad	-0.035	-0.099	-0.065	-0.045	-0.043	-0.034	-0.034	-0.089	0.006
hist_copd	-0.004	-0.035	0.003	0.003	0.005	0.001	-0.001	-0.007	0.003
hist_cancer	0.007	-0.006	0.024	0.016	0.006	0.004	0.006	0.004	0.013
hist_renal	-0.007	0.001	-0.004	0.025	0.018	0.023	0.023	0.030	-0.019
sex	0.008	0.079	0.032	0.009	0.007	0.007	0.005	0.017	0.015
CHD	-0.011	-0.079	-0.026	-0.002	-0.005	-0.004	-0.004	-0.016	0.000
NSTEMI	-0.128	-0.423	-0.352	-0.002	-0.008	-0.013	-0.012	-0.053	0.006
STEMI	-0.038	-0.314	-0.115	0.009	0.009	0.010	0.008	0.028	0.019
UA	-0.022	-0.142	-0.057	-0.021	-0.022	-0.021	-0.021	-0.065	-0.010
_cons	0.042	0.005	0.154	-0.049	-0.032	-0.022	-0.012	0.061	-0.027

	firsteventStroke_H2	firsteventStroke_H3	firsteventStroke_H4	feSTROKE_H	age0	timeperiod	diabetes	hist_liver	hist_hf
firsteventStroke_H2	138.357								
firsteventStroke_H3	10.863	154.464							
firsteventStroke_H4	10.687	11.061	164.086						
feSTROKE_H	11.475	11.876	11.772	70.462					
age0	-0.001	0.000	0.000	-0.001	0.418				
timeperiod	-0.001	-0.001	-0.001	-0.006	0.004	0.175			
diabetes	-0.006	-0.005	0.000	0.004	0.367	0.125	13.630		
hist_liver	0.028	0.022	0.018	0.043	1.366	0.231	-1.452	50.663	
hist_hf	0.002	0.001	0.005	0.018	-2.542	0.185	-0.901	-0.245	11.922
hist_af	-0.022	-0.021	-0.021	-0.072	-2.216	0.204	0.257	-0.073	-3.140
hist_pad	0.003	0.000	0.001	0.007	-1.075	0.107	-1.100	-0.095	-1.207
hist_copd	0.001	0.002	-0.001	-0.006	0.234	0.084	-0.165	-0.193	-1.349
hist_cancer	0.008	0.005	0.006	0.006	-2.419	0.237	0.058	-0.107	-0.673
hist_renal	-0.012	0.004	-0.002	0.001	-1.286	0.678	-2.117	-0.234	-2.576
sex	0.012	0.011	0.005	0.006	-1.768	0.003	0.346	0.137	-1.153
CHD	0.002	0.002	-0.003	-0.013	-0.929	-0.174	-0.194	0.167	-0.857
NSTEMI	0.023	0.017	0.014	0.014	-1.753	0.294	-0.696	-0.080	-0.939
STEMI	0.020	0.016	0.012	0.010	1.323	0.220	0.291	-0.058	1.167
UA	-0.005	0.001	0.004	-0.007	0.133	0.002	-0.156	-0.140	-0.694
_cons	-0.022	-0.017	-0.010	0.008	2.819	-2.025	-1.669	-0.386	-1.049

	hist_af	hist_pad	hist_copd	hist_cancer	hist_renal	sex	CHD	NSTEMI	STEMI
hist_af	13.788								
hist_pad	-0.637	17.674							
hist_copd	-0.360	-0.480	11.483						
hist_cancer	-0.760	-0.318	-0.311	17.301					
hist_renal	-1.453	-1.342	-0.619	-1.008	20.482				
sex	0.004	0.322	-0.511	-0.085	-0.128	9.839			
CHD	0.050	-0.637	0.255	-0.096	0.085	1.467	12.227		
NSTEMI	-1.260	-1.190	-0.180	-0.509	-1.422	1.143	3.999	17.602	
STEMI	0.910	0.342	0.640	0.515	0.185	2.672	3.999	-0.043	20.809
UA	-0.357	-0.569	-0.364	-0.098	-0.192	0.553	3.999	1.869	1.585
_cons	-1.637	-1.070	-2.398	-1.330	-1.035	-5.049	-3.999	-1.869	-1.584

	UA	_cons
UA	14.113	
_cons	-1.906	4.823

CVD Specific Costs Mean Estimates

fatalCVD	fatalNONCVD	firsteventMI	MIdiabetes	firsteventMI2	MIdiabetes2	firsteventMI3	MIdiabetes3
2071	1737	4854	674	1209	1042	640	660

firsteventMI4	MIdiabetes4	feMI	feMIdiabetes	firsteventStroke_I	firsteventStroke_I2	firsteventStroke_I3	firsteventStroke_I4
675	403	481	280	5957	1151	675	539

feSTROKE_I	firsteventStroke_H	firsteventStroke_H2	firsteventStroke_H3	firsteventStroke_H4	feSTROKE_H	age0	timeperiod
448	6836	1517	585	393	670	6	7

diabetes	hist_liver	hist_hf	hist_af	hist_pad	hist_copd	hist_cancer	hist_renal
194	279	248	221	242	142	154	418

sex	CHD	NSTEMI	STEMI	UA	_cons
-23	2	145	29	125	224

CVD Specific Costs Cholesky Decomposition of Variance Covariance Matrix

	fatalCVD	fatalNONCVD	firsteventMI	MIdiabetes	firsteventMI2	MIdiabetes2	firsteventMI3	MIdiabetes3
fatalCVD	21.298							
fatalNONCVD	0.142	17.279						
firsteventMI	0.110	0.097	29.547					
MIdiabetes	0.020	0.039	-29.515	57.965				
firsteventMI2	-0.513	-0.353	2.720	0.016	31.513			
MIdiabetes2	-0.138	-0.044	-2.686	5.659	-31.484	62.225		
firsteventMI3	-0.231	-0.125	2.722	0.017	2.654	0.015	33.415	
MIdiabetes3	-0.081	-0.288	-2.685	5.648	-2.621	5.508	-33.386	66.266
firsteventMI4	-0.204	-0.109	2.743	0.019	2.675	0.016	2.613	0.015
MIdiabetes4	-0.283	-0.045	-2.703	5.611	-2.639	5.472	-2.581	5.343
feMI	-0.210	-0.158	2.817	0.032	2.748	0.030	2.685	0.028
feMIdiabetes	-0.110	-0.100	-2.751	5.577	-2.686	5.434	-2.626	5.304
firsteventStroke_I	0.114	0.127	0.055	0.035	0.050	0.031	0.046	0.028
firsteventStroke_I2	-0.367	-0.425	0.059	0.036	0.054	0.033	0.050	0.030
firsteventStroke_I3	-0.235	-0.188	0.062	0.038	0.057	0.034	0.053	0.031
firsteventStroke_I4	-0.184	-0.199	0.065	0.039	0.061	0.036	0.057	0.033
feSTROKE_I	-0.230	-0.163	0.094	0.053	0.090	0.050	0.086	0.047
firsteventStroke_H	0.106	0.112	0.054	0.030	0.049	0.027	0.045	0.024
firsteventStroke_H2	-0.429	-0.488	0.054	0.030	0.049	0.028	0.046	0.025
firsteventStroke_H3	-0.600	-0.156	0.057	0.031	0.053	0.028	0.049	0.027
firsteventStroke_H4	0.009	-0.070	0.060	0.029	0.056	0.028	0.053	0.026
feSTROKE_H	-0.261	-0.126	0.090	0.042	0.086	0.041	0.082	0.039
age0	-0.010	-0.011	-0.002	-0.001	-0.002	-0.001	-0.001	-0.001
timeperiod	-0.004	-0.007	-0.004	-0.002	-0.004	-0.002	-0.004	-0.002
diabetes	-0.053	-0.021	0.162	-0.454	0.137	-0.389	0.118	-0.340
hist_liver	-0.067	-0.261	-0.020	-0.043	0.003	-0.020	-0.005	-0.011
hist_hf	-0.216	-0.155	-0.029	-0.028	-0.019	-0.019	-0.014	-0.011
hist_af	-0.079	-0.054	0.005	0.012	0.003	0.006	0.008	0.007
hist_pad	-0.116	-0.078	-0.065	-0.053	-0.053	-0.037	-0.044	-0.036
hist_copd	0.002	-0.091	-0.019	-0.007	-0.017	-0.007	-0.011	-0.004
hist_cancer	0.010	-0.369	-0.012	0.003	-0.009	0.003	-0.003	0.002
hist_renal	-0.066	-0.056	-0.007	-0.040	-0.003	-0.016	0.001	-0.003
sex	0.057	0.051	0.033	0.013	0.028	0.009	0.025	0.009
CHD	-0.029	0.034	-0.039	-0.011	-0.034	-0.010	-0.032	-0.009
NSTEMI	-0.173	-0.038	-0.198	-0.154	-0.182	-0.127	-0.160	-0.109
STEMI	-0.025	-0.001	-0.143	-0.051	-0.133	-0.046	-0.122	-0.043
UA	-0.038	-0.002	-0.068	-0.028	-0.057	-0.027	-0.052	-0.024
_cons	-0.060	-0.061	-0.071	0.045	-0.052	0.042	-0.042	0.036

	firsteventMI4	MIdiabetes4	feMI	feMIdiabetes	firsteventStroke_I	firsteventStroke_I2	firsteventStroke_I3	firsteventStroke_I4
firsteventMI4	34.772							
MIdiabetes4	-34.742	69.080						
feMI	2.592	0.028	14.664					
feMIdiabetes	-2.535	5.109	-14.406	29.956				
firsteventStroke_I	0.044	0.027	0.174	0.093	30.944			
firsteventStroke_I2	0.048	0.028	0.194	0.101	2.515	32.590		
firsteventStroke_I3	0.052	0.030	0.213	0.109	2.504	2.438	35.117	
firsteventStroke_I4	0.055	0.032	0.230	0.117	2.516	2.449	2.451	36.826
feSTROKE_I	0.084	0.046	0.371	0.180	2.652	2.583	2.585	2.534
firsteventStroke_H	0.043	0.023	0.166	0.082	0.056	0.052	0.047	0.045
firsteventStroke_H2	0.044	0.024	0.181	0.089	0.059	0.055	0.051	0.048
firsteventStroke_H3	0.048	0.025	0.200	0.096	0.061	0.058	0.054	0.052
firsteventStroke_H4	0.052	0.025	0.219	0.099	0.065	0.062	0.058	0.056
feSTROKE_H	0.081	0.039	0.358	0.160	0.097	0.094	0.089	0.087
age0	-0.001	0.000	-0.002	-0.001	-0.004	-0.003	-0.002	-0.002
timeperiod	-0.004	-0.002	-0.020	-0.009	-0.004	-0.005	-0.005	-0.005
diabetes	0.105	-0.302	0.264	-0.812	-0.034	-0.027	-0.025	-0.022
hist_liver	-0.008	0.001	0.001	-0.055	-0.028	-0.027	-0.026	-0.013
hist_hf	-0.010	-0.007	-0.026	-0.033	-0.029	-0.021	-0.015	-0.015
hist_af	0.007	0.007	0.015	0.026	-0.061	-0.048	-0.036	-0.030
hist_pad	-0.039	-0.030	-0.085	-0.056	-0.037	-0.036	-0.029	-0.028
hist_copd	-0.011	-0.004	-0.030	0.002	0.003	0.004	0.000	-0.001
hist_cancer	-0.002	0.006	-0.006	0.019	0.012	0.004	0.004	0.004
hist_renal	0.000	-0.007	-0.001	-0.002	0.020	0.015	0.019	0.019
sex	0.022	0.007	0.068	0.028	0.007	0.005	0.005	0.004
CHD	-0.029	-0.009	-0.068	-0.022	-0.002	-0.004	-0.003	-0.003
NSTEMI	-0.143	-0.103	-0.359	-0.289	-0.003	-0.007	-0.011	-0.010
STEMI	-0.111	-0.036	-0.270	-0.107	0.008	0.008	0.009	0.007
UA	-0.048	-0.019	-0.121	-0.047	-0.018	-0.019	-0.018	-0.018
_cons	-0.032	0.035	0.005	0.131	-0.042	-0.028	-0.019	-0.011

	feSTROKE_I	firsteventStroke_H	firsteventStroke_H2	firsteventStroke_H3	firsteventStroke_H4	feSTROKE_H	age0	timeperiod
feSTROKE_I	16.041							
firsteventStroke_H	0.154	107.730						
firsteventStroke_H2	0.170	8.894	118.948					
firsteventStroke_H3	0.186	8.635	8.806	132.879				
firsteventStroke_H4	0.203	8.510	8.677	9.024	141.206			
feSTROKE_H	0.329	9.080	9.259	9.628	9.580	59.812		
age0	-0.005	-0.001	-0.001	0.000	0.000	-0.001	0.318	
timeperiod	-0.019	-0.001	-0.001	-0.001	-0.001	-0.005	0.004	0.150
diabetes	-0.053	-0.003	-0.005	-0.004	0.000	0.003	0.270	0.108
hist_liver	0.012	0.018	0.023	0.019	0.015	0.037	1.037	0.196
hist_hf	-0.037	0.003	0.001	0.000	0.004	0.015	-1.926	0.157
hist_af	-0.080	-0.029	-0.018	-0.018	-0.018	-0.062	-1.686	0.174
hist_pad	-0.076	0.005	0.003	0.000	0.001	0.007	-0.824	0.092
hist_copd	-0.006	0.002	0.001	0.002	-0.001	-0.005	0.174	0.071
hist_cancer	0.003	0.010	0.007	0.004	0.005	0.006	-1.840	0.201
hist_renal	0.025	-0.016	-0.010	0.003	-0.002	0.001	-0.966	0.586
sex	0.014	0.012	0.010	0.010	0.004	0.005	-1.343	0.004
CHD	-0.014	0.000	0.002	0.002	-0.002	-0.011	-0.704	-0.148
NSTEMI	-0.043	0.006	0.019	0.013	0.011	0.010	-1.207	0.246
STEMI	0.021	0.016	0.018	0.015	0.011	0.011	0.844	0.195
UA	-0.056	-0.009	-0.004	0.001	0.003	-0.006	0.104	0.002
_cons	0.053	-0.023	-0.019	-0.014	-0.009	0.007	2.143	-1.759

	diabetes	hist_liver	hist_hf	hist_af	hist_pad	hist_copd	hist_cancer	hist_renal
diabetes	10.359							
hist_liver	-1.090	38.645						
hist_hf	-0.685	-0.185	9.058					
hist_af	0.194	-0.055	-2.389	10.493				
hist_pad	-0.834	-0.072	-0.921	-0.487	13.439			
hist_copd	-0.127	-0.146	-1.022	-0.271	-0.363	8.719		
hist_cancer	0.043	-0.078	-0.512	-0.576	-0.243	-0.236	13.180	
hist_renal	-1.617	-0.174	-1.970	-1.103	-1.024	-0.476	-0.766	15.727
sex	0.260	0.102	-0.878	0.003	0.238	-0.391	-0.072	-0.096
CHD	-0.147	0.127	-0.651	0.038	-0.484	0.195	-0.074	0.065
NSTEMI	-0.501	-0.071	-0.633	-0.883	-0.841	-0.107	-0.341	-1.003
STEMI	0.187	-0.033	0.781	0.595	0.179	0.455	0.336	0.058
UA	-0.118	-0.105	-0.529	-0.270	-0.434	-0.275	-0.074	-0.145
_cons	-1.263	-0.292	-0.786	-1.236	-0.807	-1.815	-1.001	-0.774

	sex	CHD	NSTEMI	STEMI	UA	_cons
sex	7.461					
CHD	1.116	9.259				
NSTEMI	0.936	3.035	13.090			
STEMI	1.947	3.035	0.842	15.259		
UA	0.423	3.035	1.443	1.158	10.706	
_cons	-3.830	-3.035	-1.443	-1.158	-1.445	3.658

CHD Specific Costs Mean Estimates

fatalCVD	fatalNONCVD	firsteventMI	MIdiabetes	firsteventMI2	MIdiabetes2	firsteventMI3	MIdiabetes3
1407	1068	4658	643	1166	792	590	701

firsteventMI4	MIdiabetes4	feMI	feMIdiabetes	firsteventStroke_I	firsteventStroke_I2	firsteventStroke_I3	firsteventStroke_I4
642	330	475	269	3029	620	415	262

feSTROKE_I	firsteventStroke_H	firsteventStroke_H2	firsteventStroke_H3	firsteventStroke_H4	feSTROKE_H	age0	timeperiod
256	2874	790	218	301	251	4	4

diabetes	hist_liver	hist_hf	hist_af	hist_pad	hist_copd	hist_cancer	hist_renal
144	198	143	84	161	117	107	201

sex	CHD	NSTEMI	STEMI	UA	_cons
-23	82	219	111	163	179

CHD Specific Costs Cholesky Decomposition of Variance Covariance Matrix

	fatalCVD	fatalNONCVD	firsteventMI	MIdiabetes	firsteventMI2	MIdiabetes2	firsteventMI3	MIdiabetes3
fatalCVD	17.056							
fatalNONCVD	0.116	13.843						
firsteventMI	0.088	0.079	23.686					
MIdiabetes	0.017	0.031	-23.661	46.458				
firsteventMI2	-0.411	-0.281	2.109	0.013	25.271			
MIdiabetes2	-0.109	-0.035	-2.082	4.379	-25.247	49.891		
firsteventMI3	-0.184	-0.098	2.111	0.014	2.064	0.012	26.802	
MIdiabetes3	-0.064	-0.230	-2.082	4.373	-2.039	4.278	-26.779	53.145
firsteventMI4	-0.161	-0.085	2.128	0.015	2.081	0.013	2.038	0.012
MIdiabetes4	-0.225	-0.035	-2.096	4.346	-2.053	4.251	-2.012	4.162
feMI	-0.165	-0.124	2.177	0.025	2.130	0.023	2.086	0.022
feMIdiabetes	-0.088	-0.079	-2.126	4.300	-2.081	4.203	-2.040	4.112
firsteventStroke_I	0.092	0.103	0.045	0.029	0.041	0.026	0.038	0.023
firsteventStroke_I2	-0.294	-0.340	0.048	0.030	0.044	0.027	0.040	0.024
firsteventStroke_I3	-0.187	-0.149	0.050	0.031	0.046	0.028	0.043	0.026
firsteventStroke_I4	-0.146	-0.158	0.053	0.032	0.049	0.029	0.046	0.027
feSTROKE_I	-0.181	-0.128	0.075	0.043	0.072	0.040	0.068	0.038
firsteventStroke_H	0.085	0.091	0.044	0.024	0.040	0.022	0.036	0.020
firsteventStroke_H2	-0.343	-0.391	0.044	0.025	0.040	0.022	0.037	0.021
firsteventStroke_H3	-0.480	-0.123	0.046	0.025	0.042	0.023	0.040	0.021
firsteventStroke_H4	0.009	-0.055	0.048	0.024	0.045	0.022	0.043	0.021
feSTROKE_H	-0.207	-0.099	0.071	0.033	0.068	0.032	0.065	0.031
age0	-0.008	-0.009	-0.002	-0.001	-0.001	-0.001	-0.001	0.000
timeperiod	-0.003	-0.005	-0.003	-0.001	-0.003	-0.001	-0.003	-0.001
diabetes	-0.042	-0.016	0.128	-0.357	0.108	-0.307	0.094	-0.269
hist_liver	-0.051	-0.203	-0.015	-0.034	0.003	-0.016	-0.004	-0.009
hist_hf	-0.168	-0.121	-0.023	-0.022	-0.015	-0.015	-0.011	-0.008
hist_af	-0.062	-0.042	0.004	0.009	0.002	0.005	0.006	0.005
hist_pad	-0.090	-0.061	-0.051	-0.042	-0.042	-0.030	-0.035	-0.029
hist_copd	0.001	-0.071	-0.015	-0.005	-0.013	-0.006	-0.009	-0.004
hist_cancer	0.008	-0.285	-0.009	0.002	-0.007	0.002	-0.003	0.002
hist_renal	-0.053	-0.044	-0.006	-0.032	-0.002	-0.013	0.001	-0.003
sex	0.044	0.040	0.026	0.010	0.022	0.007	0.020	0.007
CHD	-0.023	0.027	-0.031	-0.009	-0.027	-0.008	-0.025	-0.007
NSTEMI	-0.136	-0.031	-0.157	-0.120	-0.145	-0.100	-0.128	-0.086
STEMI	-0.018	0.001	-0.112	-0.041	-0.104	-0.038	-0.097	-0.035
UA	-0.030	-0.002	-0.054	-0.022	-0.045	-0.022	-0.042	-0.019
_cons	-0.047	-0.048	-0.056	0.035	-0.042	0.033	-0.034	0.029

	firsteventMI4	MIdiabetes4	feMI	feMIdiabetes	firsteventStroke_I	firsteventStroke_I2	firsteventStroke_I3	firsteventStroke_I4
firsteventMI4	27.894							
MIdiabetes4	-27.871	55.414						
feMI	2.018	0.022	11.667					
feMIdiabetes	-1.974	3.970	-11.462	23.821				
firsteventStroke_I	0.036	0.022	0.140	0.076	24.816			
firsteventStroke_I2	0.039	0.023	0.156	0.082	1.958	26.142		
firsteventStroke_I3	0.042	0.024	0.171	0.089	1.951	1.903	28.173	
firsteventStroke_I4	0.044	0.026	0.185	0.095	1.960	1.912	1.918	29.548
feSTROKE_I	0.067	0.037	0.297	0.145	2.061	2.012	2.018	1.981
firsteventStroke_H	0.035	0.019	0.134	0.066	0.046	0.042	0.038	0.036
firsteventStroke_H2	0.036	0.020	0.145	0.072	0.048	0.045	0.041	0.039
firsteventStroke_H3	0.039	0.021	0.160	0.078	0.050	0.047	0.044	0.042
firsteventStroke_H4	0.041	0.020	0.175	0.080	0.052	0.050	0.046	0.045
feSTROKE_H	0.065	0.031	0.286	0.127	0.078	0.075	0.071	0.069
age0	-0.001	0.000	-0.001	-0.001	-0.003	-0.002	-0.002	-0.002
timeperiod	-0.003	-0.002	-0.016	-0.007	-0.003	-0.004	-0.004	-0.004
diabetes	0.084	-0.240	0.212	-0.649	-0.027	-0.022	-0.020	-0.018
hist_liver	-0.006	0.001	0.002	-0.044	-0.022	-0.022	-0.021	-0.010
hist_hf	-0.008	-0.006	-0.020	-0.026	-0.022	-0.016	-0.012	-0.012
hist_af	0.005	0.006	0.013	0.021	-0.048	-0.038	-0.028	-0.024
hist_pad	-0.031	-0.024	-0.068	-0.045	-0.029	-0.028	-0.023	-0.023
hist_copd	-0.009	-0.003	-0.023	0.001	0.002	0.003	0.000	-0.001
hist_cancer	-0.002	0.004	-0.005	0.015	0.009	0.003	0.003	0.004
hist_renal	0.000	-0.005	-0.001	-0.001	0.016	0.011	0.015	0.015
sex	0.017	0.005	0.054	0.022	0.005	0.004	0.004	0.003
CHD	-0.023	-0.007	-0.054	-0.018	-0.002	-0.003	-0.002	-0.002
NSTEMI	-0.114	-0.081	-0.291	-0.227	-0.004	-0.007	-0.010	-0.009
STEMI	-0.089	-0.030	-0.215	-0.089	0.009	0.008	0.009	0.007
UA	-0.038	-0.015	-0.097	-0.037	-0.014	-0.015	-0.014	-0.014
_cons	-0.026	0.028	0.004	0.105	-0.033	-0.022	-0.015	-0.009

	feSTROKE_I	firsteventStroke_H	firsteventStroke_H2	firsteventStroke_H3	firsteventStroke_H4	feSTROKE_H	age0	timeperiod
feSTROKE_I	12.788							
firsteventStroke_H	0.124	86.379						
firsteventStroke_H2	0.137	6.909	95.405					
firsteventStroke_H3	0.149	6.721	6.872	106.606				
firsteventStroke_H4	0.162	6.627	6.776	7.062	113.304			
feSTROKE_H	0.263	7.052	7.210	7.513	7.489	47.692		
age0	-0.004	-0.001	-0.001	0.000	0.000	-0.001	0.243	
timeperiod	-0.015	-0.001	-0.001	-0.001	-0.001	-0.004	0.003	0.120
diabetes	-0.042	-0.002	-0.004	-0.003	0.000	0.002	0.206	0.086
hist_liver	0.010	0.014	0.018	0.015	0.012	0.030	0.791	0.157
hist_hf	-0.029	0.003	0.001	0.000	0.003	0.012	-1.467	0.124
hist_af	-0.064	-0.023	-0.014	-0.014	-0.015	-0.050	-1.285	0.139
hist_pad	-0.060	0.003	0.002	0.000	0.001	0.006	-0.630	0.073
hist_copd	-0.005	0.002	0.001	0.001	-0.001	-0.005	0.134	0.057
hist_cancer	0.002	0.008	0.005	0.003	0.004	0.005	-1.405	0.159
hist_renal	0.020	-0.013	-0.008	0.002	-0.002	0.001	-0.731	0.470
sex	0.011	0.010	0.008	0.008	0.004	0.004	-1.024	0.003
CHD	-0.011	0.000	0.002	0.002	-0.002	-0.009	-0.538	-0.119
NSTEMI	-0.037	0.005	0.015	0.011	0.010	0.014	-0.935	0.203
STEMI	0.019	0.012	0.014	0.011	0.008	0.002	0.653	0.150
UA	-0.045	-0.007	-0.003	0.001	0.003	-0.005	0.080	0.001
_cons	0.043	-0.018	-0.015	-0.012	-0.007	0.006	1.635	-1.416

	diabetes	hist_liver	hist_hf	hist_af	hist_pad	hist_copd	hist_cancer	hist_renal
sex	5.701							
CHD	0.854	7.068						
NSTEMI	0.708	2.320	10.054					
STEMI	1.499	2.320	0.626	11.643				
UA	0.324	2.320	1.096	0.887	8.179			
_cons	-2.927	-2.320	-1.096	-0.887	-1.104	2.795		

Health Related Quality of Life

	_cons	age	male	acute_mi	old_mi	angina	hf	stroke
mean	0.8280	-0.0003	0.0010	-0.0626	-0.0368	-0.0854	-0.1167	-0.1171
standard error	0.0015	0.0002	0.0006	0.0132	0.0257	0.0134	0.0144	0.0121

Variable names and defiitions

Event costs

fatalCVD	Cost of a fatal cardiovascular event
fatalNONCVD	Cost of a fatal noncardiovascular event
firsteventMI	Cost of myocardial infarction in first quarter following event
MIdiabetes	Additional cost of myocardial infarction in first quarter for patients with diabetes
firsteventMI2	Cost of myocardial infarction in second quarter following event
MIdiabetes2	Additional cost of myocardial infarction in second quarter for patients with diabetes
firsteventMI3	Cost of myocardial infarction in third quarter following event
MIdiabetes3	Additional cost of myocardial infarction in second quarter for patients with diabetes
firsteventMI4	Cost of myocardial infarction in fourth quarter following event
MIdiabetes4	Additional cost of myocardial infarction in fourth quarter for patients with diabetes
feMI	Cost of myocardial infarction in all subsequent quarters following event
feMIdiabetes	Additional cost of myocardial infarction in all subsequent for patients with diabetes
firsteventStroke_I	Cost of ischemic stroke in first quarter following event
firsteventStroke_I2	Cost of ischemic stroke in second quarter following event
firsteventStroke_I3	Cost of ischemic stroke in third quarter following event
firsteventStroke_I4	Cost of ischemic stroke in fourth quarter following event
feSTROKE_I	Cost of ischemic stroke in all subsequent quarters following event
firsteventStroke_H	Cost of hemorrhagic stroke in first quarter following event
firsteventStroke_H2	Cost of hemorrhagic stroke in second quarter following event
firsteventStroke_H3	Cost of hemorrhagic stroke in third quarter following event
firsteventStroke_H4	Cost of hemorrhagic stroke in fourth quarter following event
feSTROKE_H	Cost of hemorrhagic stroke in all subsequent quarters following event

Background cost coefficients for quarter costs

age0	Baseline age
timeperiod	Model cycle number
diabetes	History of diabetes
hist_liver	History of liver disease
hist_hf	History of heart failure
hist_af	History of atrial fibrillation
hist_pad	History of peripheral artery disease
hist_copd	History of chronic obstructive pulmonary disease
hist_cancer	History of cancer
hist_renal	History of renal disease
sex	Female
CHD	Other CHD
NSTEMI	NSTEMI
STEMI	STEMI
UA	Unstable Angina
_cons	Constant
