# Supplementary material for: Using electronic health records to predict costs and outcomes in stable coronary artery disease
Source: Heart. 2016 Feb 10;102(10):755–62. doi: 10.1136/heartjnl-2015-308850 (PMC4849559; doi:10.1136/heartjnl-2015-308850)

Modelling lifetime costs and health outcomes for patients with stable coronary artery disease

Appendix D: Modelling and Selection of Risk Equations

The prognostic factors used in the risk equations as covariates were taken from the work of Rapsomaniki, Eleni, et al. "Prognostic models for stable coronary artery disease based on electronic health record cohort of 102 023 patients." European heart journal 35.13 (2014): 844-852. This study compares different prognostic models using the CALIBER dataset and develops a model to best exploit the unique properties of this dataset. We also follow this study in terms of the imputation model used to impute missing covariate values as detailed in the technical appendix to that study:
http://eurheartj.oxfordjournals.org/content/ehj/suppl/2013/12/01/eht533.DC1/eht533supp1.pdf

In this study we use these prognostic factors to fit a range of parametric survival models to each of the 11 risk equations in our model. We calculate hazards and survival over the time period we need to extrapolate our model over for every patient in the dataset and plot average values of these for each parametric model. These average predictions were used to assess clinical plausibility of the extrapolation made. The plots also contain piecewise exponentials for the hazards and Kaplan Meir estimates for survival to allow us to visually compare observed event rates to those predicted by averaging our parametric equations.

We also use the Akaike information criteria (AIC) to assess the goodness of fit of the various different parametric survival models to the observed data. The parametric model which has the best performance on the AIC is highlighted in red for each equation. These "best" performing models across the 11 equations were all deemed to be plausible extrapolations by our clinical experts and were combined in a competing risks framework using the methods proposed by Putter, H., M. Fiocco, and R. B. Geskus. "Tutorial in biostatistics: competing risks and multi-state models." Statistics in medicine 26.11 (2007): 2389.

The competing risks model was used to estimate patient specific time dependent transition probabilities for the Markov model described in appendix (b) by using the patient specific prognostic factors as covariates in the 11 risk equations to generate appropriate cumulative incidence functions from which transition probabilities could be derived.

The variance covariances matrices from the estimated models for the risk equations were used in the probabilistic sensitvity analysis of the model to account for the non-linearities in the model and characterise the uncertainty around the model estimates.

Equation 1: FE MI	GenGamma	LogNormal	Weibull	Exponential
**Sociodemographic characteristics**				
Age in men	0.98 (0.96-0.99)	0.98 (0.98-0.99)	0.98 (0.98-0.98)	0.98 (0.98-0.99)
Age in women	0.98 (0.97-1.00)	0.98 (0.98-0.99)	0.98 (0.98-0.99)	0.99 (0.98-0.99)
Women vs men	1.59 (1.19-2.14)	1.49 (1.32-1.68)	1.44 (1.29-1.59)	1.35 (1.24-1.46)
Most deprived quintile, yes vs. no	0.89 (0.62-1.27)	0.81 (0.73-0.89)	0.85 (0.78-0.93)	0.88 (0.82-0.94)
**SCAD diagnosis and severity**				
Other CHD vs. stable angina	0.89 (0.69-1.13)	0.85 (0.75-0.96)	0.79 (0.71-0.89)	0.84 (0.77-0.92)
NSTEMI vs. stable angina	0.19 (0.14-0.26)	0.19 (0.17-0.22)	0.23 (0.20-0.26)	0.31 (0.28-0.34)
STEMI vs stable angina	0.26 (0.19-0.36)	0.26 (0.22-0.32)	0.29 (0.25-0.35)	0.37 (0.33-0.43)
Unstable angina vs. stable angina	0.58 (0.47-0.71)	0.57 (0.50-0.65)	0.56 (0.50-0.63)	0.64 (0.58-0.70)
PCI in last 6 months	1.20 (0.77-1.87)	1.13 (0.97-1.32)	1.11 (0.97-1.27)	1.05 (0.95-1.17)
CABG in last 6 months	3.81 (1.90-7.62)	3.05 (2.39-3.91)	2.88 (2.28-3.65)	2.37 (1.97-2.85)
Previous/recurrent MI	0.53 (0.46-0.62)	0.57 (0.51-0.63)	0.62 (0.56-0.68)	0.69 (0.64-0.74)
Use of nitrates	0.62 (0.52-0.73)	0.64 (0.59-0.71)	0.69 (0.64-0.75)	0.75 (0.70-0.80)
**CVD risk factors**				
Current smoker vs. never	0.85 (0.65-1.10)	0.80 (0.69-0.92)	0.85 (0.75-0.97)	0.91 (0.83-1.01)
Ex-smoker vs. never	0.91 (0.68-1.21)	0.91 (0.79-1.05)	0.92 (0.81-1.05)	0.94 (0.85-1.04)
Hypertension	1.34 (0.85-2.10)	1.19 (1.07-1.32)	1.15 (1.05-1.27)	1.12 (1.04-1.21)
Diabetes mellitus	0.63 (0.44-0.91)	0.60 (0.54-0.67)	0.63 (0.57-0.70)	0.69 (0.64-0.75)
Total cholesterol, per 1 mmol/L increase	0.88 (0.76-1.03)	0.91 (0.86-0.96)	0.92 (0.87-0.97)	0.94 (0.90-0.98)
HDL, per 0.5 mmol/L increase	1.20 (0.98-1.47)	1.11 (1.04-1.18)	1.10 (1.04-1.17)	1.08 (1.03-1.13)
**CVD co-morbidities**				
Heart failure	0.86 (0.70-1.05)	0.86 (0.77-0.95)	0.86 (0.79-0.95)	0.88 (0.82-0.94)
Peripheral arterial disease	0.72 (0.41-1.28)	0.62 (0.54-0.71)	0.64 (0.57-0.71)	0.69 (0.63-0.75)
Atrial fibrillation	1.13 (0.84-1.51)	1.03 (0.92-1.17)	1.00 (0.90-1.11)	0.98 (0.90-1.06)
Stroke	0.77 (0.60-0.99)	0.79 (0.69-0.90)	0.80 (0.71-0.90)	0.82 (0.75-0.90)
**Non-CVD co-morbidities**				
Chornic kidney disease	0.84 (0.41-1.70)	0.97 (0.81-1.16)	0.90 (0.77-1.05)	0.84 (0.74-0.94)
Chronic obstructive pulmonary disease	0.91 (0.73-1.13)	0.85 (0.77-0.94)	0.86 (0.78-0.94)	0.88 (0.82-0.94)
Cancer	0.93 (0.70-1.25)	0.96 (0.83-1.11)	0.96 (0.84-1.09)	0.95 (0.86-1.05)
Chronic liver disease	0.83 (0.26-2.59)	0.77 (0.51-1.15)	0.75 (0.53-1.07)	0.78 (0.59-1.02)
**Psychosocial characteristics**				
Depression at diagnosis	1.16 (1.00-1.35)	1.15 (1.02-1.29)	1.11 (0.99-1.24)	1.06 (0.97-1.15)
Anxiety at diagnosis	1.11 (0.79-1.58)	1.04 (0.88-1.22)	1.04 (0.90-1.21)	1.02 (0.91-1.15)
**Biomarkers**				
Heart rate, per 10 b.p.m. increase	1.02 (0.96-1.08)	1.00 (0.95-1.05)	0.99 (0.95-1.04)	0.99 (0.95-1.03)
Creatinine, per 30 micromol/L increase	0.88 (0.77-1.00)	0.89 (0.85-0.94)	0.91 (0.87-0.95)	0.93 (0.90-0.96)
White cell count, per 1.5 10^9/L increase	0.89 (0.82-0.97)	0.89 (0.85-0.93)	0.90 (0.87-0.93)	0.92 (0.89-0.94)
Haemoglobin, per 1.5 g/dL increase	1.21 (1.09-1.34)	1.17 (1.10-1.25)	1.15 (1.08-1.21)	1.12 (1.07-1.17)
**Generalised gamma model parameters**				
mu	13.24 (12.32-14.16)	12.79 (12.59-12.99)	11.83 (11.65-12.00)	10.90 (10.80-11.00)
sigma	3.67 (2.27-5.95)	2.91 (2.84-2.97)	1.28 (1.24-1.31)	1
Q	-0.23 (-0.77-0.31)	0	1	1
**Model Fit**				
Log-likelihood	-52068.90	-51904.95	-51904.60	-52099.34
AIC	104207.81	103877.90	103877.20	104264.68

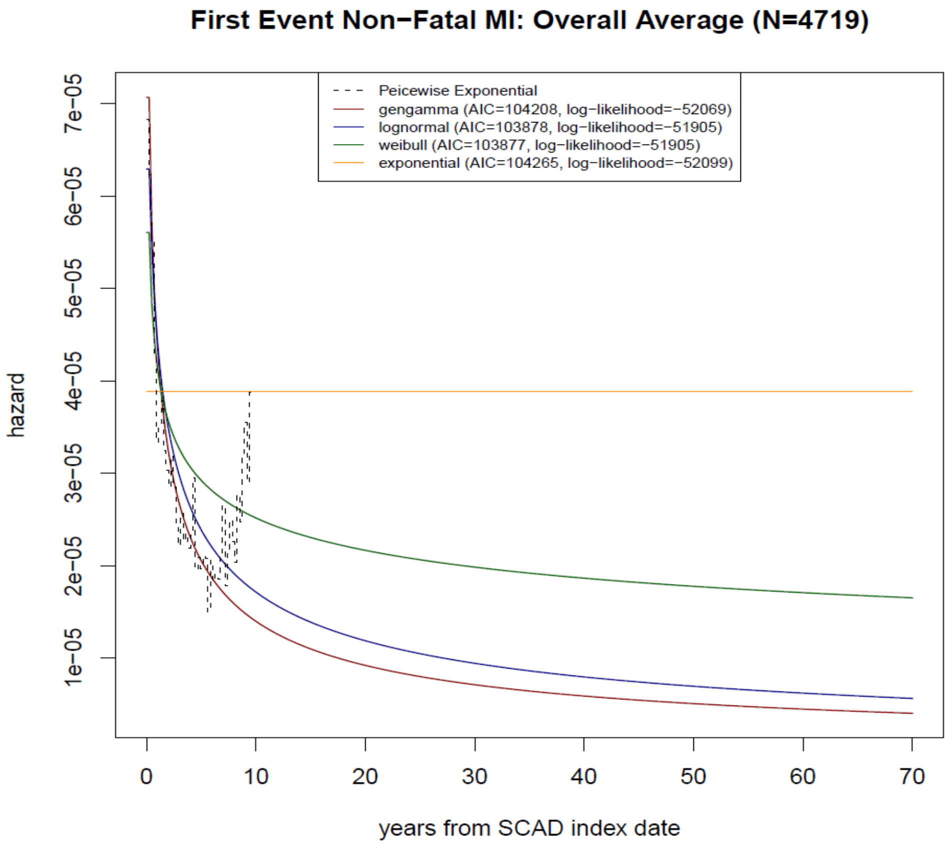
First Event Non−Fatal MI: Overall Average (N=4719)
Peicewise Exponential
gengamma (AIC=104208, log−likelihood=−52069)
lognormal (AIC=103878, log−likelihood=−51905)
weibull (AIC=103877, log−likelihood=−51905)
exponential (AIC=104265, log−likelihood=−52099)
hazard
years from SCAD index date

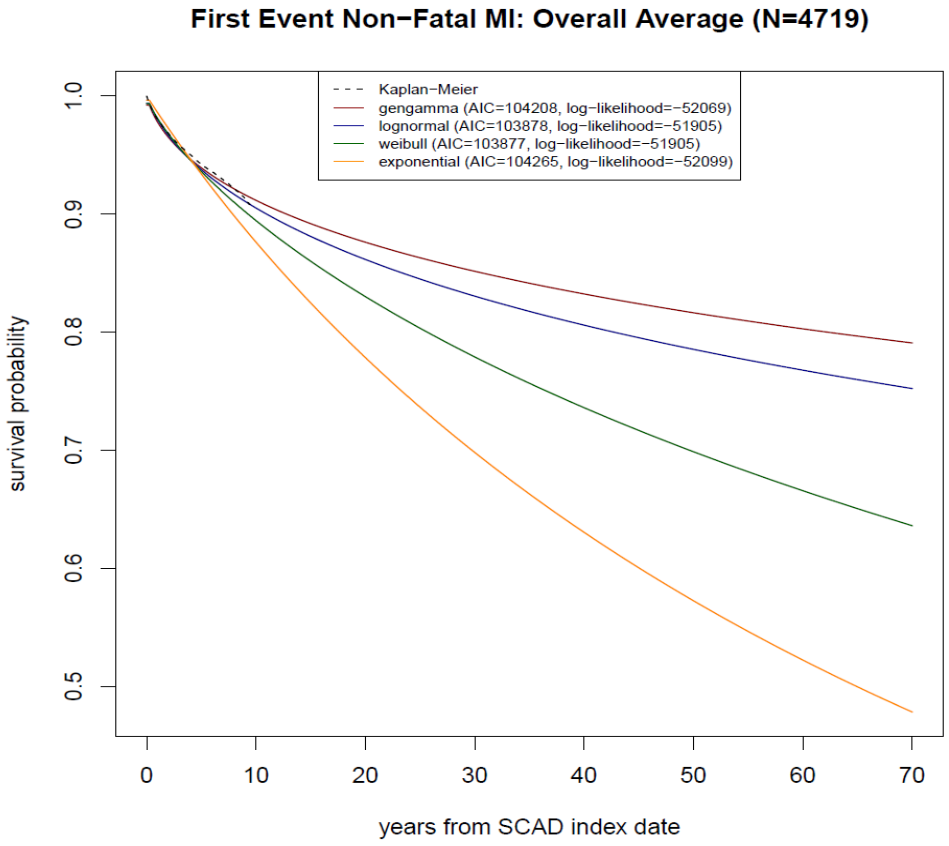
First Event Non−Fatal MI: Overall Average (N=4719)
Kaplan−Meier
gengamma (AIC=104208, log−likelihood=−52069)
lognormal (AIC=103878, log−likelihood=−51905)
weibull (AIC=103877, log−likelihood=−51905)
exponential (AIC=104265, log−likelihood=−52099)
survival probability
years from SCAD index date

Equation 2: FE Stroke I	GenGamma	LogNormal	Weibull	Exponential
**Sociodemographic characteristics**				
Age in men	0.95 (0.95-0.96)	0.95 (0.95-0.96)	0.96 (0.95-0.96)	0.96 (0.95-0.96)
Age in women	1.01 (1.00-1.02)	1.01 (1.00-1.01)	1.00 (1.00-1.01)	1.00 (1.00-1.01)
Women vs men	1.12 (0.98-1.28)	1.13 (1.02-1.25)	1.12 (1.02-1.23)	1.12 (1.02-1.23)
Most deprived quintile, yes vs. no	0.77 (0.68-0.87)	0.78 (0.71-0.86)	0.81 (0.74-0.88)	0.81 (0.75-0.88)
**SCAD diagnosis and severity**				
Other CHD vs. stable angina	0.99 (0.86-1.14)	1.01 (0.90-1.14)	1.00 (0.90-1.11)	1.00 (0.91-1.11)
NSTEMI vs. stable angina	1.00 (0.84-1.18)	0.92 (0.78-1.08)	0.93 (0.81-1.08)	0.93 (0.81-1.07)
STEMI vs stable angina	1.22 (0.68-2.18)	1.06 (0.84-1.34)	1.05 (0.84-1.31)	1.04 (0.84-1.30)
Unstable angina vs. stable angina	0.91 (0.75-1.11)	0.88 (0.77-0.99)	0.88 (0.79-0.98)	0.88 (0.79-0.98)
PCI in last 6 months	1.09 (0.80-1.49)	1.13 (0.94-1.37)	1.14 (0.95-1.36)	1.13 (0.95-1.35)
CABG in last 6 months	1.21 (0.96-1.52)	1.19 (0.95-1.48)	1.15 (0.94-1.41)	1.15 (0.95-1.40)
Previous/recurrent MI	0.87 (0.69-1.08)	0.88 (0.78-0.99)	0.90 (0.81-1.00)	0.90 (0.82-1.00)
Use of nitrates	0.97 (0.84-1.14)	0.96 (0.88-1.05)	0.97 (0.89-1.05)	0.97 (0.89-1.04)
**CVD risk factors**				
Current smoker vs. never	0.74 (0.59-0.92)	0.74 (0.65-0.84)	0.79 (0.71-0.88)	0.80 (0.72-0.89)
Ex-smoker vs. never	0.99 (0.81-1.21)	1.01 (0.89-1.14)	1.01 (0.90-1.14)	1.01 (0.91-1.13)
Hypertension	1.02 (0.85-1.22)	1.04 (0.93-1.15)	1.02 (0.93-1.13)	1.02 (0.93-1.12)
Diabetes mellitus	0.69 (0.60-0.80)	0.72 (0.64-0.80)	0.74 (0.67-0.82)	0.75 (0.68-0.82)
Total cholesterol, per 1 mmol/L increase	0.94 (0.86-1.02)	0.93 (0.89-0.99)	0.95 (0.90-1.00)	0.95 (0.91-1.00)
HDL, per 0.5 mmol/L increase	1.00 (0.90-1.11)	1.00 (0.91-1.10)	0.99 (0.91-1.07)	0.99 (0.91-1.07)
**CVD co-morbidities**				
Heart failure	0.85 (0.75-0.96)	0.86 (0.78-0.95)	0.90 (0.83-0.98)	0.90 (0.83-0.98)
Peripheral arterial disease	0.80 (0.67-0.96)	0.84 (0.73-0.96)	0.87 (0.78-0.98)	0.87 (0.78-0.98)
Atrial fibrillation	0.59 (0.51-0.68)	0.62 (0.56-0.69)	0.66 (0.60-0.72)	0.67 (0.61-0.73)
Stroke	0.23 (0.19-0.28)	0.22 (0.20-0.25)	0.30 (0.27-0.33)	0.31 (0.28-0.33)
**Non-CVD co-morbidities**				
Chornic kidney disease	1.23 (0.96-1.57)	1.11 (0.92-1.34)	1.05 (0.88-1.25)	1.03 (0.87-1.23)
Chronic obstructive pulmonary disease	1.07 (0.96-1.20)	1.08 (0.98-1.19)	1.07 (0.98-1.16)	1.06 (0.98-1.16)
Cancer	1.08 (0.89-1.31)	1.03 (0.90-1.19)	1.04 (0.92-1.17)	1.03 (0.92-1.17)
Chronic liver disease	0.72 (0.41-1.24)	0.79 (0.52-1.19)	0.78 (0.55-1.12)	0.79 (0.55-1.12)
**Psychosocial characteristics**				
Depression at diagnosis	0.90 (0.76-1.06)	0.90 (0.81-1.01)	0.89 (0.81-0.98)	0.89 (0.81-0.98)
Anxiety at diagnosis	0.96 (0.80-1.16)	0.94 (0.81-1.09)	0.94 (0.82-1.07)	0.94 (0.83-1.07)
**Biomarkers**				
Heart rate, per 10 b.p.m. increase	1.00 (0.93-1.07)	0.99 (0.94-1.04)	0.99 (0.95-1.03)	0.99 (0.95-1.03)
Creatinine, per 30 micromol/L increase	0.97 (0.90-1.03)	0.96 (0.90-1.02)	0.97 (0.92-1.02)	0.97 (0.92-1.02)
White cell count, per 1.5 10^9/L increase	0.92 (0.88-0.97)	0.93 (0.90-0.97)	0.94 (0.91-0.97)	0.94 (0.91-0.97)
Haemoglobin, per 1.5 g/dL increase	1.04 (0.99-1.09)	1.04 (0.98-1.09)	1.03 (0.99-1.08)	1.03 (0.99-1.08)
**Generalised gamma model parameters**				
mu	12.56 (12.32-12.8)	12.37 (12.17-12.57)	11.28 (11.11-11.45)	11.19 (11.07-11.31)
sigma	2.72 (2.29-3.23)	2.47 (2.40-2.54)	1.02 (0.99-1.06)	1
Q	-0.09 (-0.36-0.17)	0	1	1
**Model Fit**				
Log-likelihood	-36689.77	-36652.82	-36630.25	-36631.56
AIC	73449.54	73373.64	73328.51	73329.11

First Event Non−Fatal Ischaemic Stroke: Overall Average (N=3222)

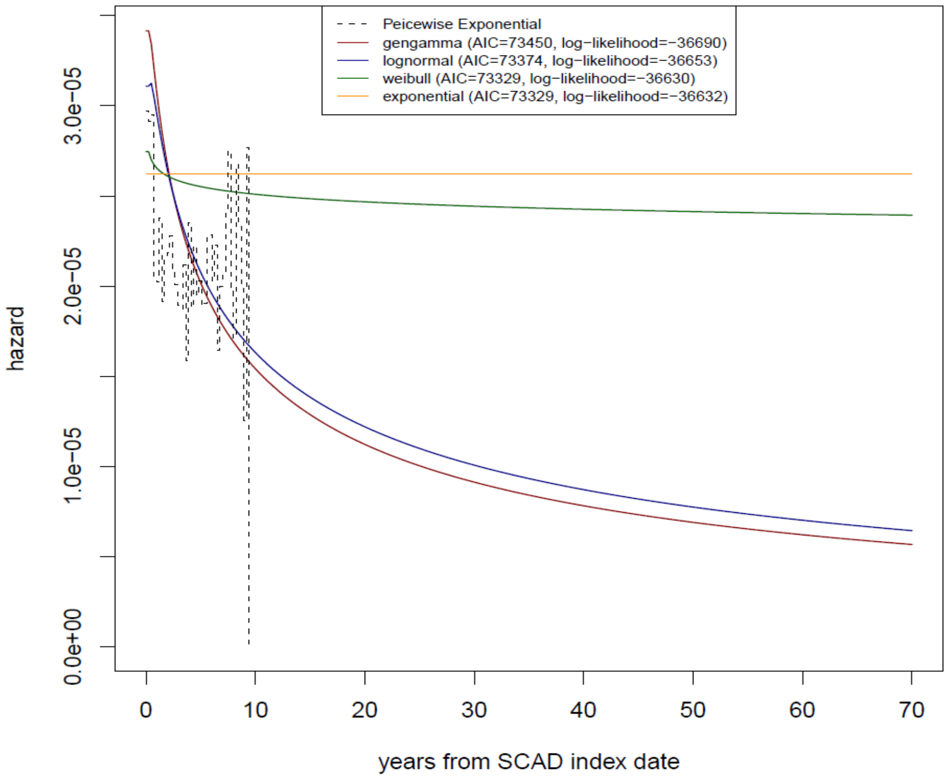

First Event Non−Fatal Ischaemic Stroke: Overall Average (N=3222)

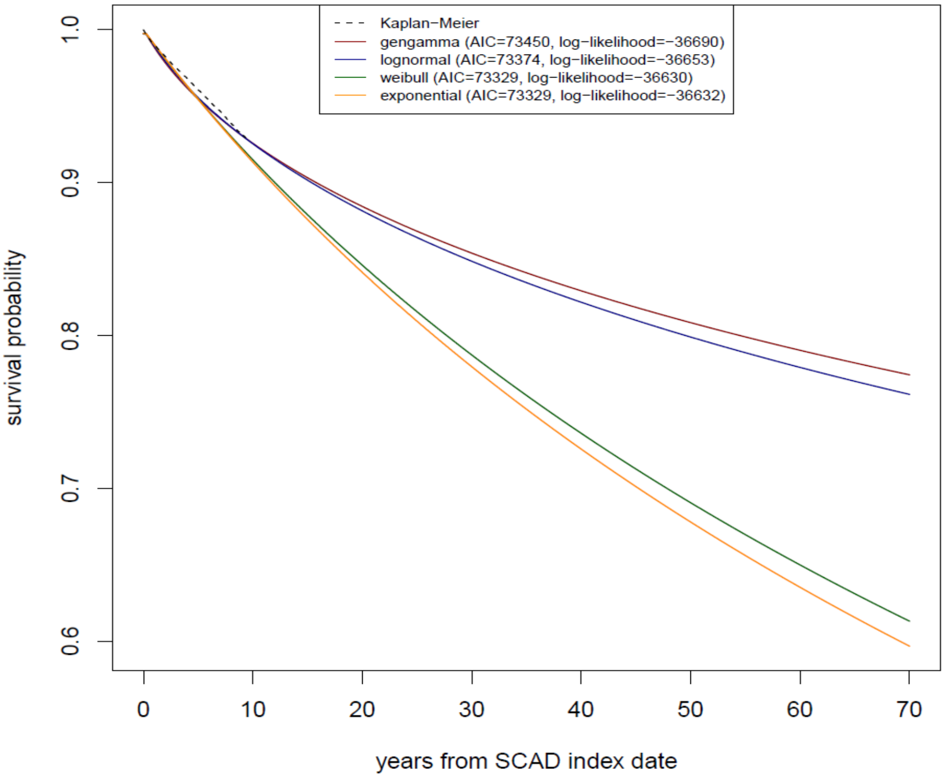

Equation 3: FE Stroke H	GenGamma	LogNormal	Weibull	Exponential
**Sociodemographic characteristics**				
Age in men	0.94 (0.92-0.96)	0.95 (0.93-0.97)	0.95 (0.94-0.97)	0.95 (0.94-0.97)
Age in women	1.02 (0.98-1.05)	1.01 (0.98-1.03)	1.01 (0.99-1.03)	1.01 (0.99-1.03)
Women vs men	1.83 (1.24-2.70)	1.49 (1.11-2.00)	1.41 (1.07-1.86)	1.39 (1.07-1.81)
Most deprived quintile, yes vs. no				
**SCAD diagnosis and severity**				
Other CHD vs. stable angina				
NSTEMI vs. stable angina				
STEMI vs stable angina				
Unstable angina vs. stable angina				
PCI in last 6 months				
CABG in last 6 months				
Previous/recurrent MI				
Use of nitrates				
**CVD risk factors**				
Current smoker vs. never				
Ex-smoker vs. never				
Hypertension				
Diabetes mellitus				
Total cholesterol, per 1 mmol/L increase				
HDL, per 0.5 mmol/L increase				
**CVD co-morbidities**				
Heart failure				
Peripheral arterial disease				
Atrial fibrillation				
Stroke				
**Non-CVD co-morbidities**				
Chornic kidney disease				
Chronic obstructive pulmonary disease				
Cancer				
Chronic liver disease				
**Psychosocial characteristics**				
Depression at diagnosis				
Anxiety at diagnosis				
**Biomarkers**				
Heart rate, per 10 b.p.m. increase				
Creatinine, per 30 micromol/L increase				
White cell count, per 1.5 10^9/L increase				
Haemoglobin, per 1.5 g/dL increase				
**Generalised gamma model parameters**				
mu	23.07 (21.60-24.54)	16.59 (15.65-17.53)	13.36 (12.73-13.99)	13.09 (12.93-13.25)
sigma	14.52 (12.56-16.78)	3.41 (3.09-3.77)	1.05 (0.94-1.17)	1
Q	-2.76 (-3.32--2.19)	0	1	1
**Model Fit**				
Log-likelihood	-3711.20	-3694.11	-3691.75	-3692.16
AIC	7434.41	7398.23	7393.50	7392.32

First Event Non−Fatal Hemorrhagic Stroke: Overall Average (N=262)

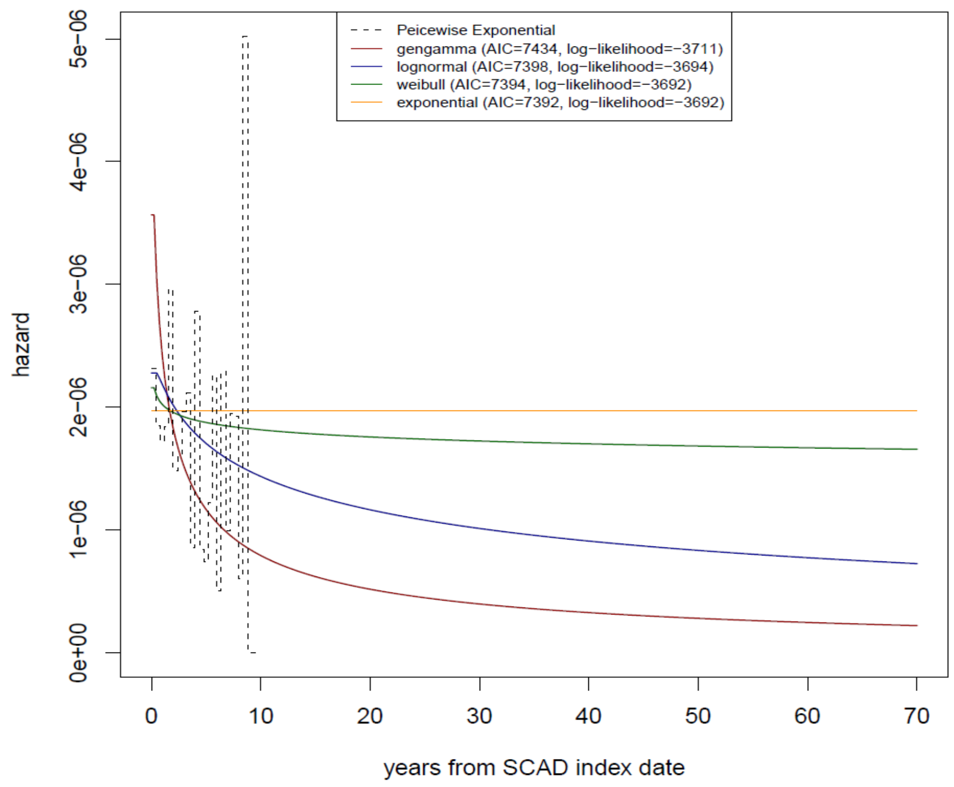

First Event Non−Fatal Hemorrhagic Stroke: Overall Average (N=262)

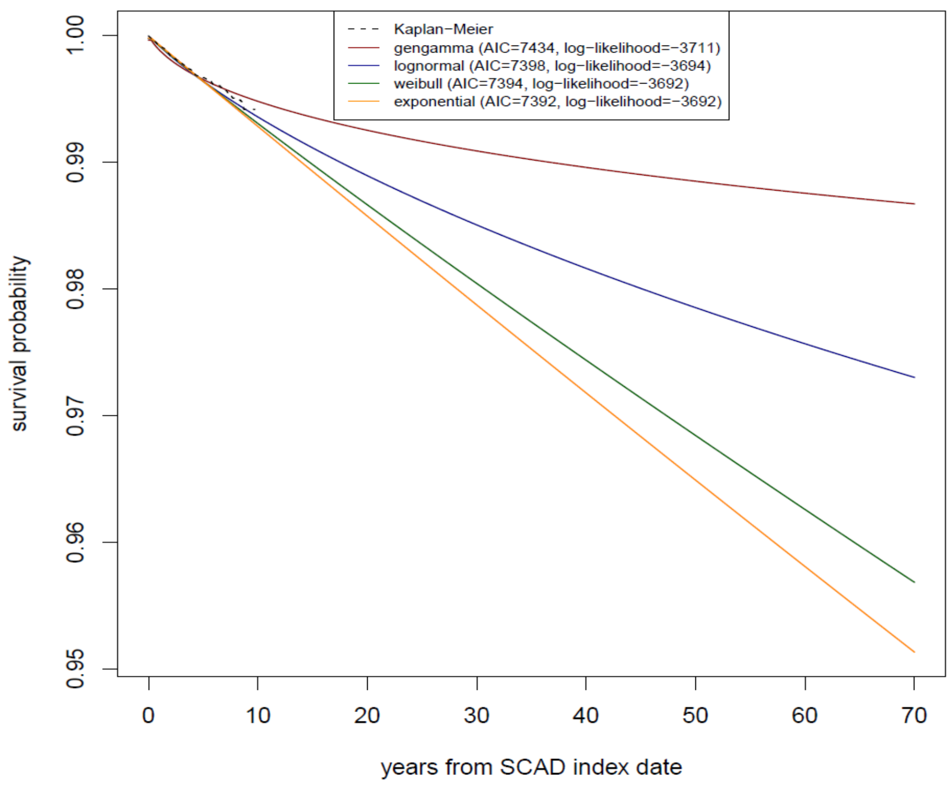

Equation 4: FE Fatal CVD	GenGamma	LogNormal	Weibull	Exponential
**Sociodemographic characteristics**				
Age in men	0.94 (0.92-0.96)	0.94 (0.93-0.94)	0.94 (0.94-0.94)	0.94 (0.94-0.94)
Age in women	0.97 (0.95-1.00)	0.97 (0.97-0.98)	0.97 (0.97-0.98)	0.97 (0.97-0.98)
Women vs men	1.82 (0.95-3.46)	2.04 (1.86-2.24)	1.97 (1.81-2.16)	2.00 (1.83-2.19)
Most deprived quintile, yes vs. no	0.90 (0.51-1.58)	0.85 (0.79-0.92)	0.90 (0.84-0.96)	0.90 (0.84-0.96)
**SCAD diagnosis and severity**				
Other CHD vs. stable angina	0.84 (0.57-1.24)	0.85 (0.78-0.93)	0.85 (0.79-0.92)	0.85 (0.78-0.91)
NSTEMI vs. stable angina	0.54 (0.33-0.88)	0.54 (0.48-0.60)	0.57 (0.53-0.63)	0.57 (0.52-0.62)
STEMI vs stable angina	0.73 (0.29-1.84)	0.74 (0.62-0.87)	0.77 (0.65-0.90)	0.77 (0.65-0.90)
Unstable angina vs. stable angina	0.91 (0.61-1.34)	0.90 (0.82-1.00)	0.89 (0.81-0.97)	0.89 (0.81-0.97)
PCI in last 6 months	1.42 (0.56-3.56)	1.71 (1.46-2.00)	1.82 (1.55-2.13)	1.85 (1.58-2.18)
CABG in last 6 months	1.58 (0.46-5.46)	2.09 (1.73-2.51)	1.98 (1.65-2.36)	2.00 (1.67-2.41)
Previous/recurrent MI	0.68 (0.52-0.90)	0.72 (0.66-0.78)	0.76 (0.72-0.82)	0.76 (0.71-0.81)
Use of nitrates	0.71 (0.59-0.85)	0.70 (0.65-0.74)	0.75 (0.71-0.79)	0.74 (0.70-0.79)
**CVD risk factors**				
Current smoker vs. never	0.60 (0.34-1.04)	0.76 (0.68-0.84)	0.80 (0.73-0.87)	0.79 (0.72-0.86)
Ex-smoker vs. never	0.77 (0.51-1.17)	0.95 (0.86-1.05)	0.96 (0.87-1.05)	0.96 (0.87-1.05)
Hypertension	0.93 (0.71-1.22)	0.98 (0.90-1.06)	0.98 (0.91-1.06)	0.98 (0.91-1.06)
Diabetes mellitus	0.79 (0.57-1.11)	0.73 (0.68-0.80)	0.75 (0.70-0.80)	0.75 (0.70-0.80)
Total cholesterol, per 1 mmol/L increase	0.97 (0.84-1.13)	0.96 (0.91-1.00)	0.97 (0.93-1.01)	0.97 (0.92-1.01)
HDL, per 0.5 mmol/L increase	1.05 (0.81-1.36)	1.05 (0.99-1.12)	1.03 (0.98-1.08)	1.03 (0.98-1.09)
**CVD co-morbidities**				
Heart failure	0.47 (0.38-0.59)	0.52 (0.48-0.55)	0.58 (0.54-0.61)	0.57 (0.53-0.60)
Peripheral arterial disease	0.70 (0.44-1.12)	0.72 (0.66-0.79)	0.75 (0.70-0.81)	0.75 (0.69-0.81)
Atrial fibrillation	0.69 (0.59-0.82)	0.73 (0.68-0.79)	0.76 (0.72-0.81)	0.76 (0.71-0.81)
Stroke	0.63 (0.35-1.14)	0.66 (0.60-0.71)	0.72 (0.67-0.77)	0.71 (0.66-0.77)
**Non-CVD co-morbidities**				
Chornic kidney disease	1.04 (0.33-3.33)	0.98 (0.86-1.11)	0.94 (0.84-1.04)	0.95 (0.85-1.05)
Chronic obstructive pulmonary disease	1.22 (0.78-1.91)	1.05 (0.98-1.13)	1.03 (0.97-1.10)	1.04 (0.97-1.10)
Cancer	1.29 (0.83-2.00)	1.10 (1.00-1.22)	1.12 (1.03-1.22)	1.12 (1.03-1.23)
Chronic liver disease	0.44 (0.09-2.09)	0.64 (0.48-0.85)	0.76 (0.59-0.99)	0.76 (0.59-0.99)
**Psychosocial characteristics**				
Depression at diagnosis	0.89 (0.58-1.37)	0.90 (0.83-0.98)	0.89 (0.83-0.96)	0.89 (0.83-0.96)
Anxiety at diagnosis	0.71 (0.39-1.28)	0.85 (0.77-0.95)	0.88 (0.80-0.97)	0.88 (0.80-0.97)
**Biomarkers**				
Heart rate, per 10 b.p.m. increase	0.89 (0.81-0.98)	0.90 (0.87-0.93)	0.92 (0.89-0.95)	0.92 (0.89-0.94)
Creatinine, per 30 micromol/L increase	0.89 (0.81-0.97)	0.89 (0.86-0.92)	0.91 (0.89-0.93)	0.90 (0.88-0.93)
White cell count, per 1.5 10^9/L increase	0.85 (0.77-0.95)	0.89 (0.86-0.93)	0.91 (0.88-0.94)	0.91 (0.88-0.94)
Haemoglobin, per 1.5 g/dL increase	1.30 (1.12-1.50)	1.28 (1.23-1.32)	1.23 (1.19-1.26)	1.23 (1.19-1.27)
**Generalised gamma model parameters**				
mu	12.33 (11.37-13.29)	11.49 (11.35-11.63)	10.9 (10.77-11.02)	10.98 (10.88-11.09)
sigma	4.24 (3.86-4.66)	2.09 (2.05-2.14)	0.97 (0.95-1.00)	1
Q	-1.30 (-1.78--0.81)	0	1	1
**Model Fit**				
Log-likelihood	-58770.27	-57762.41	-57592.89	-57595.48
AIC	117610.54	115592.82	115253.77	115256.97

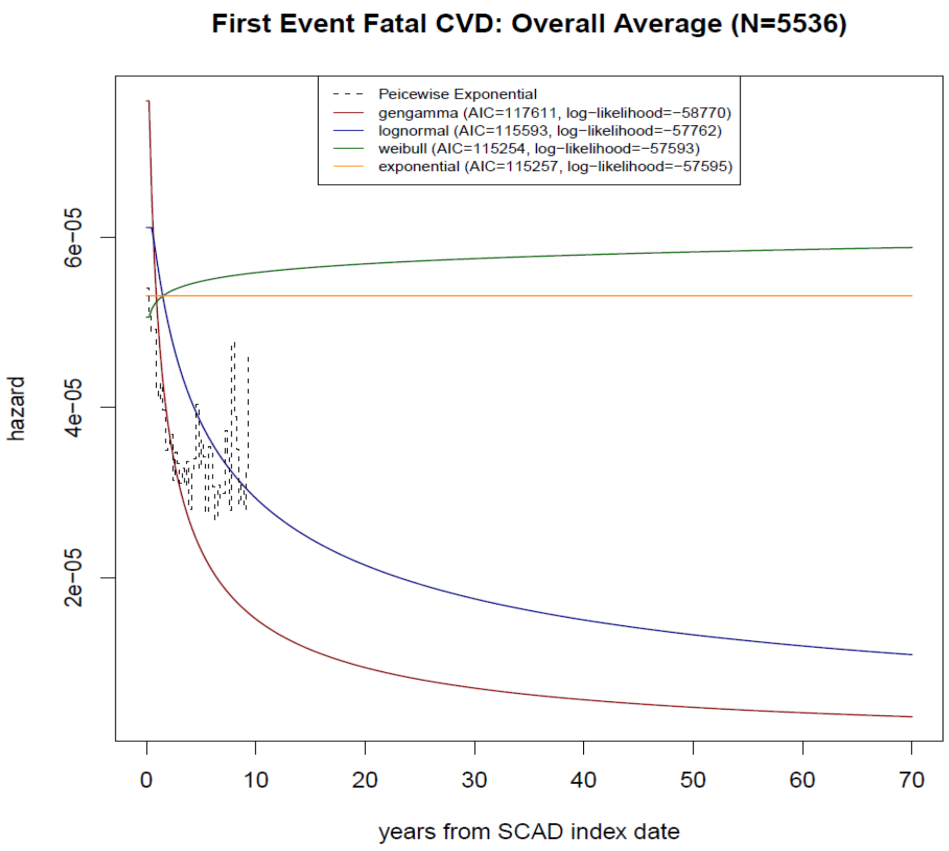
First Event Fatal CVD: Overall Average (N=5536)
Peicewise Exponential
gengamma (AIC=117611, log−likelihood=−58770)
lognormal (AIC=115593, log−likelihood=−57762)
weibull (AIC=115254, log−likelihood=−57593)
exponential (AIC=115257, log−likelihood=−57595)
hazard
2e−05
4e−05
6e−05
0
10
20
30
40
50
60
70
years from SCAD index date

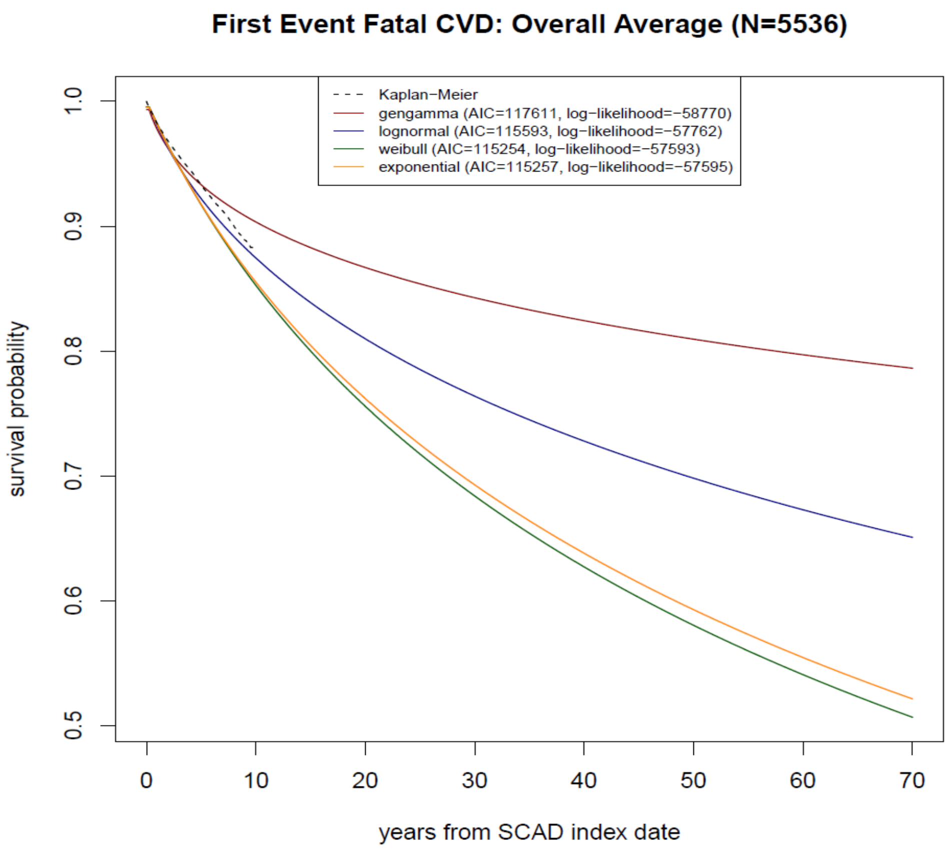
First Event Fatal CVD: Overall Average (N=5536)
Kaplan−Meier
gengamma (AIC=117611, log−likelihood=−58770)
lognormal (AIC=115593, log−likelihood=−57762)
weibull (AIC=115254, log−likelihood=−57593)
exponential (AIC=115257, log−likelihood=−57595)
survival probability
0.5
0.6
0.7
0.8
0.9
1.0
0
10
20
30
40
50
60
70
years from SCAD index date

Equation 5: FE Fatal non-CVD	GenGamma	LogNormal	Weibull	Exponential
**Sociodemographic characteristics**				
Age in men	0.94 (0.94-0.95)	0.94 (0.94-0.95)	0.94 (0.94-0.95)	0.94 (0.93-0.94)
Age in women	0.99 (0.98-0.99)	0.99 (0.98-0.99)	0.99 (0.98-0.99)	0.99 (0.98-0.99)
Women vs men	1.71 (1.60-1.84)	1.75 (1.65-1.86)	1.65 (1.56-1.74)	1.75 (1.64-1.87)
Most deprived quintile, yes vs. no	0.90 (0.74-1.08)	0.84 (0.79-0.88)	0.86 (0.82-0.90)	0.85 (0.80-0.89)
**SCAD diagnosis and severity**				
Other CHD vs. stable angina	1.03 (0.90-1.18)	1.04 (0.98-1.11)	1.02 (0.97-1.08)	1.02 (0.96-1.08)
NSTEMI vs. stable angina	0.91 (0.70-1.19)	0.89 (0.82-0.96)	0.90 (0.84-0.96)	0.90 (0.84-0.98)
STEMI vs stable angina	1.05 (0.62-1.77)	0.93 (0.83-1.04)	0.90 (0.81-1.00)	0.91 (0.80-1.02)
Unstable angina vs. stable angina	1.07 (0.91-1.26)	1.03 (0.96-1.10)	1.02 (0.96-1.09)	1.02 (0.95-1.10)
PCI in last 6 months	1.31 (1.07-1.59)	1.34 (1.21-1.50)	1.32 (1.19-1.47)	1.40 (1.24-1.58)
CABG in last 6 months	1.75 (1.23-2.50)	1.85 (1.62-2.12)	1.74 (1.53-1.99)	1.86 (1.60-2.17)
Previous/recurrent MI	1.02 (0.90-1.16)	1.01 (0.95-1.08)	1.00 (0.95-1.06)	1.00 (0.94-1.07)
Use of nitrates	0.90 (0.85-0.96)	0.89 (0.85-0.93)	0.92 (0.88-0.96)	0.91 (0.87-0.95)
**CVD risk factors**				
Current smoker vs. never	0.73 (0.65-0.81)	0.70 (0.65-0.76)	0.74 (0.69-0.80)	0.69 (0.64-0.75)
Ex-smoker vs. never	0.86 (0.79-0.94)	0.84 (0.78-0.91)	0.85 (0.80-0.91)	0.83 (0.77-0.89)
Hypertension	1.15 (1.08-1.22)	1.11 (1.05-1.18)	1.11 (1.06-1.17)	1.13 (1.07-1.19)
Diabetes mellitus	0.90 (0.81-1.00)	0.91 (0.86-0.97)	0.91 (0.86-0.95)	0.90 (0.85-0.95)
Total cholesterol, per 1 mmol/L increase	1.00 (0.96-1.04)	1.01 (0.98-1.04)	1.01 (0.98-1.04)	1.00 (0.97-1.04)
HDL, per 0.5 mmol/L increase	0.97 (0.94-1.00)	0.98 (0.95-1.01)	0.98 (0.95-1.01)	0.98 (0.95-1.01)
**CVD co-morbidities**				
Heart failure	0.72 (0.68-0.77)	0.71 (0.67-0.74)	0.76 (0.73-0.80)	0.74 (0.70-0.77)
Peripheral arterial disease	0.84 (0.76-0.93)	0.81 (0.75-0.86)	0.83 (0.78-0.87)	0.81 (0.76-0.87)
Atrial fibrillation	0.88 (0.75-1.03)	0.84 (0.80-0.90)	0.88 (0.84-0.93)	0.88 (0.83-0.93)
Stroke	0.86 (0.75-0.98)	0.84 (0.79-0.90)	0.87 (0.82-0.92)	0.86 (0.81-0.92)
**Non-CVD co-morbidities**				
Chornic kidney disease	0.92 (0.81-1.05)	0.91 (0.83-0.99)	0.89 (0.82-0.96)	0.93 (0.85-1.02)
Chronic obstructive pulmonary disease	0.74 (0.63-0.86)	0.73 (0.70-0.77)	0.76 (0.73-0.79)	0.74 (0.70-0.77)
Cancer	0.49 (0.42-0.57)	0.41 (0.39-0.44)	0.56 (0.53-0.58)	0.51 (0.49-0.54)
Chronic liver disease	0.44 (0.27-0.72)	0.43 (0.36-0.52)	0.53 (0.46-0.62)	0.50 (0.42-0.59)
**Psychosocial characteristics**				
Depression at diagnosis	0.81 (0.73-0.89)	0.80 (0.75-0.84)	0.82 (0.78-0.86)	0.80 (0.76-0.85)
Anxiety at diagnosis	0.83 (0.55-1.25)	0.78 (0.72-0.84)	0.83 (0.78-0.89)	0.82 (0.76-0.88)
**Biomarkers**				
Heart rate, per 10 b.p.m. increase	0.90 (0.86-0.95)	0.89 (0.87-0.92)	0.91 (0.89-0.93)	0.90 (0.88-0.93)
Creatinine, per 30 micromol/L increase	0.98 (0.93-1.04)	1.00 (0.98-1.02)	0.99 (0.97-1.01)	0.99 (0.97-1.01)
White cell count, per 1.5 10^9/L increase	0.87 (0.82-0.92)	0.85 (0.84-0.87)	0.89 (0.87-0.90)	0.87 (0.86-0.89)
Haemoglobin, per 1.5 g/dL increase	1.38 (1.33-1.42)	1.41 (1.37-1.44)	1.33 (1.30-1.36)	1.38 (1.35-1.41)
**Generalised gamma model parameters**				
mu	10.1 (10.01-10.19)	10.25 (10.15-10.34)	9.95 (9.87-10.03)	10.32 (10.24-10.40)
sigma	1.37 (1.11-1.70)	1.73 (1.7-1.76)	0.86 (0.85-0.88)	1
Q	0.46 (0.19-0.73)	0	1	1
**Model Fit**				
Log-likelihood	-87260.46	-87397.88	-87058.62	-87182.58
AIC	174590.93	174863.76	174185.23	174431.16

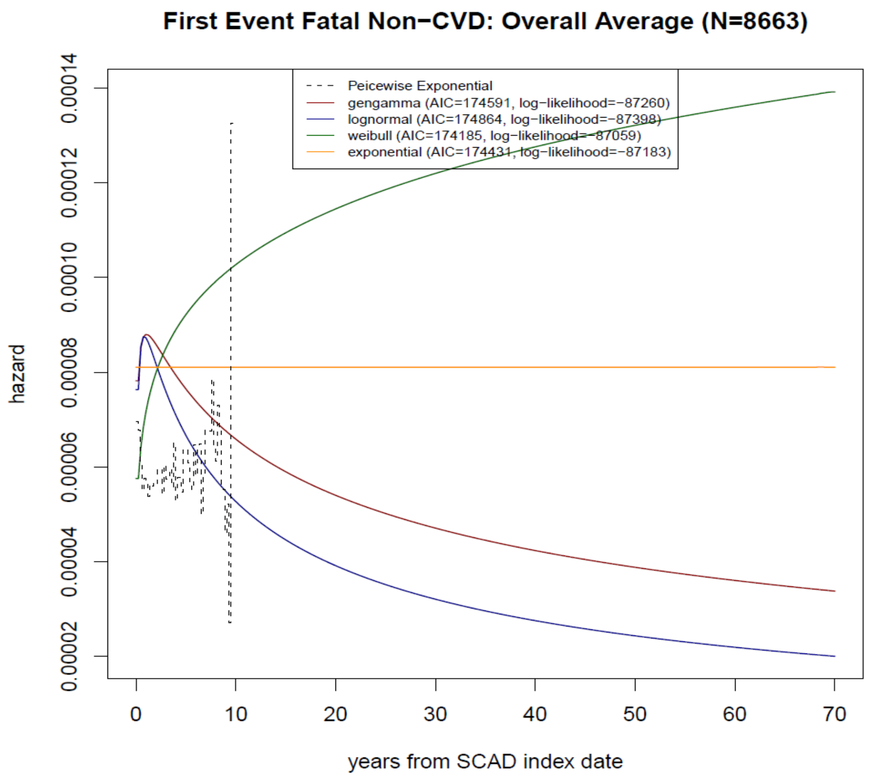
First Event Fatal Non−CVD: Overall Average (N=8663)
Peicewise Exponential
gengamma (AIC=174591, log−likelihood=−87260)
lognormal (AIC=174864, log−likelihood=−87398)
weibull (AIC=174185, log−likelihood=−87059)
exponential (AIC=174431, log−likelihood=−87183)
hazard
0.00002
0.00004
0.00006
0.00008
0.00010
0.00012
0.00014
0
10
20
30
40
50
60
70
years from SCAD index date

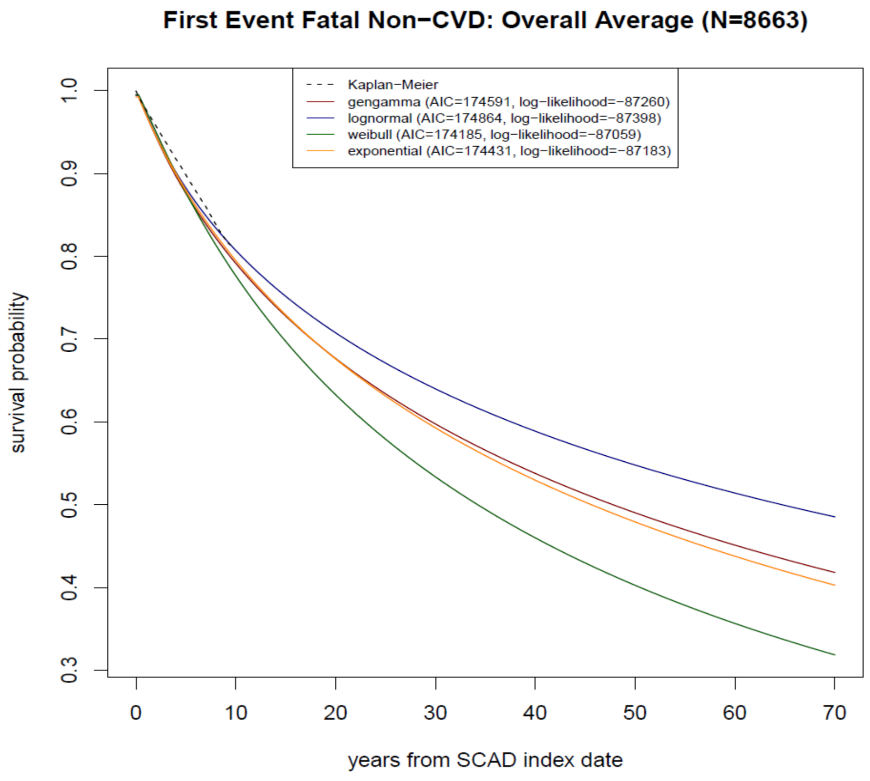
First Event Fatal Non−CVD: Overall Average (N=8663)
Kaplan−Meier
gengamma (AIC=174591, log−likelihood=−87260)
lognormal (AIC=174864, log−likelihood=−87398)
weibull (AIC=174185, log−likelihood=−87059)
exponential (AIC=174431, log−likelihood=−87183)
survival probability
0.3
0.4
0.5
0.6
0.7
0.8
0.9
1.0
0
10
20
30
40
50
60
70
years from SCAD index date

Equation 6: Post MI Fatal CVD	GenGamma	LogNormal	Weibull	Exponential
**Sociodemographic characteristics**				
Age in men	0.85 (0.83-0.87)	0.85 (0.83-0.87)	0.85 (0.83-0.87)	0.92 (0.91-0.93)
Age in women	0.98 (0.95-1.02)	0.98 (0.95-1.02)	0.98 (0.95-1.02)	0.99 (0.97-1.00)
Women vs men	1.87 (1.12-3.11)	1.87 (1.12-3.11)	1.90 (1.12-3.22)	1.39 (1.11-1.74)
**Generalised gamma model parameters**				
mu	11.14 (10.71-11.56)	11.13 (10.74-11.51)	11.27 (10.89-11.65)	8.85 (8.74-8.96)
sigma	4.21 (3.60-4.93)	4.24 (4.02-4.48)	2.39 (2.25-2.54)	1
Q	0.02 (-0.32-0.36)	0	1	1
**Model Fit**				
Log-likelihood	-6781.95	-6781.95	-6798.12	-7388.97
AIC	13575.89	13573.90	13606.23	14785.94

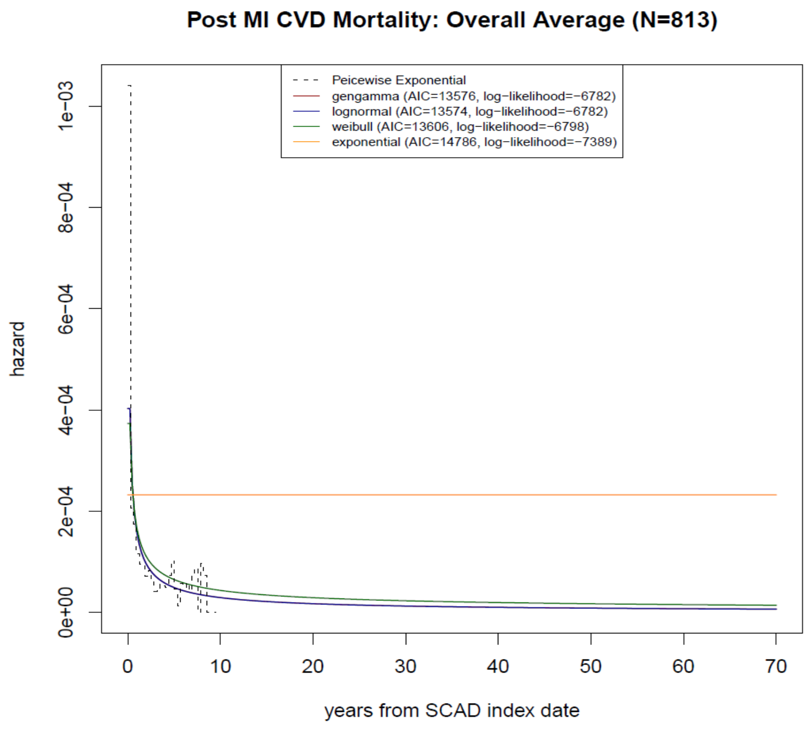

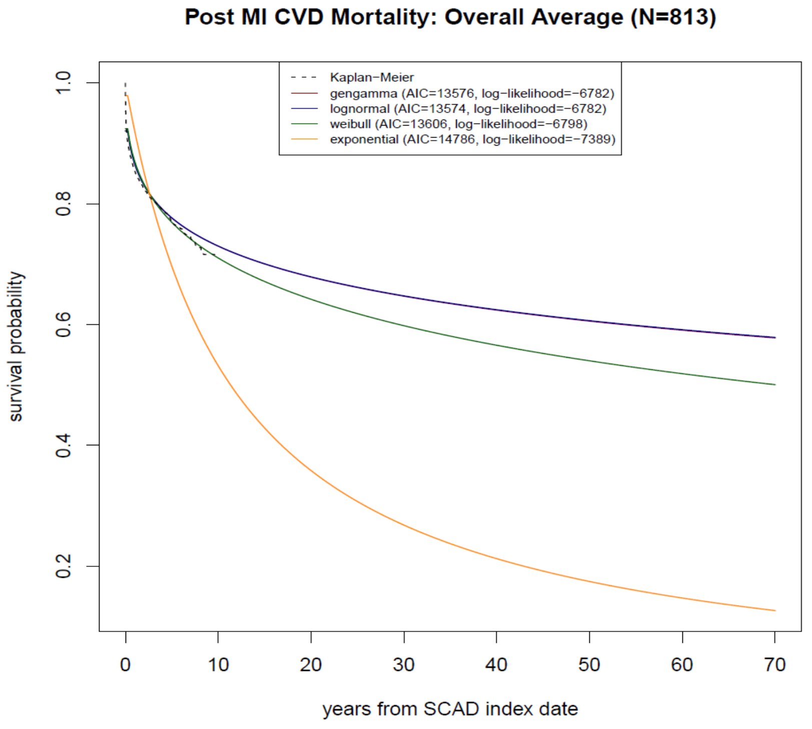

Equation 7: Post MI Fatal Non-CVD	GenGamma	LogNormal	Weibull	Exponential
**Sociodemographic characteristics**				
Age in men	0.88 (0.86-0.89)	0.87 (0.85-0.88)	0.87 (0.86-0.89)	0.91 (0.90-0.92)
Age in women	1.03 (1.00-1.05)	1.02 (0.99-1.05)	1.03 (1.00-1.05)	1.01 (1.00-1.03)
Women vs men	0.91 (0.64-1.30)	1.04 (0.71-1.52)	0.95 (0.66-1.37)	1.00 (0.81-1.24)
**Generalised gamma model parameters**				
mu	10.38 (10.02-10.75)	10.38 (10.07-10.69)	10.32 (10.03-10.6)	8.99 (8.87-9.11)
sigma	0.77 (0.37-1.61)	3.27 (3.09-3.45)	1.73 (1.63-1.84)	1
Q	2.47 (0.63-4.3)	0	1	1
**Model Fit**				
Log-likelihood	-6755.79	-6788.29	-6762.82	-6972.03
AIC	13523.58	13586.59	13535.64	13952.06

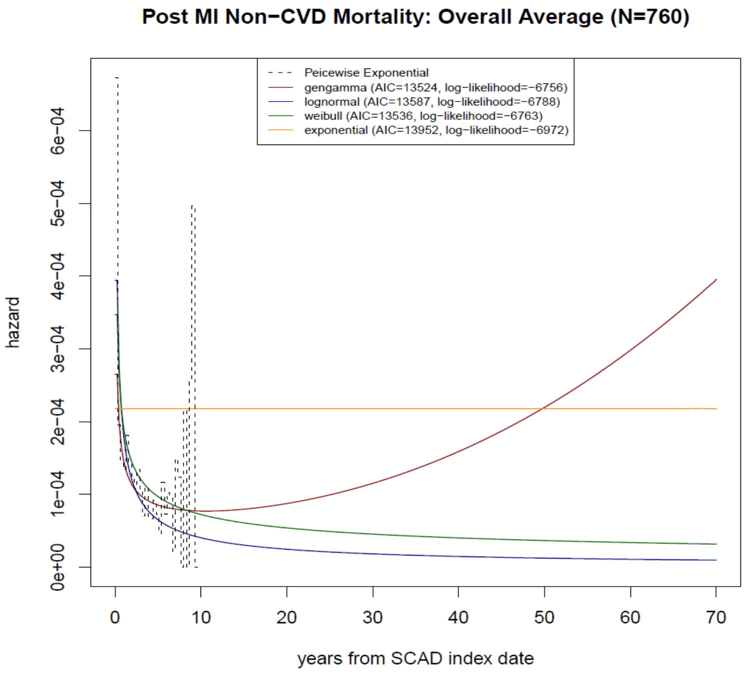

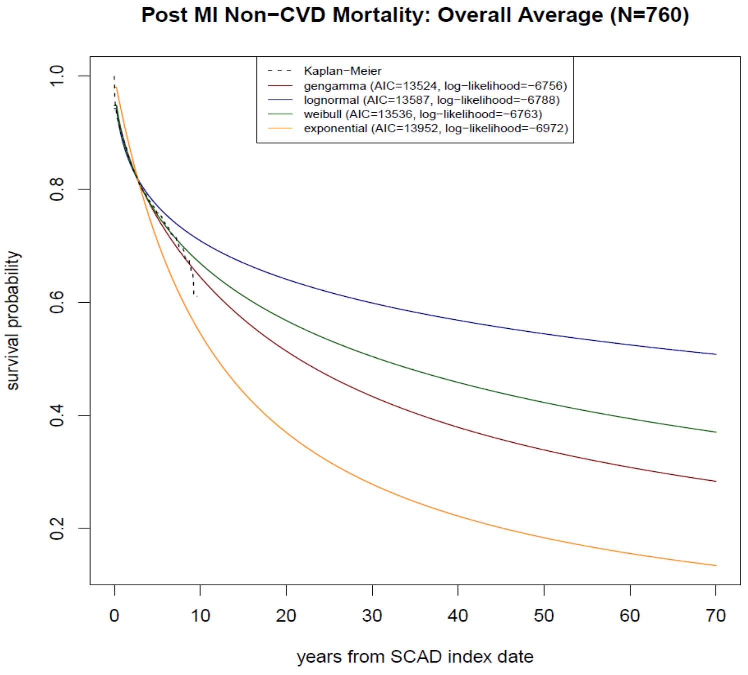

Equation 8: Post Ischaemic Stroke Fatal CVD	GenGamma	LogNormal	Weibull	Exponential
**Sociodemographic characteristics**				
Age in men	0.91 (0.89-0.93)	0.91 (0.89-0.94)	0.91 (0.89-0.93)	0.94 (0.92-0.95)
Age in women	0.99 (0.96-1.03)	0.99 (0.95-1.03)	0.99 (0.96-1.03)	0.99 (0.97-1.01)
Women vs men	1.52 (0.90-2.54)	1.54 (0.90-2.62)	1.54 (0.91-2.59)	1.35 (0.98-1.86)
**Generalised gamma model parameters**				
mu	10.42 (9.45-11.39)	10.68 (10.22-11.14)	10.4 (9.98-10.81)	9.08 (8.89-9.27)
sigma	0.59 (0.04-9.78)	3.30 (3.07-3.56)	1.67 (1.54-1.81)	1
Q	3.00 (-5.42-11.42)	0	1	1
**Model Fit**				
Log-likelihood	-3786.80	-3796.47	-3789.03	-3883.70
AIC	7585.61	7602.95	7588.07	7775.40

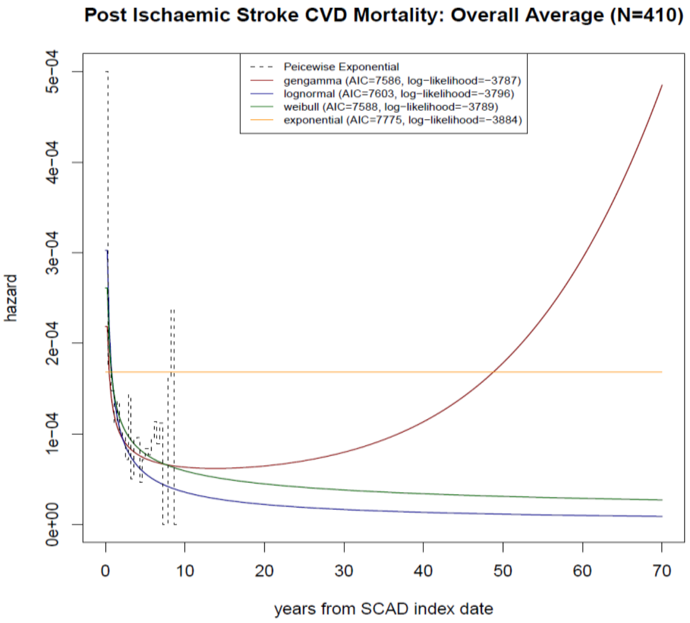

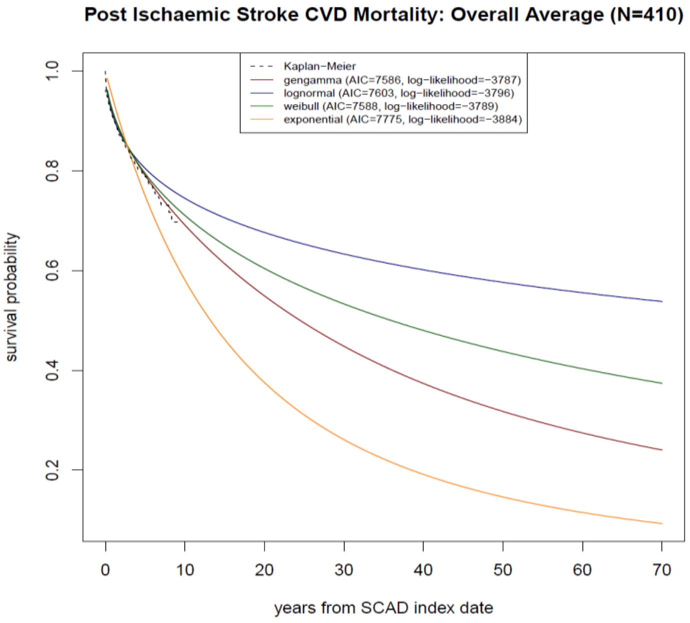

Equation 9: Post Ischaemic Stroke Fatal Non-CVD	GenGamma	LogNormal	Weibull	Exponential
**Sociodemographic characteristics**				
Age in men	0.93 (0.91-0.95)	0.93 (0.91-0.95)	0.93 (0.91-0.95)	0.95 (0.94-0.96)
Age in women	0.99 (0.97-1.03)	1.01 (0.97-1.04)	1.00 (0.97-1.03)	1.00 (0.98-1.02)
Women vs men	1.48 (0.97-2.26)	1.59 (1.02-2.49)	1.50 (0.97-2.31)	1.32 (1.02-1.71)
**Generalised gamma model parameters**				
mu	9.92 (8.53-11.3)	9.86 (9.49-10.23)	9.80 (9.47-10.13)	8.70 (8.54-8.85)
sigma	0.53 (0.01-38.25)	3.23 (3.02-3.45)	1.69 (1.57-1.82)	1
Q	3.40 (-11.15-17.96)	0	1	1
**Model Fit**				
Log-likelihood	-4747.78	-4760.80	-4751.72	-4879.00
AIC	9507.57	9531.60	9513.44	9765.99

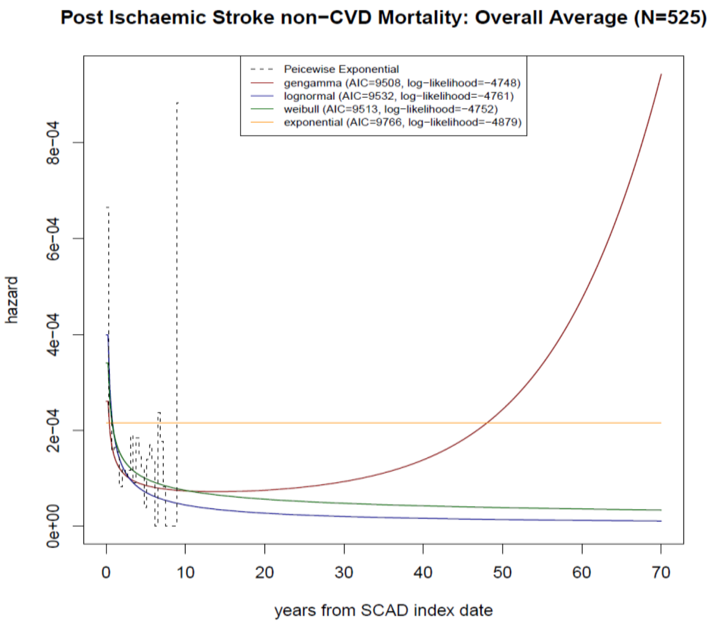

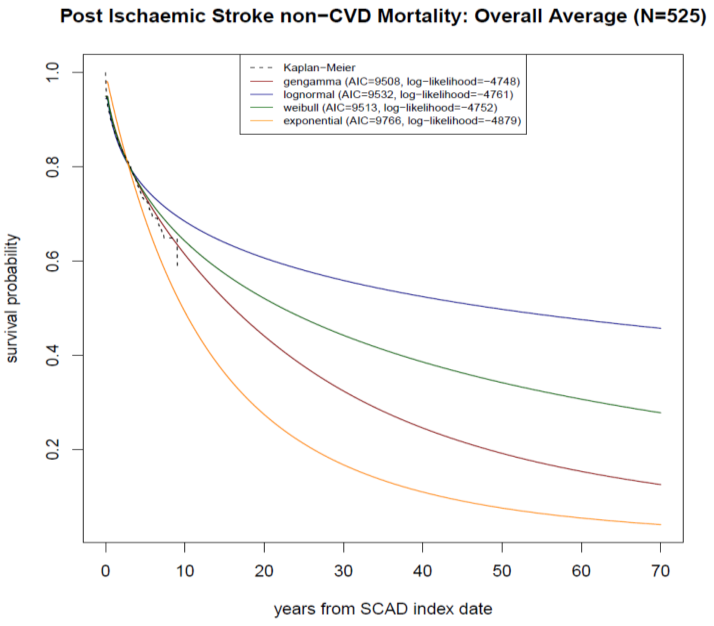

Equation 10: Post Hemorrhagic Stroke Fatal CVD	GenGamma	LogNormal	Weibull	Exponential
**Sociodemographic characteristics**				
Age in men	0.88 (0.80-0.96)	0.88 (0.80-0.96)	0.89 (0.81-0.97)	0.94 (0.90-0.97)
Age in women	1.02 (0.87-1.20)	1.02 (0.87-1.19)	1.04 (0.90-1.19)	1.02 (0.96-1.08)
Women vs men	0.79 (0.09-6.85)	0.85 (0.11-6.58)	1.06 (0.15-7.27)	1.19 (0.52-2.76)
**Generalised gamma model parameters**				
mu	10.95 (8.99-12.9)	11.02 (9.31-12.74)	10.79 (9.18-12.41)	8.58 (8.06-9.09)
sigma	4.60 (2.04-10.36)	4.14 (3.27-5.25)	2.25 (1.73-2.92)	1
Q	-0.26 (-2.4-1.89)	0	1	1
**Model Fit**				
Log-likelihood	-346.21	-346.24	-346.99	-373.82
AIC	704.42	702.48	703.98	755.64

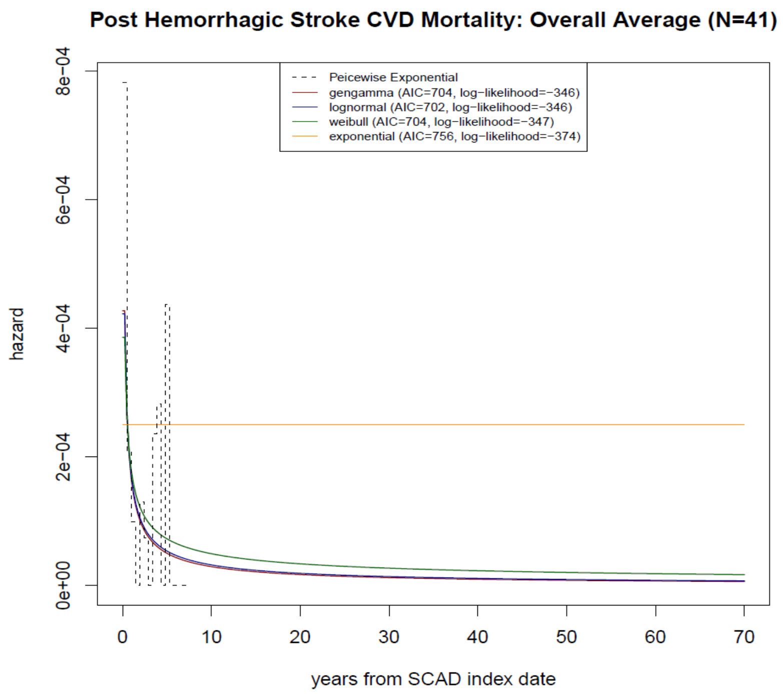

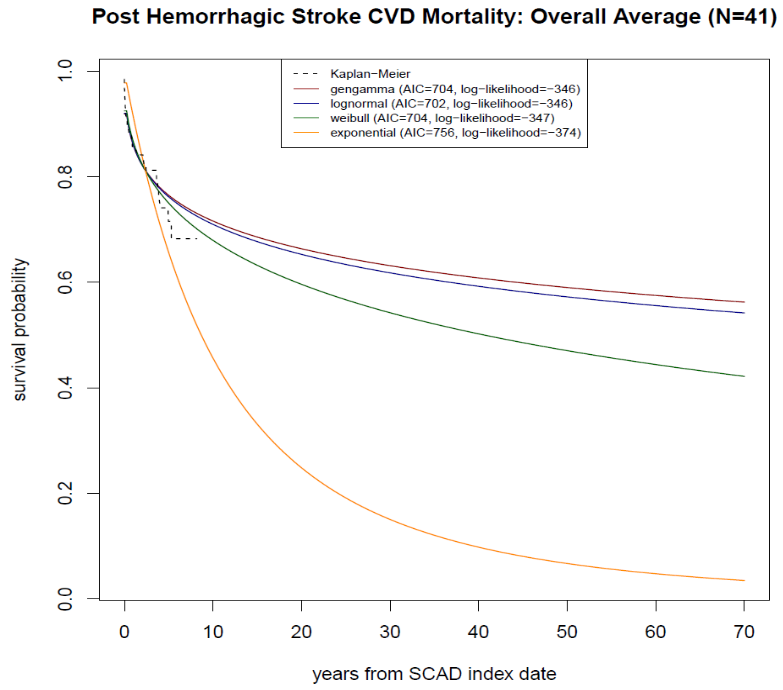

Equation 11: Post Hemorrhagic Stroke Fatal Non-CVD	GenGamma	LogNormal	Weibull	Exponential
**Sociodemographic characteristics**				
Age in men	0.91 (0.84-0.99)	0.91 (0.84-0.99)	0.92 (0.85-0.99)	0.95 (0.91-0.99)
Age in women	0.95 (0.81-1.12)	0.95 (0.81-1.11)	0.94 (0.8-1.09)	0.97 (0.91-1.04)
Women vs men	5.81 (0.68-49.96)	5.64 (0.62-51.43)	5.70 (0.57-57.48)	2.59 (0.88-7.65)
**Generalised gamma model parameters**				
mu	9.86 (7.32-12.39)	10.24 (8.70-11.79)	10.22 (8.81-11.63)	8.51 (8.03-9.00)
sigma	4.55 (2.38-8.72)	3.68 (2.85-4.75)	2.01 (1.53-2.66)	1
Q	-0.61 (-2.86-1.64)	0	1	1
**Model Fit**				
Log-likelihood	-305.31	-305.49	-306.60	-323.30
AIC	622.63	620.97	623.20	654.61

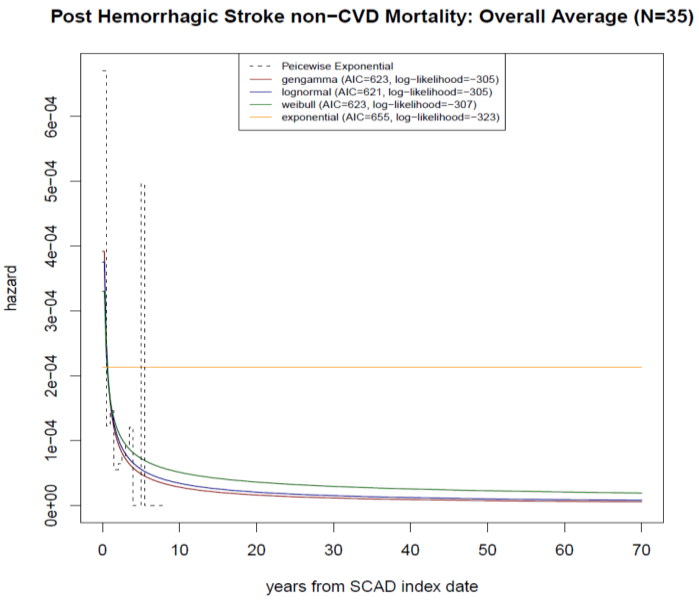

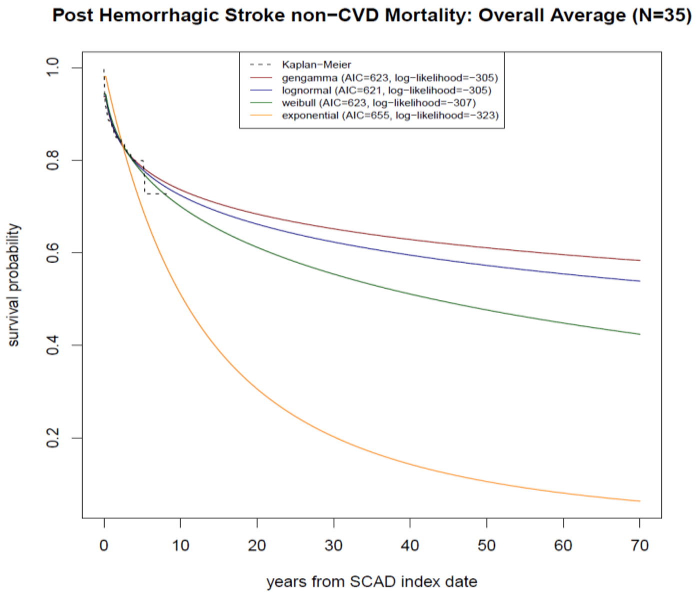

Supplement: Web appendix D [file heartjnl-2015-308850-s4.pdf]
