# Supplementary material for: Using electronic health records to predict costs and outcomes in stable coronary artery disease
Source: Heart. 2016 Feb 10;102(10):755–62. doi: 10.1136/heartjnl-2015-308850 (PMC4849559; doi:10.1136/heartjnl-2015-308850)
Supplement: Web appendix E [file heartjnl-2015-308850-s5.pdf]

Modelling lifetime costs and health outcomes for patients with stable coronary artery disease
Appendix E: Patient Profiles

Section 1: Patient Risk Deciles

Patient average covariate profiles based on deciles of 5yr risk of composite CVD primary endpoint											
Risk Decile	**1**	**2**	**3**	**4**	**5**	**6**	**7**	**8**	**9**	**10**	**Overall**
5 year risk (average across patients)	3.69%	5.70%	7.37%	9.15%	11.20%	13.71%	17.14%	22.14%	30.42%	52.37%	16.68%
5 year risk (at average covariate values)	3.46%	5.43%	6.95%	8.53%	10.36%	12.57%	15.64%	20.07%	27.23%	44.18%	11.64%
**Socio-demographic characteristics**											
Sex (% female)	64%	48%	42%	39%	37%	37%	38%	42%	44%	46%	44%
Age (if male)	49	55	59	62	65	67	71	74	77	81	67
Age (if female)	53	62	67	70	73	75	78	80	83	87	72
Age (weighted average)	52	59	62	65	68	70	73	76	80	84	69
Most deprived quintile (%)	15%	17%	18%	19%	20%	21%	21%	22%	22%	24%	20%
**SCAD diagnosis**											
Other CHD	11%	17%	20%	22%	24%	24%	25%	26%	25%	20%	23%
NSTEMI	0%	1%	3%	5%	8%	10%	12%	17%	23%	43%	10%
STEMI	1%	4%	8%	12%	13%	14%	13%	9%	6%	4%	7%
Unstable angina	10%	13%	12%	12%	12%	12%	13%	15%	17%	15%	14%
Stable angina	78%	65%	56%	49%	43%	39%	37%	34%	29%	18%	47%
**SCAD severity**											
PCI in last 6 months	9%	12%	13%	14%	13%	13%	11%	9%	6%	4%	9%
CABG in last 6 months	9%	7%	6%	5%	5%	4%	4%	3%	2%	1%	4%
Previous/recurrent MI	2%	6%	10%	14%	18%	23%	26%	29%	32%	43%	18%
Use of nitrates	10%	16%	19%	21%	24%	28%	33%	37%	43%	56%	28%
**Smoking status**											
Current smoker	31%	35%	36%	37%	38%	38%	37%	35%	32%	30%	35%
Ex-smoker	27%	30%	31%	32%	32%	33%	34%	34%	34%	34%	32%
Never smoked	41%	35%	33%	31%	30%	29%	29%	31%	33%	36%	33%
**Other CVD risk factors**											
Hypertension	69%	70%	71%	71%	72%	74%	76%	79%	83%	87%	76%
Diabetes	4%	8%	10%	12%	14%	16%	18%	21%	24%	32%	16%
Total cholesterol (mmol/L)	4.95	4.91	4.84	4.79	4.74	4.74	4.70	4.68	4.64	4.54	4.79
HDL (mmol/L)	1.41	1.37	1.35	1.35	1.35	1.35	1.36	1.37	1.37	1.35	1.37
**CVD co-morbidities**											
Heart failure	5%	7%	9%	12%	15%	19%	27%	37%	52%	73%	26%
Peripheral arterial disease	1%	2%	3%	4%	6%	8%	10%	13%	16%	25%	8%
Atrial fibrillation	3%	5%	7%	9%	10%	13%	16%	21%	29%	43%	15%
Stroke	0%	1%	1%	2%	3%	5%	8%	14%	22%	39%	9%
**Non-CVD co-morbidities**											
Chronic kidney disease	2%	2%	3%	4%	4%	5%	7%	9%	12%	20%	7%
Chronic obstructive pulmonary disease	20%	20%	20%	21%	22%	23%	25%	27%	28%	30%	23%
Cancer	4%	5%	6%	7%	8%	9%	11%	13%	14%	12%	9%
Chronic liver disease	0%	1%	1%	1%	1%	1%	1%	1%	1%	1%	1%
**Psychosocial characteristics**											
Depression at diagnosis	20%	17%	15%	15%	14%	14%	15%	17%	18%	21%	17%
Anxiety at diagnosis	7%	6%	6%	7%	7%	7%	8%	8%	10%	12%	8%
**Biomarkers**											
Heart rate (b.p.m.)	72	71	71	71	71	71	72	73	74	76	72
Creatinine (mmol/L)	88	92	95	96	98	100	101	104	109	125	100
White cell count (10^9/L)	6.81	7.05	7.19	7.31	7.44	7.54	7.62	7.76	7.88	8.22	7.46
Haemoglobin (g/100ml)	14.26	14.26	14.16	14.05	13.88	13.70	13.48	13.16	12.81	12.20	13.61

Section 2: Clinically selected patients

**Sample patient covariate profiles for 10 clinically selected patients**										
Patient Profile	**1**	**2**	**3**	**4**	**5**	**6**	**7**	**8**	**9**	**10**
5 year risk	3.68%	5.72%	7.59%	9.26%	11.48%	13.83%	17.41%	22.29%	30.44%	50.11%
**Socio-demographic characteristics**										
Sex	Female	Female	Male	Male	Male	Male	Male	Male	Male	Male
Age	53	62	59	62	65	67	71	74	76	81
Most deprived quintile	-	-	TRUE	-	-	TRUE			TRUE	-
**SCAD diagnosis**										
Other CHD	-	-	-	-	TRUE	TRUE	TRUE	TRUE	-	-
NSTEMI	-	-	-	-	-	-	-	-	TRUE	TRUE
STEMI	-	-	-	-	-	-	-	-	-	-
Unstable angina	-	-	-	-	-	-	-	-	-	-
Stable angina	TRUE	TRUE	TRUE	TRUE	-	-	-	-	-	-
**SCAD severity**										
PCI in last 6 months	-	-	-	-	-	-	-	-	-	-
CABG in last 6 months	-	-	-	-	-	-	-	-	-	-
Previous/recurrent MI	-	-	-	-	TRUE	-	TRUE	-	-	-
Use of nitrates	-	-	-	TRUE	-	TRUE	TRUE	TRUE	-	-
**Smoking Status**										
Current smoker	-	TRUE	TRUE	-	-	-	TRUE	TRUE	TRUE	-
Ex-smoker	-	-	-	TRUE	-	-	-	-	-	-
Never smoked	TRUE	-	-	-	-	TRUE	-	-	-	TRUE
**Other CVD risk factors**										
Hypertension	TRUE	TRUE	TRUE	TRUE	TRUE	TRUE	TRUE	TRUE	TRUE	TRUE
Diabetes	-	-	-	-	-	-	-	-	-	TRUE
Total cholesterol (mmol/L)	5.57	4.78	4.30	6.63	4.39	4.80	4.68	4.00	3.23	3.70
HDL (mmol/L)	1.83	1.27	1.39	1.30	1.20	0.71	2.10	0.99	1.61	0.93
**CVD co-morbidities**										
Heart failure	-	-	-	-	-	-	-	TRUE	TRUE	TRUE
Peripheral arterial disease	-	-	-	-	-	-	-	-	-	-
Atrial fibrillation	-	-	-	-	-	-	TRUE	-	TRUE	-
Stroke	-	-	-	-	-	-	-	-	-	-
**Non-CVD co-morbidities**										
Chronic kidney disease	-	-	-	-	-	-	-	-	-	-
Chronic obstructive pulmonary disease	-	TRUE	TRUE	-	-	-	TRUE	-	TRUE	-
Cancer	-	-	-	-	-	-	-	-	-	-
Chronic liver disease	-	-	-	-	-	-	-	-	-	-
**Psychosocial characteristics**										
Depression at diagnosis	-	TRUE	-	TRUE	-	-	-	-	-	TRUE
Anxiety at diagnosis	-	-	-	-	-	-	-	-	-	-
**Biomarkers**										
Heart rate (b.p.m.)	66	69	77	79	70	65	67	78	79	79
Creatinine (mmol/L)	94.35	116.34	94.00	103.00	99.83	85.00	94.00	113.00	92.54	114.00
White cell count (10^9/L)	10.20	7.75	10.35	8.18	8.37	5.24	7.16	9.30	6.13	9.50
Haemoglobin (g/100ml)	11.05	13.01	15.77	14.39	12.60	10.90	16.00	14.90	15.67	11.70

Section 3: Trial comparable patient populations

**Patient average covariate profiles CALIBER patients matching trial criteria**		
Trial	**pegasus**	**odyssey**
**Socio-demographic characteristics**		
Sex (% female)	40%	44%
Age (if male)	75	69
Age (if female)	78	70
Age (weighted average)	76	70
Most deprived quintile (%)	19%	20%
**SCAD diagnosis**		
Other CHD	0%	22%
NSTEMI	64%	12%
STEMI	36%	8%
Unstable angina	0%	13%
Stable angina	0%	45%
**SCAD severity**		
PCI in last 6 months	23%	10%
CABG in last 6 months	6%	5%
Previous/recurrent MI	27%	20%
Use of nitrates	27%	29%
**Smoking status**		
Current smoker	28%	34%
Ex-smoker	35%	32%
Never smoked	37%	33%
**Other CVD risk factors**		
Hypertension	68%	75%
Diabetes	22%	16%
Total cholesterol (mmol/L)	4.22	4.75
HDL (mmol/L)	1.32	1.36
**CVD co-morbidities**		
Heart failure	28%	26%
Peripheral arterial disease	11%	9%
Atrial fibrillation	20%	16%
Stroke	0%	10%
**Non-CVD co-morbidities**		
Chronic kidney disease	11%	7%
Chronic obstructive pulmonary disease	24%	24%
Cancer	11%	9%
Chronic liver disease	1%	1%
**Psychosocial characteristics**		
Depression at diagnosis	14%	17%
Anxiety at diagnosis	7%	8%
**Biomarkers**		
Heart rate (b.p.m.)	70	72
Creatinine (mmol/L)	106	101
White cell count (10^9/L)	7.64	7.48
Haemoglobin (g/100ml)	13.27	13.58
