# Supplementary material for: Using electronic health records to predict costs and outcomes in stable coronary artery disease
Source: Heart. 2016 Feb 10;102(10):755–62. doi: 10.1136/heartjnl-2015-308850 (PMC4849559; doi:10.1136/heartjnl-2015-308850)
Supplement: Web appendix F [file heartjnl-2015-308850-s6.pdf]

# Modelling lifetime costs and health outcomes for patients with stable coronary artery disease

## Appendix F: Full Results by Risk Decile and Clinical Profiles

### Section 1: Results by Risk Deciles

| | Risk Decile | | | | | | | | | |
|---|---|---|---|---|---|---|---|---|---|---|
| **Basecase** | **1** | **2** | **3** | **4** | **5** | **6** | **7** | **8** | **9** | **10** |
| Life years | 26.81 (26.63 to 26.98) | 19.62 (19.48 to 19.8) | 17.34 (17.18 to 17.53) | 15.63 (15.47 to 15.84) | 14.26 (14.08 to 14.49) | 13.03 (12.83 to 13.28) | 11.92 (11.69 to 12.21) | 10.48 (10.21 to 10.84) | 8.52 (8.19 to 8.94) | 5.51 (5.09 to 6.02) |
| QALYs* | 19.23 (18.06 to 20.09) | 14.08 (13.28 to 14.69) | 12.4 (11.7 to 12.95) | 11.13 (10.5 to 11.62) | 10.09 (9.52 to 10.55) | 9.16 (8.64 to 9.58) | 8.26 (7.76 to 8.67) | 7.13 (6.66 to 7.54) | 5.65 (5.2 to 6.06) | 3.51 (3.14 to 3.92) |
| QALYs** | 19.11 (18.06 to 19.93) | 13.97 (13.26 to 14.54) | 12.29 (11.66 to 12.8) | 11.01 (10.45 to 11.48) | 9.97 (9.44 to 10.41) | 9.03 (8.53 to 9.45) | 8.13 (7.65 to 8.53) | 6.99 (6.54 to 7.4) | 5.5 (5.09 to 5.89) | 3.34 (3.01 to 3.72) |
| Total Cost (£) | 116,888 (64,743 to 168,032) | 81,490 (54,858 to 108,206) | 73,057 (53,809 to 92,411) | 68,102 (53,588 to 83,062) | 64,521 (53,054 to 76,141) | 62,034 (53,567 to 71,316) | 61,435 (54,672 to 68,506) | 59,446 (53,872 to 65,167) | 54,345 (49,316 to 59,720) | 43,020 (37,731 to 48,842) |
| CVD Specific Cost (£) | 71,943 (28,717 to 113,960) | 52,034 (29,821 to 73,886) | 47,681 (31,396 to 63,800) | 45,251 (32,763 to 57,894) | 43,438 (33,616 to 53,321) | 42,266 (34,888 to 50,204) | 42,301 (36,394 to 48,452) | 41,366 (36,735 to 46,148) | 38,410 (34,488 to 42,629) | 31,199 (27,373 to 35,474) |
| CHD Specific Cost (£) | 46,921 (12,629 to 79,870) | 36,069 (18,387 to 53,211) | 33,892 (20,897 to 46,637) | 32,693 (22,676 to 42,679) | 31,741 (23,884 to 39,629) | 30,944 (24,902 to 37,252) | 30,793 (26,031 to 35,683) | 29,885 (26,263 to 33,699) | 27,533 (24,555 to 30,719) | 22,324 (19,620 to 25,304) |
| Discounted Life Years | 16.77 (16.69 to 16.85) | 13.66 (13.58 to 13.75) | 12.5 (12.41 to 12.61) | 11.56 (11.46 to 11.68) | 10.76 (10.65 to 10.89) | 9.99 (9.87 to 10.15) | 9.26 (9.11 to 9.44) | 8.27 (8.1 to 8.5) | 6.9 (6.67 to 7.17) | 4.67 (4.38 to 5.01) |
| Discounted QALYs* | 12.09 (11.46 to 12.6) | 9.84 (9.33 to 10.24) | 8.97 (8.51 to 9.34) | 8.25 (7.82 to 8.6) | 7.63 (7.23 to 7.96) | 7.04 (6.66 to 7.34) | 6.42 (6.06 to 6.73) | 5.64 (5.3 to 5.94) | 4.58 (4.25 to 4.88) | 2.98 (2.7 to 3.28) |
| Discounted QALYs** | 12.04 (11.45 to 12.53) | 9.77 (9.31 to 10.17) | 8.9 (8.47 to 9.25) | 8.18 (7.78 to 8.51) | 7.55 (7.17 to 7.87) | 6.95 (6.58 to 7.25) | 6.34 (5.98 to 6.63) | 5.55 (5.21 to 5.84) | 4.47 (4.16 to 4.76) | 2.85 (2.6 to 3.13) |
| Discounted Total Cost (£) | 62,210 (33,724 to 90,043) | 50,864 (34,490 to 67,270) | 48,046 (35,660 to 60,475) | 46,535 (37,121 to 56,164) | 45,429 (37,877 to 53,081) | 44,785 (39,150 to 50,876) | 45,283 (40,798 to 49,797) | 44,903 (41,160 to 48,628) | 42,436 (38,855 to 46,110) | 35,549 (31,679 to 39,615) |
| Discounted CVD Cost (£) | 37,857 (14,738 to 60,313) | 32,331 (18,671 to 45,751) | 31,288 (21,030 to 41,484) | 30,896 (22,893 to 38,965) | 30,584 (24,097 to 37,099) | 30,531 (25,641 to 35,726) | 31,211 (27,360 to 35,214) | 31,281 (28,197 to 34,361) | 30,024 (27,337 to 32,944) | 25,801 (22,935 to 28,739) |
| Discounted CHD Cost (£) | 25,316 (7,118 to 42,948) | 22,868 (12,077 to 33,360) | 22,639 (14,464 to 30,648) | 22,672 (16,260 to 29,078) | 22,657 (17,508 to 27,823) | 22,619 (18,653 to 26,761) | 22,946 (19,867 to 26,107) | 22,778 (20,345 to 25,211) | 21,646 (19,569 to 23,841) | 18,522 (16,462 to 20,605) |
| Time to first event (years) | 24.55 (24.31 to 24.76) | 17.8 (17.64 to 17.95) | 15.62 (15.47 to 15.75) | 13.98 (13.85 to 14.11) | 12.67 (12.54 to 12.8) | 11.49 (11.36 to 11.62) | 10.43 (10.29 to 10.57) | 9 (8.85 to 9.15) | 7.06 (6.91 to 7.22) | 4.07 (3.9 to 4.23) |
| MI as primary endpoint (%) | 6 (5.55 to 6.49) | 7.11 (6.73 to 7.49) | 8.06 (7.72 to 8.43) | 8.94 (8.61 to 9.29) | 9.84 (9.5 to 10.15) | 10.7 (10.39 to 11.01) | 11.59 (11.28 to 11.9) | 12.33 (12.01 to 12.64) | 12.89 (12.57 to 13.22) | 14.3 (13.87 to 14.73) |
| Ischaemic stroke as primary endpoint (%) | 5.51 (5.01 to 6.06) | 5.7 (5.34 to 6.11) | 6.06 (5.73 to 6.43) | 6.39 (6.07 to 6.74) | 6.8 (6.48 to 7.11) | 7.37 (7.05 to 7.68) | 8.29 (7.95 to 8.63) | 9.31 (8.96 to 9.68) | 10.07 (9.72 to 10.43) | 9.97 (9.58 to 10.38) |
| Haemorragic stroke as primary endpoint (%) | 0.67 (0.48 to 0.89) | 0.67 (0.54 to 0.81) | 0.71 (0.59 to 0.82) | 0.72 (0.62 to 0.84) | 0.74 (0.65 to 0.84) | 0.76 (0.67 to 0.86) | 0.79 (0.7 to 0.89) | 0.78 (0.69 to 0.88) | 0.7 (0.61 to 0.81) | 0.48 (0.4 to 0.57) |
| CVD Mortality (%) | 4.48 (3.45 to 5.55) | 6.6 (5.45 to 7.51) | 8.52 (7.22 to 9.47) | 10.39 (8.97 to 11.44) | 12.63 (11.07 to 13.85) | 15.48 (13.78 to 17.07) | 20.17 (18.17 to 22.63) | 26.29 (23.61 to 30.18) | 34.46 (30.65 to 39.32) | 45.95 (41.34 to 50.07) |
| Non-CVD Mortality (%) | 95.46 (94.4 to 96.49) | 93.4 (92.49 to 94.55) | 91.48 (90.53 to 92.78) | 89.6 (88.56 to 91.03) | 87.37 (86.15 to 88.93) | 84.52 (82.93 to 86.22) | 79.83 (77.37 to 81.83) | 73.71 (69.82 to 76.39) | 65.54 (60.68 to 69.35) | 54.05 (49.93 to 58.66) |

* 1 year decrement post event

** constant decrement post event

| | | | | | Risk Decile | | | | | |
|---|---|---|---|---|---|---|---|---|---|---|
| **Scenario HR=0.9** | **1** | **2** | **3** | **4** | **5** | **6** | **7** | **8** | **9** | **10** |
| Life years | 26.82<br>(26.65 to 26.98) | 19.65<br>(19.52 to 19.81) | 17.38<br>(17.24 to 17.56) | 15.69<br>(15.54 to 15.88) | 14.33<br>(14.17 to 14.55) | 13.12<br>(12.94 to 13.36) | 12.05<br>(11.84 to 12.33) | 10.64<br>(10.39 to 10.98) | 8.7<br>(8.38 to 9.1) | 5.65<br>(5.26 to 6.14) |
| QALYs* | 19.23<br>(18.06 to 20.1) | 14.1<br>(13.31 to 14.71) | 12.44<br>(11.73 to 12.98) | 11.18<br>(10.55 to 11.67) | 10.15<br>(9.58 to 10.6) | 9.22<br>(8.7 to 9.65) | 8.35<br>(7.85 to 8.76) | 7.24<br>(6.77 to 7.65) | 5.77<br>(5.32 to 6.17) | 3.6<br>(3.23 to 4) |
| QALYs** | 19.13<br>(18.06 to 19.95) | 14<br>(13.28 to 14.58) | 12.33<br>(11.7 to 12.84) | 11.07<br>(10.5 to 11.53) | 10.04<br>(9.51 to 10.47) | 9.11<br>(8.61 to 9.53) | 8.23<br>(7.75 to 8.63) | 7.12<br>(6.66 to 7.51) | 5.63<br>(5.23 to 6.02) | 3.44<br>(3.12 to 3.81) |
| Total Cost (£) | 116,326<br>(64,272 to 167,320) | 81,135<br>(54,510 to 107,802) | 72,807<br>(53,450 to 92,149) | 67,944<br>(53,306 to 82,935) | 64,464<br>(52,855 to 76,119) | 62,119<br>(53,512 to 71,443) | 61,757<br>(54,884 to 68,906) | 60,001<br>(54,431 to 65,716) | 55,094<br>(50,093 to 60,425) | 43,676<br>(38,531 to 49,287) |
| CVD Specific Cost (£) | 71,461<br>(28,231 to 113,373) | 51,699<br>(29,429 to 73,512) | 47,415<br>(31,048 to 63,543) | 45,047<br>(32,473 to 57,691) | 43,302<br>(33,392 to 53,276) | 42,226<br>(34,724 to 50,204) | 42,422<br>(36,418 to 48,621) | 41,648<br>(36,976 to 46,488) | 38,835<br>(34,907 to 43,039) | 31,585<br>(27,807 to 35,723) |
| CHD Specific Cost (£) | 46,551<br>(12,262 to 79,418) | 35,800<br>(18,125 to 52,914) | 33,670<br>(20,647 to 46,416) | 32,513<br>(22,469 to 42,510) | 31,610<br>(23,696 to 39,506) | 30,882<br>(24,783 to 37,211) | 30,844<br>(26,004 to 35,739) | 30,050<br>(26,380 to 33,921) | 27,801<br>(24,778 to 30,944) | 22,571<br>(19,899 to 25,446) |
| Discounted Life Years | 16.78<br>(16.71 to 16.85) | 13.68<br>(13.61 to 13.77) | 12.53<br>(12.45 to 12.63) | 11.6<br>(11.51 to 11.71) | 10.81<br>(10.71 to 10.94) | 10.06<br>(9.94 to 10.2) | 9.35<br>(9.21 to 9.52) | 8.39<br>(8.22 to 8.6) | 7.03<br>(6.81 to 7.28) | 4.78<br>(4.5 to 5.11) |
| Discounted QALYs* | 12.1<br>(11.47 to 12.61) | 9.85<br>(9.35 to 10.26) | 8.99<br>(8.53 to 9.36) | 8.28<br>(7.85 to 8.63) | 7.67<br>(7.27 to 8) | 7.08<br>(6.7 to 7.39) | 6.49<br>(6.13 to 6.79) | 5.72<br>(5.37 to 6.02) | 4.66<br>(4.33 to 4.96) | 3.05<br>(2.77 to 3.35) |
| Discounted QALYs** | 12.05<br>(11.46 to 12.54) | 9.79<br>(9.33 to 10.19) | 8.93<br>(8.5 to 9.29) | 8.22<br>(7.81 to 8.55) | 7.6<br>(7.22 to 7.91) | 7.01<br>(6.64 to 7.31) | 6.41<br>(6.05 to 6.7) | 5.63<br>(5.29 to 5.92) | 4.56<br>(4.25 to 4.85) | 2.93<br>(2.68 to 3.21) |
| Discounted Total Cost (£) | 61,970<br>(33,482 to 89,767) | 50,675<br>(34,265 to 67,062) | 47,897<br>(35,481 to 60,325) | 46,427<br>(36,937 to 56,057) | 45,373<br>(37,768 to 53,058) | 44,806<br>(39,089 to 50,926) | 45,446<br>(40,888 to 50,039) | 45,219<br>(41,455 to 48,916) | 42,906<br>(39,371 to 46,580) | 36,032<br>(32,152 to 40,063) |
| Discounted CVD Cost (£) | 37,639<br>(14,517 to 60,073) | 32,144<br>(18,461 to 45,550) | 31,123<br>(20,815 to 41,320) | 30,757<br>(22,693 to 38,849) | 30,477<br>(23,969 to 37,015) | 30,476<br>(25,520 to 35,707) | 31,250<br>(27,342 to 35,246) | 31,423<br>(28,289 to 34,525) | 30,276<br>(27,547 to 33,156) | 26,077<br>(23,251 to 28,952) |
| Discounted CHD Cost (£) | 25,144<br>(6,949 to 42,762) | 22,715<br>(11,922 to 33,208) | 22,501<br>(14,300 to 30,506) | 22,552<br>(16,095 to 28,937) | 22,559<br>(17,391 to 27,734) | 22,558<br>(18,566 to 26,735) | 22,951<br>(19,822 to 26,116) | 22,856<br>(20,366 to 25,330) | 21,803<br>(19,688 to 24,006) | 18,697<br>(16,654 to 20,772) |
| Time to first event (years) | 24.76<br>(24.53 to 24.96) | 17.99<br>(17.84 to 18.13) | 15.81<br>(15.67 to 15.93) | 14.18<br>(14.06 to 14.31) | 12.88<br>(12.76 to 13) | 11.72<br>(11.59 to 11.84) | 10.68<br>(10.55 to 10.81) | 9.28<br>(9.13 to 9.42) | 7.35<br>(7.19 to 7.5) | 4.3<br>(4.13 to 4.47) |
| MI as primary endpoint (%) | 5.44<br>(5.03 to 5.89) | 6.46<br>(6.11 to 6.81) | 7.33<br>(7.02 to 7.67) | 8.15<br>(7.84 to 8.47) | 8.98<br>(8.67 to 9.27) | 9.8<br>(9.5 to 10.08) | 10.65<br>(10.36 to 10.94) | 11.38<br>(11.09 to 11.68) | 11.99<br>(11.68 to 12.31) | 13.47<br>(13.05 to 13.87) |
| Ischaemic stroke as primary endpoint (%) | 5<br>(4.54 to 5.51) | 5.18<br>(4.86 to 5.56) | 5.52<br>(5.21 to 5.87) | 5.84<br>(5.54 to 6.16) | 6.22<br>(5.92 to 6.51) | 6.76<br>(6.46 to 7.05) | 7.64<br>(7.32 to 7.97) | 8.64<br>(8.3 to 8.99) | 9.43<br>(9.08 to 9.78) | 9.48<br>(9.1 to 9.89) |
| Haemorragic stroke as primary endpoint (%) | 0.67<br>(0.49 to 0.9) | 0.68<br>(0.55 to 0.82) | 0.71<br>(0.6 to 0.84) | 0.73<br>(0.63 to 0.85) | 0.75<br>(0.66 to 0.86) | 0.77<br>(0.68 to 0.87) | 0.81<br>(0.72 to 0.91) | 0.8<br>(0.71 to 0.9) | 0.73<br>(0.64 to 0.84) | 0.51<br>(0.43 to 0.6) |
| CVD Mortality (%) | 4.09<br>(3.14 to 5.07) | 6.02<br>(4.97 to 6.86) | 7.79<br>(6.59 to 8.66) | 9.52<br>(8.21 to 10.49) | 11.59<br>(10.15 to 12.72) | 14.24<br>(12.67 to 15.73) | 18.65<br>(16.77 to 20.96) | 24.46<br>(21.94 to 28.11) | 32.35<br>(28.74 to 36.88) | 43.8<br>(39.38 to 47.72) |
| Non-CVD Mortality (%) | 95.85<br>(94.88 to 96.79) | 93.98<br>(93.14 to 95.03) | 92.21<br>(91.34 to 93.41) | 90.48<br>(89.51 to 91.79) | 88.41<br>(87.28 to 89.85) | 85.76<br>(84.27 to 87.33) | 81.35<br>(79.04 to 83.23) | 75.54<br>(71.89 to 78.06) | 67.65<br>(63.12 to 71.26) | 56.2<br>(52.28 to 60.62) |

* 1 year decrement post event
** constant decrement post event

| | Risk Decile | | | | | | | | | |
|---|---|---|---|---|---|---|---|---|---|---|
| **Scenario HR=0.8** | **1** | **2** | **3** | **4** | **5** | **6** | **7** | **8** | **9** | **10** |
| Life years | 26.83 (26.67 to 26.97) | 19.68 (19.56 to 19.83) | 17.43 (17.3 to 17.59) | 15.75 (15.61 to 15.93) | 14.41 (14.26 to 14.61) | 13.22 (13.06 to 13.44) | 12.19 (11.99 to 12.44) | 10.81 (10.58 to 11.12) | 8.89 (8.59 to 9.26) | 5.81 (5.43 to 6.27) |
| QALYs* | 19.24 (18.07 to 20.11) | 14.12 (13.34 to 14.73) | 12.47 (11.77 to 13.01) | 11.22 (10.59 to 11.71) | 10.2 (9.63 to 10.65) | 9.3 (8.77 to 9.72) | 8.45 (7.95 to 8.86) | 7.36 (6.88 to 7.77) | 5.89 (5.45 to 6.29) | 3.71 (3.34 to 4.1) |
| QALYs** | 19.14 (18.06 to 19.97) | 14.03 (13.31 to 14.62) | 12.37 (11.74 to 12.88) | 11.12 (10.55 to 11.59) | 10.1 (9.57 to 10.53) | 9.19 (8.7 to 9.6) | 8.34 (7.85 to 8.74) | 7.24 (6.79 to 7.63) | 5.77 (5.36 to 6.15) | 3.56 (3.23 to 3.92) |
| Total Cost (£) | 115,756 (63,795 to 166,598) | 80,773 (54,120 to 107,389) | 72,551 (53,082 to 91,896) | 67,781 (53,014 to 82,803) | 64,405 (52,651 to 76,101) | 62,207 (53,455 to 71,622) | 62,097 (55,108 to 69,331) | 60,594 (54,973 to 66,325) | 55,914 (50,957 to 61,211) | 44,438 (39,264 to 49,876) |
| CVD Specific Cost (£) | 70,971 (27,738 to 112,777) | 51,358 (29,040 to 73,130) | 47,143 (30,692 to 63,247) | 44,839 (32,205 to 57,488) | 43,163 (33,161 to 53,177) | 42,185 (34,553 to 50,181) | 42,551 (36,444 to 48,767) | 41,950 (37,232 to 46,828) | 39,303 (35,345 to 43,456) | 32,038 (28,346 to 36,032) |
| CHD Specific Cost (£) | 46,175 (11,891 to 78,959) | 35,525 (17,858 to 52,611) | 33,442 (20,394 to 46,174) | 32,330 (22,210 to 42,337) | 31,475 (23,530 to 39,414) | 30,817 (24,670 to 37,225) | 30,900 (25,961 to 35,827) | 30,228 (26,517 to 34,109) | 28,096 (25,049 to 31,237) | 22,861 (20,245 to 25,719) |
| Discounted Life Years | 16.79 (16.73 to 16.86) | 13.7 (13.64 to 13.78) | 12.56 (12.49 to 12.66) | 11.64 (11.56 to 11.75) | 10.86 (10.77 to 10.98) | 10.13 (10.02 to 10.26) | 9.44 (9.31 to 9.6) | 8.5 (8.35 to 8.7) | 7.16 (6.96 to 7.4) | 4.91 (4.64 to 5.21) |
| Discounted QALYs* | 12.11 (11.47 to 12.62) | 9.87 (9.36 to 10.28) | 9.01 (8.55 to 9.39) | 8.31 (7.88 to 8.66) | 7.71 (7.3 to 8.03) | 7.13 (6.75 to 7.44) | 6.55 (6.19 to 6.85) | 5.8 (5.45 to 6.1) | 4.75 (4.42 to 5.05) | 3.13 (2.85 to 3.42) |
| Discounted QALYs** | 12.06 (11.47 to 12.56) | 9.82 (9.35 to 10.21) | 8.96 (8.52 to 9.32) | 8.25 (7.85 to 8.59) | 7.64 (7.26 to 7.96) | 7.06 (6.69 to 7.36) | 6.48 (6.13 to 6.78) | 5.72 (5.38 to 6.01) | 4.66 (4.35 to 4.94) | 3.02 (2.77 to 3.3) |
| Discounted Total Cost (£) | 61,727 (33,236 to 89,489) | 50,483 (34,036 to 66,850) | 47,745 (35,300 to 60,172) | 46,317 (36,748 to 55,994) | 45,314 (37,691 to 53,033) | 44,829 (39,027 to 51,018) | 45,617 (41,061 to 50,263) | 45,555 (41,805 to 49,261) | 43,414 (39,829 to 47,104) | 36,583 (32,756 to 40,495) |
| Discounted CVD Cost (£) | 37,418 (14,294 to 59,830) | 31,953 (18,248 to 45,346) | 30,956 (20,596 to 41,151) | 30,615 (22,490 to 38,731) | 30,368 (23,839 to 36,906) | 30,419 (25,391 to 35,688) | 31,291 (27,335 to 35,254) | 31,575 (28,357 to 34,715) | 30,551 (27,749 to 33,423) | 26,395 (23,621 to 29,239) |
| Discounted CHD Cost (£) | 24,970 (6,780 to 42,575) | 22,560 (11,764 to 33,053) | 22,361 (14,139 to 30,360) | 22,428 (15,925 to 28,821) | 22,459 (17,272 to 27,665) | 22,494 (18,470 to 26,702) | 22,957 (19,776 to 26,161) | 22,941 (20,424 to 25,455) | 21,974 (19,859 to 24,145) | 18,899 (16,854 to 20,921) |
| Time to first event (years) | 24.97 (24.76 to 25.15) | 18.18 (18.04 to 18.31) | 16.01 (15.88 to 16.12) | 14.39 (14.27 to 14.51) | 13.09 (12.98 to 13.21) | 11.95 (11.83 to 12.07) | 10.94 (10.81 to 11.07) | 9.57 (9.43 to 9.71) | 7.65 (7.49 to 7.81) | 4.56 (4.38 to 4.73) |
| MI as primary endpoint (%) | 4.87 (4.5 to 5.28) | 5.79 (5.48 to 6.11) | 6.58 (6.3 to 6.9) | 7.33 (7.05 to 7.63) | 8.1 (7.81 to 8.36) | 8.85 (8.58 to 9.12) | 9.66 (9.39 to 9.94) | 10.39 (10.11 to 10.67) | 11.03 (10.73 to 11.33) | 12.56 (12.16 to 12.94) |
| Ischaemic stroke as primary endpoint (%) | 4.48 (4.07 to 4.94) | 4.65 (4.36 to 5) | 4.97 (4.69 to 5.28) | 5.26 (4.99 to 5.56) | 5.62 (5.35 to 5.89) | 6.13 (5.85 to 6.4) | 6.96 (6.66 to 7.26) | 7.92 (7.6 to 8.26) | 8.72 (8.39 to 9.07) | 8.93 (8.56 to 9.33) |
| Haemorragic stroke as primary endpoint (%) | 0.68 (0.49 to 0.9) | 0.69 (0.55 to 0.83) | 0.72 (0.61 to 0.85) | 0.75 (0.64 to 0.86) | 0.76 (0.67 to 0.87) | 0.79 (0.7 to 0.89) | 0.83 (0.74 to 0.93) | 0.83 (0.73 to 0.93) | 0.76 (0.67 to 0.88) | 0.54 (0.45 to 0.64) |
| CVD Mortality (%) | 3.7 (2.84 to 4.57) | 5.43 (4.48 to 6.2) | 7.04 (5.96 to 7.84) | 8.62 (7.43 to 9.5) | 10.51 (9.2 to 11.54) | 12.95 (11.52 to 14.32) | 17.04 (15.31 to 19.19) | 22.49 (20.16 to 25.85) | 30.02 (26.63 to 34.22) | 41.36 (37.16 to 45.06) |
| Non-CVD Mortality (%) | 96.25 (95.37 to 97.1) | 94.56 (93.8 to 95.52) | 92.96 (92.16 to 94.04) | 91.38 (90.5 to 92.57) | 89.49 (88.46 to 90.8) | 87.05 (85.68 to 88.48) | 82.96 (80.81 to 84.69) | 77.5 (74.15 to 79.84) | 69.98 (65.78 to 73.37) | 58.64 (54.94 to 62.84) |

* 1 year decrement post event

** constant decrement post event

| | Risk Decile | | | | | | | | | |
|---|---|---|---|---|---|---|---|---|---|---|
| **Scenario HR=0.7** | **1** | **2** | **3** | **4** | **5** | **6** | **7** | **8** | **9** | **10** |
| Life years | 26.83 (26.69 to 26.97) | 19.71 (19.6 to 19.84) | 17.47 (17.35 to 17.62) | 15.81 (15.68 to 15.97) | 14.49 (14.36 to 14.67) | 13.33 (13.18 to 13.53) | 12.33 (12.15 to 12.56) | 10.99 (10.77 to 11.28) | 9.1 (8.82 to 9.44) | 5.99 (5.63 to 6.43) |
| QALYs* | 19.25 (18.08 to 20.11) | 14.14 (13.36 to 14.75) | 12.5 (11.8 to 13.04) | 11.26 (10.63 to 11.75) | 10.26 (9.68 to 10.71) | 9.37 (8.84 to 9.79) | 8.55 (8.04 to 8.95) | 7.49 (7.01 to 7.88) | 6.03 (5.59 to 6.42) | 3.82 (3.46 to 4.2) |
| QALYs** | 19.16 (18.08 to 19.99) | 14.06 (13.34 to 14.65) | 12.42 (11.77 to 12.93) | 11.18 (10.6 to 11.65) | 10.17 (9.64 to 10.6) | 9.28 (8.78 to 9.68) | 8.45 (7.97 to 8.85) | 7.38 (6.92 to 7.76) | 5.92 (5.51 to 6.29) | 3.69 (3.36 to 4.04) |
| Total Cost (£) | 115,177 (63,233 to 165,863) | 80,405 (53,673 to 106,949) | 72,288 (52,706 to 91,587) | 67,615 (52,735 to 82,667) | 64,345 (52,458 to 76,100) | 62,300 (53,397 to 71,782) | 62,454 (55,341 to 69,736) | 61,227 (55,536 to 66,892) | 56,812 (51,829 to 62,081) | 45,327 (40,267 to 50,662) |
| CVD Specific Cost (£) | 70,475 (27,238 to 112,172) | 51,010 (28,659 to 72,740) | 46,865 (30,328 to 63,028) | 44,624 (31,910 to 57,279) | 43,019 (32,975 to 53,046) | 42,144 (34,377 to 50,231) | 42,688 (36,474 to 48,945) | 42,274 (37,502 to 47,186) | 39,817 (35,815 to 43,938) | 32,570 (28,996 to 36,421) |
| CHD Specific Cost (£) | 45,795 (11,515 to 78,492) | 35,246 (17,587 to 52,302) | 33,210 (20,136 to 45,964) | 32,141 (21,989 to 42,159) | 31,337 (23,351 to 39,320) | 30,751 (24,552 to 37,240) | 30,959 (25,924 to 35,994) | 30,419 (26,620 to 34,296) | 28,422 (25,320 to 31,527) | 23,203 (20,580 to 25,947) |
| Discounted Life Years | 16.8 (16.74 to 16.86) | 13.73 (13.67 to 13.8) | 12.59 (12.53 to 12.68) | 11.68 (11.61 to 11.78) | 10.92 (10.83 to 11.02) | 10.19 (10.1 to 10.32) | 9.53 (9.42 to 9.68) | 8.62 (8.48 to 8.8) | 7.31 (7.12 to 7.52) | 5.05 (4.8 to 5.34) |
| Discounted QALYs* | 12.12 (11.48 to 12.62) | 9.89 (9.38 to 10.29) | 9.04 (8.58 to 9.42) | 8.34 (7.91 to 8.69) | 7.75 (7.34 to 8.07) | 7.18 (6.8 to 7.49) | 6.62 (6.25 to 6.92) | 5.88 (5.53 to 6.18) | 4.85 (4.51 to 5.14) | 3.22 (2.95 to 3.51) |
| Discounted QALYs** | 12.08 (11.48 to 12.57) | 9.84 (9.37 to 10.24) | 8.99 (8.55 to 9.36) | 8.29 (7.88 to 8.63) | 7.69 (7.3 to 8.01) | 7.12 (6.75 to 7.42) | 6.55 (6.2 to 6.85) | 5.81 (5.47 to 6.1) | 4.77 (4.45 to 5.05) | 3.12 (2.87 to 3.38) |
| Discounted Total Cost (£) | 61,482 (32,989 to 89,207) | 50,287 (33,804 to 66,634) | 47,589 (35,114 to 60,015) | 46,203 (36,555 to 55,929) | 45,254 (37,557 to 53,008) | 44,852 (38,964 to 51,038) | 45,796 (41,154 to 50,445) | 45,911 (42,152 to 49,609) | 43,966 (40,329 to 47,635) | 37,216 (33,401 to 41,056) |
| Discounted CVD Cost (£) | 37,194 (14,078 to 59,582) | 31,760 (18,041 to 45,139) | 30,785 (20,372 to 40,974) | 30,470 (22,281 to 38,569) | 30,256 (23,707 to 36,829) | 30,360 (25,265 to 35,669) | 31,335 (27,307 to 35,354) | 31,738 (28,495 to 34,931) | 30,850 (28,072 to 33,706) | 26,762 (24,002 to 29,540) |
| Discounted CHD Cost (£) | 24,794 (6,608 to 42,411) | 22,403 (11,605 to 32,895) | 22,218 (13,976 to 30,212) | 22,302 (15,752 to 28,716) | 22,356 (17,139 to 27,583) | 22,429 (18,374 to 26,666) | 22,965 (19,726 to 26,217) | 23,031 (20,502 to 25,571) | 22,161 (19,992 to 24,353) | 19,133 (17,135 to 21,134) |
| Time to first event (years) | 25.19 (25 to 25.35) | 18.38 (18.25 to 18.49) | 16.21 (16.09 to 16.32) | 14.6 (14.49 to 14.71) | 13.31 (13.21 to 13.42) | 12.19 (12.08 to 12.3) | 11.22 (11.09 to 11.34) | 9.88 (9.73 to 10.01) | 7.97 (7.82 to 8.13) | 4.84 (4.66 to 5.02) |
| MI as primary endpoint (%) | 4.3 (3.97 to 4.65) | 5.11 (4.83 to 5.4) | 5.82 (5.57 to 6.11) | 6.5 (6.24 to 6.76) | 7.19 (6.93 to 7.43) | 7.88 (7.63 to 8.12) | 8.63 (8.38 to 8.89) | 9.33 (9.07 to 9.6) | 9.99 (9.72 to 10.27) | 11.55 (11.17 to 11.92) |
| Ischaemic stroke as primary endpoint (%) | 3.96 (3.59 to 4.36) | 4.11 (3.85 to 4.42) | 4.4 (4.15 to 4.69) | 4.67 (4.43 to 4.94) | 5 (4.76 to 5.24) | 5.47 (5.22 to 5.71) | 6.24 (5.97 to 6.52) | 7.15 (6.85 to 7.46) | 7.96 (7.64 to 8.28) | 8.31 (7.95 to 8.69) |
| Haemorragic stroke as primary endpoint (%) | 0.69 (0.49 to 0.91) | 0.7 (0.56 to 0.84) | 0.73 (0.62 to 0.86) | 0.76 (0.65 to 0.87) | 0.78 (0.68 to 0.89) | 0.8 (0.71 to 0.91) | 0.85 (0.75 to 0.95) | 0.85 (0.75 to 0.96) | 0.8 (0.7 to 0.91) | 0.57 (0.48 to 0.68) |
| CVD Mortality (%) | 3.29 (2.53 to 4.07) | 4.83 (3.98 to 5.52) | 6.27 (5.3 to 6.98) | 7.68 (6.62 to 8.48) | 9.39 (8.21 to 10.33) | 11.61 (10.31 to 12.85) | 15.34 (13.79 to 17.31) | 20.38 (18.25 to 23.45) | 27.47 (24.35 to 31.3) | 38.57 (34.65 to 42.03) |
| Non-CVD Mortality (%) | 96.65 (95.87 to 97.41) | 95.17 (94.48 to 96.02) | 93.73 (93.02 to 94.7) | 92.32 (91.52 to 93.38) | 90.61 (89.67 to 91.79) | 88.39 (87.15 to 89.69) | 84.66 (82.69 to 86.21) | 79.62 (76.55 to 81.75) | 72.53 (68.7 to 75.65) | 61.43 (57.97 to 65.35) |

* 1 year decrement post event

** constant decrement post event

| | Risk Decile | | | | | | | | | |
|---|---|---|---|---|---|---|---|---|---|---|
| **Scenario HR=0.6** | **1** | **2** | **3** | **4** | **5** | **6** | **7** | **8** | **9** | **10** |
| Life years | 26.84 (26.71 to 26.96) | 19.74 (19.64 to 19.86) | 17.52 (17.41 to 17.65) | 15.87 (15.76 to 16.02) | 14.57 (14.45 to 14.73) | 13.43 (13.3 to 13.61) | 12.48 (12.32 to 12.69) | 11.18 (10.99 to 11.44) | 9.32 (9.07 to 9.63) | 6.2 (5.85 to 6.59) |
| QALYs* | 19.25 (18.09 to 20.12) | 14.17 (13.38 to 14.77) | 12.54 (11.84 to 13.07) | 11.31 (10.68 to 11.8) | 10.32 (9.74 to 10.77) | 9.45 (8.91 to 9.87) | 8.65 (8.14 to 9.05) | 7.62 (7.13 to 8.01) | 6.18 (5.73 to 6.57) | 3.96 (3.6 to 4.32) |
| QALYs** | 19.18 (18.08 to 20.01) | 14.09 (13.36 to 14.69) | 12.46 (11.81 to 12.99) | 11.23 (10.65 to 11.71) | 10.24 (9.7 to 10.67) | 9.36 (8.86 to 9.77) | 8.56 (8.08 to 8.97) | 7.52 (7.06 to 7.91) | 6.08 (5.66 to 6.45) | 3.83 (3.51 to 4.18) |
| Total Cost (£) | 114,589 (62,629 to 165,153) | 80,029 (53,217 to 106,536) | 72,020 (52,323 to 91,300) | 67,443 (52,434 to 82,527) | 64,283 (52,406 to 76,127) | 62,397 (53,341 to 71,876) | 62,832 (55,638 to 70,110) | 61,906 (56,117 to 67,617) | 57,799 (52,769 to 63,033) | 46,370 (41,440 to 51,583) |
| CVD Specific Cost (£) | 69,971 (26,732 to 111,558) | 50,656 (28,272 to 72,342) | 46,581 (30,007 to 62,763) | 44,404 (31,581 to 57,064) | 42,872 (32,790 to 52,950) | 42,103 (34,254 to 50,298) | 42,833 (36,510 to 49,136) | 42,623 (37,821 to 47,567) | 40,383 (36,362 to 44,471) | 33,199 (29,645 to 36,932) |
| CHD Specific Cost (£) | 45,409 (11,140 to 78,019) | 34,962 (17,312 to 51,987) | 32,973 (19,872 to 45,761) | 31,948 (21,773 to 41,976) | 31,194 (23,113 to 39,221) | 30,684 (24,409 to 37,185) | 31,022 (25,896 to 36,120) | 30,625 (26,717 to 34,582) | 28,780 (25,670 to 31,945) | 23,609 (21,053 to 26,297) |
| Discounted Life Years | 16.81 (16.76 to 16.87) | 13.75 (13.69 to 13.81) | 12.63 (12.57 to 12.7) | 11.73 (11.66 to 11.81) | 10.97 (10.89 to 11.07) | 10.27 (10.18 to 10.38) | 9.63 (9.53 to 9.76) | 8.75 (8.63 to 8.91) | 7.46 (7.29 to 7.66) | 5.21 (4.96 to 5.47) |
| Discounted QALYs* | 12.13 (11.49 to 12.63) | 9.9 (9.4 to 10.31) | 9.06 (8.6 to 9.44) | 8.38 (7.95 to 8.72) | 7.79 (7.38 to 8.11) | 7.23 (6.85 to 7.54) | 6.69 (6.31 to 6.99) | 5.97 (5.62 to 6.27) | 4.95 (4.61 to 5.24) | 3.33 (3.05 to 3.6) |
| Discounted QALYs** | 12.09 (11.48 to 12.59) | 9.86 (9.38 to 10.26) | 9.02 (8.58 to 9.39) | 8.33 (7.92 to 8.67) | 7.74 (7.35 to 8.05) | 7.18 (6.81 to 7.48) | 6.63 (6.27 to 6.93) | 5.91 (5.56 to 6.2) | 4.88 (4.56 to 5.16) | 3.24 (2.98 to 3.49) |
| Discounted Total Cost (£) | 61,233 (32,738 to 88,921) | 50,089 (33,569 to 66,414) | 47,430 (34,955 to 59,854) | 46,087 (36,357 to 55,823) | 45,193 (37,422 to 52,982) | 44,876 (38,931 to 51,071) | 45,984 (41,303 to 50,665) | 46,289 (42,418 to 49,997) | 44,565 (40,929 to 48,237) | 37,944 (34,172 to 41,744) |
| Discounted CVD Cost (£) | 36,968 (13,867 to 59,331) | 31,563 (17,844 to 44,928) | 30,611 (20,147 to 40,772) | 30,322 (22,069 to 38,417) | 30,141 (23,569 to 36,749) | 30,301 (25,165 to 35,650) | 31,382 (27,283 to 35,469) | 31,911 (28,661 to 35,135) | 31,176 (28,361 to 34,031) | 27,188 (24,466 to 29,914) |
| Discounted CHD Cost (£) | 24,616 (6,435 to 42,245) | 22,243 (11,423 to 32,734) | 22,072 (13,810 to 30,061) | 22,173 (15,605 to 28,608) | 22,250 (16,989 to 27,496) | 22,362 (18,271 to 26,630) | 22,973 (19,663 to 26,264) | 23,129 (20,522 to 25,716) | 22,365 (20,170 to 24,561) | 19,407 (17,431 to 21,396) |
| Time to first event (years) | 25.4 (25.24 to 25.55) | 18.57 (18.46 to 18.68) | 16.42 (16.31 to 16.51) | 14.81 (14.71 to 14.91) | 13.54 (13.44 to 13.64) | 12.43 (12.33 to 12.54) | 11.5 (11.38 to 11.62) | 10.2 (10.06 to 10.33) | 8.32 (8.17 to 8.48) | 5.17 (4.97 to 5.35) |
| MI as primary endpoint (%) | 3.71 (3.42 to 4.02) | 4.42 (4.18 to 4.67) | 5.05 (4.83 to 5.29) | 5.64 (5.41 to 5.87) | 6.25 (6.02 to 6.47) | 6.87 (6.65 to 7.09) | 7.56 (7.34 to 7.79) | 8.22 (7.98 to 8.46) | 8.88 (8.62 to 9.14) | 10.44 (10.09 to 10.78) |
| Ischaemic stroke as primary endpoint (%) | 3.42 (3.1 to 3.78) | 3.56 (3.34 to 3.84) | 3.82 (3.6 to 4.07) | 4.06 (3.85 to 4.3) | 4.36 (4.14 to 4.57) | 4.78 (4.56 to 5) | 5.48 (5.24 to 5.73) | 6.33 (6.05 to 6.61) | 7.12 (6.82 to 7.42) | 7.59 (7.26 to 7.96) |
| Haemorragic stroke as primary endpoint (%) | 0.69 (0.5 to 0.92) | 0.7 (0.57 to 0.85) | 0.74 (0.62 to 0.87) | 0.77 (0.66 to 0.89) | 0.79 (0.69 to 0.9) | 0.82 (0.73 to 0.93) | 0.87 (0.77 to 0.98) | 0.88 (0.78 to 1) | 0.83 (0.73 to 0.96) | 0.61 (0.52 to 0.73) |
| CVD Mortality (%) | 2.87 (2.2 to 3.58) | 4.22 (3.47 to 4.82) | 5.47 (4.62 to 6.1) | 6.72 (5.78 to 7.42) | 8.23 (7.19 to 9.06) | 10.2 (9.05 to 11.3) | 13.54 (12.16 to 15.32) | 18.12 (16.21 to 20.87) | 24.67 (21.84 to 28.1) | 35.36 (31.76 to 38.53) |
| Non-CVD Mortality (%) | 97.07 (96.36 to 97.73) | 95.78 (95.18 to 96.53) | 94.53 (93.9 to 95.38) | 93.28 (92.58 to 94.22) | 91.77 (90.94 to 92.81) | 89.8 (88.7 to 90.95) | 86.46 (84.68 to 87.84) | 81.88 (79.13 to 83.79) | 75.33 (71.9 to 78.16) | 64.64 (61.47 to 68.23) |

* 1 year decrement post event

** constant decrement post event

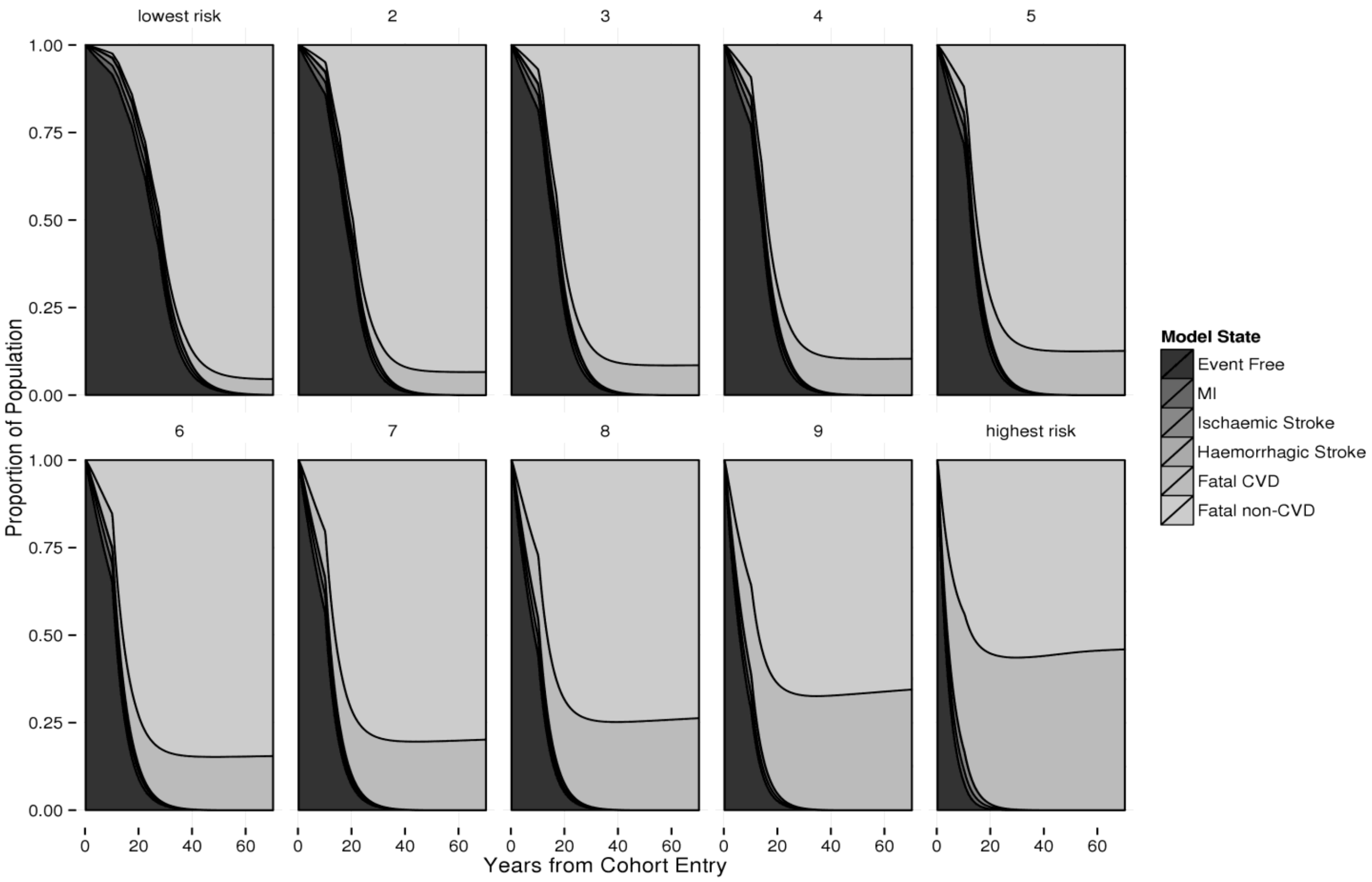
lowest risk
2
3
4
5
6
7
8
9
highest risk
1.00
0.75
0.50
0.25
0.00
Proportion of Population
0
20
40
60
Years from Cohort Entry
Model State
Event Free
MI
Ischaemic Stroke
Haemorrhagic Stroke
Fatal CVD
Fatal non-CVD

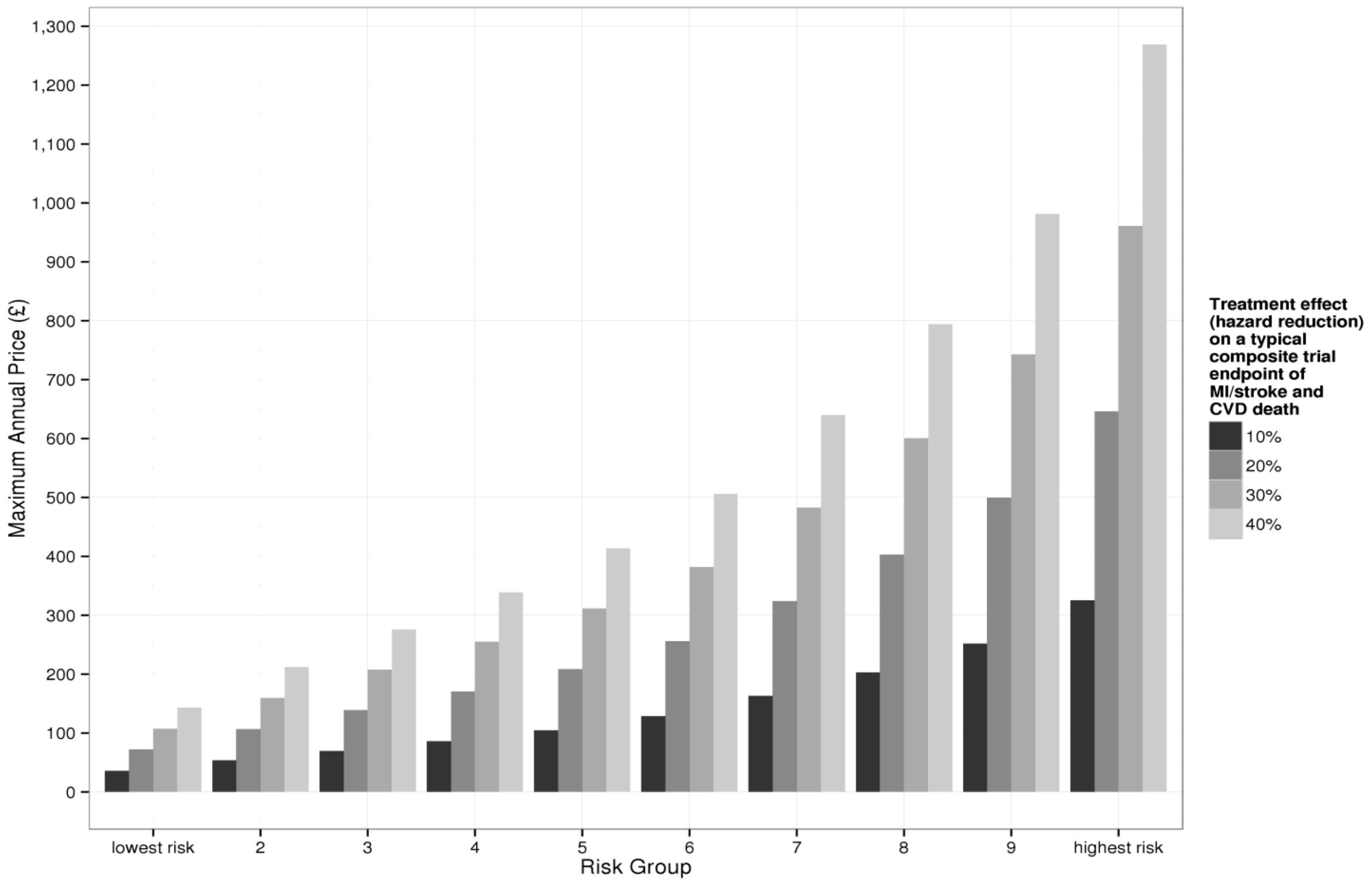

1,300
1,200
1,100
1,000
900
800
700
600
500
400
300
200
100
0
Maximum Annual Price (£)
lowest risk
2
3
4
5
6
7
8
9
highest risk
Risk Group
Treatment effect (hazard reduction) on a typical composite trial endpoint of MI/stroke and CVD death
10%
20%
30%
40%

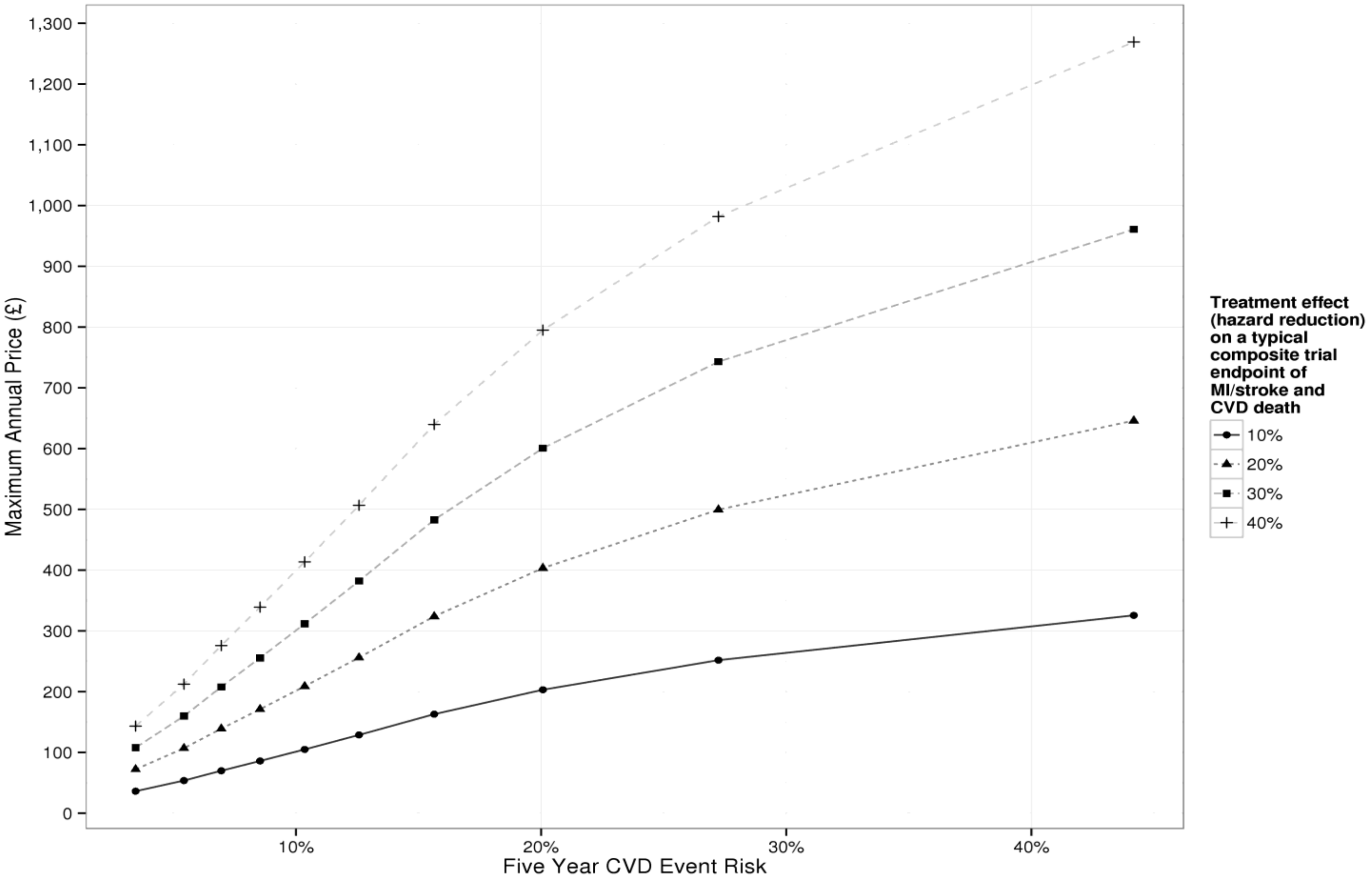
Maximum Annual Price (£)
1,300
1,200
1,100
1,000
900
800
700
600
500
400
300
200
100
0
10%
20%
30%
40%
Five Year CVD Event Risk
Treatment effect (hazard reduction) on a typical composite trial endpoint of MI/stroke and CVD death
10%
20%
30%
40%

**Section 2: Results Based on Selected Clinical Profiles**

| | Patient Profile | | | | | | | | | |
|---|---|---|---|---|---|---|---|---|---|---|
| **Basecase** | **1** | **2** | **3** | **4** | **5** | **6** | **7** | **8** | **9** | **10** |
| Life years | 25.61<br>(25.33 to 25.84) | 18.6<br>(18.29 to 18.87) | 18.41<br>(18.14 to 18.69) | 16.55<br>(16.23 to 16.88) | 15.01<br>(14.77 to 15.35) | 13.5<br>(13.11 to 13.9) | 12.41<br>(11.93 to 12.84) | 10.41<br>(9.98 to 10.82) | 9.04<br>(8.35 to 9.74) | 5.48<br>(4.99 to 6.07) |
| QALYs* | 18.51<br>(17.4 to 19.34) | 13.47<br>(12.69 to 14.09) | 13.36<br>(12.59 to 13.98) | 12.02<br>(11.33 to 12.57) | 10.9<br>(10.32 to 11.43) | 9.81<br>(9.22 to 10.34) | 9.01<br>(8.4 to 9.51) | 6.34<br>(5.73 to 6.84) | 5.5<br>(4.88 to 6.08) | 3.33<br>(2.95 to 3.75) |
| QALYs** | 18.4<br>(17.39 to 19.2) | 13.37<br>(12.65 to 13.99) | 13.26<br>(12.56 to 13.85) | 11.93<br>(11.29 to 12.47) | 10.76<br>(10.19 to 11.28) | 9.66<br>(9.1 to 10.18) | 8.84<br>(8.29 to 9.34) | 6.22<br>(5.63 to 6.71) | 5.34<br>(4.73 to 5.93) | 3.22<br>(2.83 to 3.62) |
| Total Cost (£) | 97,039<br>(49,388 to 143,478) | 82,964<br>(60,489 to 106,300) | 81,898<br>(58,414 to 106,229) | 56,502<br>(38,504 to 74,266) | 49,665<br>(35,461 to 63,564) | 44,497<br>(33,681 to 55,477) | 63,359<br>(55,253 to 72,133) | 50,352<br>(44,884 to 56,263) | 68,067<br>(61,550 to 74,877) | 42,529<br>(37,990 to 47,382) |
| CVD Specific Cost (£) | 58,657<br>(19,082 to 96,458) | 51,727<br>(32,888 to 71,095) | 52,296<br>(33,045 to 72,395) | 36,941<br>(21,875 to 51,656) | 33,987<br>(22,071 to 45,875) | 30,821<br>(21,659 to 40,068) | 48,232<br>(41,260 to 55,659) | 35,462<br>(31,008 to 40,395) | 51,822<br>(46,701 to 57,265) | 30,566<br>(27,267 to 33,973) |
| CHD Specific Cost (£) | 35,950<br>(4,550 to 65,628) | 34,931<br>(19,966 to 50,241) | 36,125<br>(20,724 to 52,053) | 24,542<br>(12,542 to 36,201) | 27,987<br>(18,501 to 37,393) | 25,520<br>(18,181 to 32,893) | 34,655<br>(29,155 to 40,573) | 26,699<br>(23,119 to 30,529) | 37,519<br>(33,671 to 41,528) | 24,279<br>(21,793 to 26,868) |
| Discounted Life Years | 16.27<br>(16.14 to 16.38) | 13.06<br>(12.89 to 13.22) | 12.93<br>(12.77 to 13.1) | 11.98<br>(11.78 to 12.19) | 11.16<br>(11 to 11.36) | 10.23<br>(9.97 to 10.49) | 9.59<br>(9.27 to 9.87) | 8.23<br>(7.93 to 8.51) | 7.26<br>(6.78 to 7.75) | 4.64<br>(4.27 to 5.07) |
| Discounted QALYs* | 11.82<br>(11.21 to 12.31) | 9.49<br>(8.98 to 9.91) | 9.42<br>(8.94 to 9.83) | 8.73<br>(8.27 to 9.1) | 8.12<br>(7.72 to 8.49) | 7.45<br>(7.04 to 7.82) | 6.97<br>(6.54 to 7.33) | 5.02<br>(4.57 to 5.39) | 4.42<br>(3.96 to 4.86) | 2.83<br>(2.53 to 3.15) |
| Discounted QALYs** | 11.77<br>(11.19 to 12.25) | 9.43<br>(8.96 to 9.84) | 9.36<br>(8.9 to 9.75) | 8.67<br>(8.24 to 9.05) | 8.03<br>(7.64 to 8.4) | 7.36<br>(6.96 to 7.72) | 6.86<br>(6.45 to 7.21) | 4.94<br>(4.5 to 5.31) | 4.31<br>(3.85 to 4.75) | 2.74<br>(2.43 to 3.06) |
| Discounted Total Cost (£) | 51,587<br>(25,308 to 76,991) | 52,614<br>(38,840 to 66,986) | 51,998<br>(37,350 to 67,021) | 36,514<br>(25,099 to 47,787) | 33,301<br>(24,026 to 42,212) | 30,690<br>(23,448 to 37,856) | 46,305<br>(40,908 to 52,152) | 37,856<br>(34,171 to 41,732) | 52,947<br>(48,530 to 57,385) | 35,084<br>(31,596 to 38,769) |
| Discounted CVD Cost (£) | 30,666<br>(9,115 to 51,409) | 32,587<br>(21,083 to 44,440) | 33,049<br>(21,154 to 45,279) | 23,805<br>(14,252 to 33,081) | 22,826<br>(14,984 to 30,469) | 21,306<br>(15,246 to 27,335) | 35,458<br>(30,850 to 40,399) | 26,713<br>(23,719 to 29,981) | 40,433<br>(36,920 to 44,039) | 25,227<br>(22,734 to 27,750) |
| Discounted CHD Cost (£) | 19,118<br>(2,134 to 35,549) | 22,399<br>(13,269 to 31,756) | 23,243<br>(13,848 to 32,937) | 16,060<br>(8,520 to 23,477) | 19,356<br>(13,131 to 25,442) | 18,118<br>(13,283 to 22,916) | 25,707<br>(22,029 to 29,616) | 20,326<br>(17,873 to 22,917) | 29,414<br>(26,822 to 32,126) | 20,117<br>(18,226 to 22,068) |
| Time to first event (years) | 23.4<br>(22.98 to 23.79) | 16.82<br>(16.4 to 17.24) | 16.31<br>(15.89 to 16.72) | 14.72<br>(14.25 to 15.21) | 13.24<br>(12.84 to 13.6) | 11.81<br>(11.26 to 12.34) | 10.65<br>(10.07 to 11.19) | 9.12<br>(8.65 to 9.61) | 7.31<br>(6.55 to 8.11) | 4.13<br>(3.62 to 4.65) |
| MI as primary endpoint (%) | 5.82<br>(4.96 to 6.76) | 5.93<br>(5.19 to 6.7) | 8.32<br>(7.44 to 9.19) | 8.86<br>(7.78 to 9.87) | 11.2<br>(10.3 to 12.21) | 11.51<br>(10.25 to 12.85) | 11.57<br>(10.33 to 12.86) | 10.17<br>(9.29 to 11.08) | 15<br>(13.4 to 16.61) | 16.81<br>(15.81 to 17.77) |
| Ischaemic stroke as primary endpoint (%) | 5.84<br>(4.9 to 6.72) | 6.99<br>(5.94 to 8.01) | 6.94<br>(6.07 to 7.88) | 6.15<br>(5.26 to 7.02) | 5.62<br>(4.96 to 6.33) | 5.94<br>(4.92 to 7.02) | 10.05<br>(8.5 to 11.65) | 7.32<br>(6.56 to 8.04) | 9.95<br>(8.61 to 11.27) | 5.89<br>(5.33 to 6.51) |
| Haemorragic stroke as primary endpoint (%) | 0.64<br>(0.42 to 0.99) | 0.66<br>(0.49 to 0.89) | 0.71<br>(0.56 to 0.89) | 0.74<br>(0.61 to 0.9) | 0.78<br>(0.65 to 0.91) | 0.76<br>(0.65 to 0.89) | 0.84<br>(0.7 to 0.99) | 0.83<br>(0.68 to 0.98) | 0.73<br>(0.59 to 0.91) | 0.53<br>(0.41 to 0.67) |
| CVD Mortality (%) | 4.51<br>(3.21 to 6.05) | 6.82<br>(5.25 to 8.71) | 9.64<br>(8.01 to 11.18) | 13.33<br>(11.34 to 15.28) | 15.84<br>(13.78 to 17.34) | 18.52<br>(16.22 to 20.65) | 22.08<br>(19.08 to 25.21) | 32.49<br>(29.49 to 35.87) | 35.15<br>(30.94 to 40.6) | 47.28<br>(44.52 to 50.44) |
| Non-CVD Mortality (%) | 95.44<br>(93.91 to 96.74) | 93.18<br>(91.29 to 94.75) | 90.36<br>(88.82 to 91.99) | 86.67<br>(84.72 to 88.66) | 84.16<br>(82.66 to 86.22) | 81.48<br>(79.35 to 83.78) | 77.92<br>(74.79 to 80.92) | 67.51<br>(64.13 to 70.51) | 64.85<br>(59.4 to 69.06) | 52.72<br>(49.56 to 55.48) |

* 1 year decrement post event

** constant decrement post event

| | Patient Profile | | | | | | | | | |
|---|---|---|---|---|---|---|---|---|---|---|
| **Scenario HR=0.9** | **1** | **2** | **3** | **4** | **5** | **6** | **7** | **8** | **9** | **10** |
| Life years | 25.62 (25.36 to 25.83) | 18.63 (18.34 to 18.89) | 18.46 (18.2 to 18.72) | 16.63 (16.33 to 16.94) | 15.12 (14.88 to 15.43) | 13.62 (13.24 to 14) | 12.55 (12.1 to 12.96) | 10.61 (10.2 to 11.01) | 9.22 (8.55 to 9.91) | 5.64 (5.14 to 6.22) |
| QALYs* | 18.52 (17.41 to 19.34) | 13.49 (12.71 to 14.11) | 13.4 (12.63 to 14.02) | 12.08 (11.39 to 12.63) | 10.98 (10.39 to 11.51) | 9.89 (9.32 to 10.42) | 9.11 (8.5 to 9.61) | 6.46 (5.86 to 6.96) | 5.61 (5 to 6.2) | 3.43 (3.04 to 3.85) |
| QALYs** | 18.42 (17.41 to 19.21) | 13.4 (12.66 to 14.02) | 13.3 (12.6 to 13.9) | 12 (11.36 to 12.54) | 10.84 (10.28 to 11.36) | 9.76 (9.2 to 10.28) | 8.95 (8.4 to 9.44) | 6.35 (5.76 to 6.84) | 5.46 (4.86 to 6.06) | 3.32 (2.93 to 3.73) |
| Total Cost (£) | 96,485 (48,868 to 142,792) | 82,651 (60,084 to 105,889) | 81,602 (58,059 to 105,970) | 56,373 (38,270 to 74,247) | 49,603 (35,239 to 63,636) | 44,507 (33,514 to 55,525) | 63,671 (55,554 to 72,503) | 51,081 (45,539 to 56,973) | 69,059 (62,597 to 75,932) | 43,211 (38,644 to 47,975) |
| CVD Specific Cost (£) | 58,183 (18,584 to 96,005) | 51,424 (32,565 to 70,764) | 51,983 (32,673 to 72,094) | 36,747 (21,576 to 51,557) | 33,841 (21,836 to 45,815) | 30,728 (21,468 to 40,049) | 48,389 (41,342 to 55,926) | 35,881 (31,279 to 40,876) | 52,485 (47,383 to 57,956) | 30,953 (27,600 to 34,392) |
| CHD Specific Cost (£) | 35,591 (4,179 to 65,238) | 34,700 (19,717 to 49,980) | 35,867 (20,411 to 51,780) | 24,355 (12,238 to 36,056) | 27,856 (18,299 to 37,339) | 25,433 (18,015 to 32,911) | 34,736 (29,139 to 40,717) | 26,985 (23,325 to 30,912) | 37,957 (34,083 to 42,009) | 24,549 (21,993 to 27,129) |
| Discounted Life Years | 16.28 (16.16 to 16.38) | 13.08 (12.92 to 13.23) | 12.97 (12.81 to 13.13) | 12.04 (11.85 to 12.23) | 11.23 (11.07 to 11.42) | 10.31 (10.06 to 10.56) | 9.68 (9.38 to 9.94) | 8.37 (8.09 to 8.64) | 7.39 (6.92 to 7.87) | 4.77 (4.39 to 5.2) |
| Discounted QALYs* | 11.83 (11.21 to 12.32) | 9.5 (9 to 9.92) | 9.45 (8.96 to 9.85) | 8.77 (8.31 to 9.15) | 8.17 (7.77 to 8.54) | 7.51 (7.11 to 7.87) | 7.04 (6.62 to 7.4) | 5.11 (4.65 to 5.48) | 4.51 (4.05 to 4.94) | 2.9 (2.6 to 3.23) |
| Discounted QALYs** | 11.78 (11.2 to 12.26) | 9.45 (8.98 to 9.86) | 9.39 (8.93 to 9.79) | 8.72 (8.29 to 9.1) | 8.09 (7.7 to 8.45) | 7.42 (7.03 to 7.79) | 6.94 (6.53 to 7.28) | 5.03 (4.59 to 5.4) | 4.4 (3.94 to 4.84) | 2.82 (2.51 to 3.15) |
| Discounted Total Cost (£) | 51,342 (25,061 to 76,713) | 52,441 (38,615 to 66,779) | 51,836 (37,149 to 66,887) | 36,425 (24,925 to 47,761) | 33,239 (23,924 to 42,230) | 30,666 (23,325 to 37,832) | 46,467 (40,985 to 52,319) | 38,295 (34,568 to 42,160) | 53,587 (49,192 to 58,106) | 35,583 (32,027 to 39,279) |
| Discounted CVD Cost (£) | 30,444 (8,896 to 51,182) | 32,412 (20,889 to 44,247) | 32,867 (20,914 to 45,124) | 23,674 (14,084 to 33,008) | 22,713 (14,813 to 30,411) | 21,220 (15,099 to 27,307) | 35,525 (30,895 to 40,460) | 26,953 (23,866 to 30,246) | 40,851 (37,331 to 44,467) | 25,499 (22,984 to 28,085) |
| Discounted CHD Cost (£) | 18,946 (1,943 to 35,380) | 22,264 (13,129 to 31,612) | 23,092 (13,665 to 32,810) | 15,936 (8,324 to 23,396) | 19,258 (12,998 to 25,375) | 18,044 (13,162 to 22,903) | 25,735 (21,999 to 29,689) | 20,492 (17,981 to 23,127) | 29,689 (27,075 to 32,416) | 20,306 (18,406 to 22,293) |
| Time to first event (years) | 23.6 (23.22 to 23.96) | 17.01 (16.61 to 17.4) | 16.54 (16.14 to 16.93) | 14.96 (14.51 to 15.42) | 13.49 (13.11 to 13.84) | 12.07 (11.54 to 12.58) | 10.93 (10.37 to 11.45) | 9.43 (8.96 to 9.9) | 7.62 (6.87 to 8.41) | 4.37 (3.84 to 4.91) |
| MI as primary endpoint (%) | 5.28 (4.49 to 6.13) | 5.39 (4.71 to 6.1) | 7.58 (6.77 to 8.39) | 8.08 (7.09 to 9.02) | 10.25 (9.41 to 11.19) | 10.55 (9.38 to 11.81) | 10.65 (9.48 to 11.86) | 9.41 (8.58 to 10.28) | 13.98 (12.43 to 15.54) | 15.85 (14.88 to 16.79) |
| Ischaemic stroke as primary endpoint (%) | 5.3 (4.45 to 6.11) | 6.36 (5.4 to 7.3) | 6.33 (5.53 to 7.2) | 5.62 (4.8 to 6.43) | 5.15 (4.53 to 5.82) | 5.46 (4.51 to 6.48) | 9.28 (7.82 to 10.79) | 6.8 (6.08 to 7.5) | 9.33 (8.04 to 10.61) | 5.61 (5.05 to 6.22) |
| Haemorragic stroke as primary endpoint (%) | 0.65 (0.42 to 0.99) | 0.67 (0.49 to 0.9) | 0.72 (0.57 to 0.9) | 0.76 (0.62 to 0.91) | 0.79 (0.67 to 0.93) | 0.78 (0.66 to 0.91) | 0.86 (0.72 to 1.01) | 0.86 (0.71 to 1.01) | 0.77 (0.61 to 0.94) | 0.56 (0.43 to 0.71) |
| CVD Mortality (%) | 4.12 (2.92 to 5.52) | 6.23 (4.78 to 7.98) | 8.83 (7.32 to 10.25) | 12.23 (10.38 to 14.04) | 14.56 (12.65 to 15.96) | 17.07 (14.92 to 19.08) | 20.45 (17.65 to 23.42) | 30.29 (27.41 to 33.48) | 33.07 (28.99 to 38.32) | 45.15 (42.44 to 48.26) |
| Non-CVD Mortality (%) | 95.83 (94.44 to 97.02) | 93.77 (92.02 to 95.21) | 91.17 (89.75 to 92.67) | 87.77 (85.96 to 89.62) | 85.44 (84.04 to 87.35) | 82.93 (80.92 to 85.08) | 79.55 (76.58 to 82.35) | 69.71 (66.52 to 72.59) | 66.93 (61.68 to 71.01) | 54.85 (51.74 to 57.56) |

* 1 year decrement post event
** constant decrement post event

| | | | | | Patient Profile | | | | | |
|---|---|---|---|---|---|---|---|---|---|---|
| **Scenario HR=0.8** | **1** | **2** | **3** | **4** | **5** | **6** | **7** | **8** | **9** | **10** |
| Life years | 25.63 (25.38 to 25.82) | 18.66 (18.39 to 18.91) | 18.51 (18.26 to 18.76) | 16.72 (16.43 to 17.01) | 15.22 (15 to 15.51) | 13.74 (13.38 to 14.1) | 12.7 (12.27 to 13.08) | 10.83 (10.43 to 11.21) | 9.43 (8.76 to 10.1) | 5.81 (5.3 to 6.4) |
| QALYs* | 18.52 (17.42 to 19.33) | 13.51 (12.74 to 14.14) | 13.44 (12.67 to 14.05) | 12.14 (11.45 to 12.69) | 11.06 (10.46 to 11.58) | 9.98 (9.41 to 10.5) | 9.21 (8.63 to 9.71) | 6.6 (5.99 to 7.1) | 5.74 (5.12 to 6.33) | 3.54 (3.14 to 3.96) |
| QALYs** | 18.44 (17.41 to 19.23) | 13.43 (12.69 to 14.05) | 13.35 (12.64 to 13.94) | 12.07 (11.43 to 12.61) | 10.93 (10.38 to 11.45) | 9.86 (9.3 to 10.37) | 9.07 (8.51 to 9.55) | 6.5 (5.9 to 6.99) | 5.6 (4.99 to 6.18) | 3.44 (3.04 to 3.86) |
| | | | | | | | | | | |
| Total Cost (£) | 95,922 (48,341 to 142,167) | 82,332 (59,672 to 105,476) | 81,298 (57,695 to 105,704) | 56,240 (38,071 to 74,228) | 49,540 (35,031 to 63,711) | 44,520 (33,403 to 55,609) | 63,999 (55,728 to 72,915) | 51,861 (46,260 to 57,771) | 70,142 (63,621 to 77,035) | 44,009 (39,340 to 48,836) |
| CVD Specific Cost (£) | 57,701 (18,079 to 95,545) | 51,115 (32,236 to 70,426) | 51,662 (32,253 to 71,783) | 36,547 (21,280 to 51,443) | 33,691 (21,594 to 45,727) | 30,633 (21,271 to 40,110) | 48,555 (41,424 to 56,149) | 36,330 (31,632 to 41,362) | 53,210 (48,062 to 58,630) | 31,410 (28,020 to 34,900) |
| CHD Specific Cost (£) | 35,228 (3,802 to 64,885) | 34,465 (19,463 to 49,734) | 35,603 (20,091 to 51,513) | 24,163 (11,976 to 35,919) | 27,720 (18,092 to 37,274) | 25,343 (17,843 to 32,903) | 34,822 (29,179 to 40,814) | 27,292 (23,487 to 31,333) | 38,435 (34,513 to 42,530) | 24,869 (22,288 to 27,583) |
| Discounted Life Years | 16.29 (16.18 to 16.39) | 13.1 (12.95 to 13.25) | 13 (12.86 to 13.15) | 12.1 (11.92 to 12.28) | 11.3 (11.15 to 11.48) | 10.4 (10.15 to 10.63) | 9.78 (9.5 to 10.03) | 8.52 (8.24 to 8.78) | 7.53 (7.06 to 8) | 4.91 (4.52 to 5.34) |
| Discounted QALYs* | 11.84 (11.22 to 12.33) | 9.52 (9.02 to 9.94) | 9.47 (8.99 to 9.88) | 8.81 (8.36 to 9.19) | 8.23 (7.82 to 8.59) | 7.57 (7.17 to 7.93) | 7.11 (6.7 to 7.47) | 5.2 (4.74 to 5.58) | 4.59 (4.13 to 5.03) | 2.99 (2.68 to 3.32) |
| Discounted QALYs** | 11.79 (11.2 to 12.27) | 9.47 (9 to 9.88) | 9.42 (8.96 to 9.82) | 8.77 (8.34 to 9.14) | 8.15 (7.76 to 8.51) | 7.49 (7.09 to 7.86) | 7.02 (6.61 to 7.36) | 5.13 (4.68 to 5.51) | 4.5 (4.04 to 4.93) | 2.92 (2.6 to 3.24) |
| Discounted Total Cost (£) | 51,093 (24,811 to 76,431) | 52,265 (38,386 to 66,568) | 51,670 (36,942 to 66,748) | 36,333 (24,782 to 47,735) | 33,176 (23,824 to 42,248) | 30,642 (23,215 to 37,869) | 46,635 (41,131 to 52,533) | 38,761 (35,000 to 42,676) | 54,278 (49,862 to 58,878) | 36,156 (32,540 to 39,848) |
| Discounted CVD Cost (£) | 30,219 (8,678 to 50,952) | 32,234 (20,692 to 44,050) | 32,682 (20,669 to 44,986) | 23,540 (13,891 to 32,934) | 22,597 (14,643 to 30,311) | 21,131 (14,947 to 27,279) | 35,595 (30,935 to 40,625) | 27,208 (24,014 to 30,581) | 41,304 (37,805 to 44,953) | 25,815 (23,300 to 28,466) |
| Discounted CHD Cost (£) | 18,771 (1,749 to 35,209) | 22,128 (12,987 to 31,465) | 22,937 (13,475 to 32,678) | 15,810 (8,126 to 23,289) | 19,158 (12,862 to 25,292) | 17,967 (13,037 to 22,902) | 25,765 (21,967 to 29,778) | 20,667 (18,126 to 23,356) | 29,987 (27,342 to 32,777) | 20,528 (18,602 to 22,585) |
| Time to first event (years) | 23.81 (23.46 to 24.15) | 17.2 (16.83 to 17.56) | 16.77 (16.4 to 17.14) | 15.21 (14.79 to 15.64) | 13.75 (13.39 to 14.08) | 12.33 (11.83 to 12.81) | 11.22 (10.68 to 11.71) | 9.75 (9.29 to 10.2) | 7.95 (7.21 to 8.73) | 4.64 (4.09 to 5.19) |
| MI as primary endpoint (%) | 4.73 (4.02 to 5.5) | 4.84 (4.22 to 5.48) | 6.82 (6.08 to 7.55) | 7.29 (6.38 to 8.15) | 9.26 (8.49 to 10.14) | 9.56 (8.48 to 10.72) | 9.68 (8.6 to 10.81) | 8.61 (7.83 to 9.42) | 12.88 (11.41 to 14.38) | 14.8 (13.86 to 15.71) |
| Ischaemic stroke as primary endpoint (%) | 4.75 (3.98 to 5.48) | 5.71 (4.84 to 6.58) | 5.71 (4.97 to 6.51) | 5.08 (4.33 to 5.82) | 4.67 (4.1 to 5.28) | 4.96 (4.08 to 5.9) | 8.47 (7.11 to 9.88) | 6.25 (5.57 to 6.91) | 8.66 (7.43 to 9.88) | 5.3 (4.75 to 5.89) |
| Haemorragic stroke as primary endpoint (%) | 0.66 (0.43 to 1) | 0.67 (0.5 to 0.91) | 0.73 (0.58 to 0.92) | 0.77 (0.63 to 0.93) | 0.81 (0.68 to 0.95) | 0.8 (0.67 to 0.93) | 0.88 (0.74 to 1.04) | 0.89 (0.73 to 1.04) | 0.8 (0.65 to 0.98) | 0.59 (0.46 to 0.76) |
| CVD Mortality (%) | 3.72 (2.64 to 5) | 5.63 (4.32 to 7.22) | 7.99 (6.62 to 9.28) | 11.08 (9.38 to 12.76) | 13.22 (11.47 to 14.52) | 15.55 (13.56 to 17.42) | 18.73 (16.11 to 21.53) | 27.91 (25.17 to 30.89) | 30.78 (26.85 to 35.85) | 42.71 (40.02 to 45.7) |
| Non-CVD Mortality (%) | 96.23 (94.96 to 97.31) | 94.37 (92.78 to 95.68) | 92.01 (90.72 to 93.38) | 88.92 (87.24 to 90.62) | 86.78 (85.48 to 88.53) | 84.45 (82.58 to 86.44) | 81.27 (78.47 to 83.89) | 72.09 (69.11 to 74.83) | 69.22 (64.15 to 73.15) | 57.29 (54.3 to 59.98) |

* 1 year decrement post event
** constant decrement post event

| | Patient Profile | | | | | | | | | |
|---|---|---|---|---|---|---|---|---|---|---|
| **Scenario HR=0.7** | **1** | **2** | **3** | **4** | **5** | **6** | **7** | **8** | **9** | **10** |
| Life years | 25.63<br>(25.41 to 25.81) | 18.69<br>(18.43 to 18.92) | 18.56<br>(18.33 to 18.79) | 16.81<br>(16.54 to 17.08) | 15.33<br>(15.12 to 15.6) | 13.87<br>(13.52 to 14.21) | 12.85<br>(12.45 to 13.2) | 11.06<br>(10.67 to 11.43) | 9.64<br>(8.98 to 10.3) | 6.02<br>(5.5 to 6.6) |
| QALYs* | 18.53<br>(17.44 to 19.34) | 13.53<br>(12.76 to 14.16) | 13.47<br>(12.72 to 14.08) | 12.21<br>(11.52 to 12.75) | 11.14<br>(10.54 to 11.65) | 10.07<br>(9.51 to 10.59) | 9.33<br>(8.75 to 9.82) | 6.74<br>(6.13 to 7.25) | 5.87<br>(5.24 to 6.46) | 3.66<br>(3.25 to 4.1) |
| QALYs** | 18.45<br>(17.43 to 19.25) | 13.46<br>(12.73 to 14.08) | 13.4<br>(12.67 to 13.98) | 12.14<br>(11.51 to 12.68) | 11.03<br>(10.46 to 11.53) | 9.97<br>(9.41 to 10.48) | 9.2<br>(8.65 to 9.67) | 6.65<br>(6.04 to 7.15) | 5.75<br>(5.13 to 6.34) | 3.57<br>(3.16 to 4) |
| Total Cost (£) | 95,350<br>(47,753 to 141,540) | 82,007<br>(59,251 to 105,219) | 80,986<br>(57,322 to 105,431) | 56,104<br>(37,866 to 74,146) | 49,475<br>(34,845 to 63,790) | 44,535<br>(33,299 to 55,682) | 64,344<br>(55,974 to 73,352) | 52,696<br>(47,028 to 58,676) | 71,327<br>(64,740 to 78,249) | 44,946<br>(40,207 to 49,952) |
| CVD Specific Cost (£) | 57,213<br>(17,566 to 95,080) | 50,800<br>(31,902 to 70,084) | 51,332<br>(31,868 to 71,465) | 36,342<br>(20,964 to 51,283) | 33,537<br>(21,345 to 45,638) | 30,536<br>(21,068 to 40,167) | 48,729<br>(41,505 to 56,357) | 36,811<br>(32,008 to 41,968) | 54,003<br>(48,813 to 59,461) | 31,952<br>(28,526 to 35,655) |
| CHD Specific Cost (£) | 34,859<br>(3,421 to 64,528) | 34,225<br>(19,204 to 49,464) | 35,332<br>(19,764 to 51,271) | 23,966<br>(11,740 to 35,774) | 27,581<br>(17,879 to 37,170) | 25,251<br>(17,666 to 32,894) | 34,912<br>(29,141 to 41,051) | 27,620<br>(23,666 to 31,793) | 38,960<br>(34,957 to 43,106) | 25,250<br>(22,617 to 28,090) |
| Discounted Life Years | 16.3<br>(16.2 to 16.39) | 13.12<br>(12.98 to 13.26) | 13.04<br>(12.9 to 13.18) | 12.16<br>(11.99 to 12.33) | 11.37<br>(11.24 to 11.54) | 10.48<br>(10.24 to 10.7) | 9.88<br>(9.62 to 10.12) | 8.68<br>(8.42 to 8.93) | 7.69<br>(7.23 to 8.15) | 5.07<br>(4.67 to 5.49) |
| Discounted QALYs* | 11.85<br>(11.23 to 12.33) | 9.54<br>(9.04 to 9.96) | 9.5<br>(9.01 to 9.9) | 8.86<br>(8.4 to 9.24) | 8.28<br>(7.87 to 8.64) | 7.63<br>(7.23 to 7.99) | 7.19<br>(6.78 to 7.54) | 5.3<br>(4.84 to 5.68) | 4.69<br>(4.22 to 5.12) | 3.09<br>(2.77 to 3.42) |
| Discounted QALYs** | 11.81<br>(11.22 to 12.28) | 9.49<br>(9.01 to 9.9) | 9.45<br>(8.99 to 9.85) | 8.82<br>(8.38 to 9.19) | 8.21<br>(7.82 to 8.56) | 7.56<br>(7.16 to 7.93) | 7.1<br>(6.71 to 7.45) | 5.23<br>(4.78 to 5.61) | 4.6<br>(4.15 to 5.03) | 3.02<br>(2.7 to 3.35) |
| Discounted Total Cost (£) | 50,840<br>(24,558 to 76,145) | 52,086<br>(38,153 to 66,352) | 51,500<br>(36,731 to 66,607) | 36,238<br>(24,636 to 47,649) | 33,111<br>(23,721 to 42,241) | 30,618<br>(23,119 to 37,930) | 46,812<br>(41,313 to 52,758) | 39,257<br>(35,408 to 43,235) | 55,027<br>(50,596 to 59,614) | 36,818<br>(33,232 to 40,644) |
| Discounted CVD Cost (£) | 29,992<br>(8,449 to 50,718) | 32,052<br>(20,491 to 43,850) | 32,492<br>(20,439 to 44,823) | 23,402<br>(13,723 to 32,860) | 22,477<br>(14,472 to 30,209) | 21,039<br>(14,790 to 27,285) | 35,668<br>(30,916 to 40,786) | 27,479<br>(24,203 to 30,925) | 41,796<br>(38,337 to 45,452) | 26,185<br>(23,622 to 28,883) |
| Discounted CHD Cost (£) | 18,595<br>(1,554 to 35,036) | 21,989<br>(12,843 to 31,315) | 22,780<br>(13,281 to 32,542) | 15,680<br>(7,972 to 23,191) | 19,056<br>(12,721 to 25,204) | 17,888<br>(12,907 to 22,891) | 25,797<br>(21,936 to 29,888) | 20,854<br>(18,233 to 23,604) | 30,311<br>(27,637 to 33,127) | 20,787<br>(18,831 to 22,867) |
| Time to first event (years) | 24.02<br>(23.7 to 24.33) | 17.39<br>(17.04 to 17.73) | 17.01<br>(16.66 to 17.36) | 15.46<br>(15.07 to 15.86) | 14.01<br>(13.68 to 14.32) | 12.61<br>(12.12 to 13.06) | 11.52<br>(11.02 to 11.98) | 10.08<br>(9.64 to 10.52) | 8.31<br>(7.57 to 9.07) | 4.94<br>(4.37 to 5.5) |
| MI as primary endpoint (%) | 4.17<br>(3.54 to 4.85) | 4.27<br>(3.72 to 4.85) | 6.04<br>(5.38 to 6.7) | 6.47<br>(5.65 to 7.25) | 8.24<br>(7.54 to 9.03) | 8.52<br>(7.54 to 9.58) | 8.67<br>(7.68 to 9.7) | 7.75<br>(7.03 to 8.51) | 11.7<br>(10.29 to 13.11) | 13.64<br>(12.74 to 14.52) |
| Ischaemic stroke as primary endpoint (%) | 4.2<br>(3.51 to 4.85) | 5.05<br>(4.28 to 5.83) | 5.07<br>(4.41 to 5.78) | 4.52<br>(3.84 to 5.19) | 4.16<br>(3.65 to 4.72) | 4.44<br>(3.64 to 5.29) | 7.61<br>(6.37 to 8.91) | 5.66<br>(5.03 to 6.28) | 7.92<br>(6.76 to 9.06) | 4.94<br>(4.42 to 5.51) |
| Haemorragic stroke as primary endpoint (%) | 0.66<br>(0.43 to 1.01) | 0.68<br>(0.5 to 0.92) | 0.74<br>(0.59 to 0.93) | 0.78<br>(0.64 to 0.94) | 0.82<br>(0.69 to 0.97) | 0.81<br>(0.69 to 0.95) | 0.91<br>(0.76 to 1.07) | 0.92<br>(0.76 to 1.08) | 0.84<br>(0.68 to 1.02) | 0.63<br>(0.5 to 0.81) |
| CVD Mortality (%) | 3.32<br>(2.34 to 4.48) | 5.02<br>(3.84 to 6.44) | 7.12<br>(5.89 to 8.29) | 9.89<br>(8.36 to 11.42) | 11.83<br>(10.24 to 13.01) | 13.95<br>(12.14 to 15.67) | 16.9<br>(14.47 to 19.44) | 25.35<br>(22.76 to 28.09) | 28.25<br>(24.52 to 33.12) | 39.91<br>(37.3 to 42.76) |
| Non-CVD Mortality (%) | 96.64<br>(95.47 to 97.61) | 94.98<br>(93.56 to 96.16) | 92.88<br>(91.71 to 94.11) | 90.11<br>(88.58 to 91.64) | 88.17<br>(86.99 to 89.76) | 86.05<br>(84.33 to 87.86) | 83.1<br>(80.56 to 85.53) | 74.65<br>(71.91 to 77.24) | 71.75<br>(66.88 to 75.48) | 60.09<br>(57.24 to 62.7) |

* 1 year decrement post event
** constant decrement post event

| | Patient Profile | | | | | | | | | |
|---|---|---|---|---|---|---|---|---|---|---|
| **Scenario HR=0.6** | **1** | **2** | **3** | **4** | **5** | **6** | **7** | **8** | **9** | **10** |
| Life years | 25.64 (25.43 to 25.81) | 18.72 (18.48 to 18.93) | 18.61 (18.39 to 18.83) | 16.9 (16.65 to 17.16) | 15.44 (15.25 to 15.69) | 14 (13.66 to 14.32) | 13.01 (12.64 to 13.34) | 11.3 (10.94 to 11.65) | 9.88 (9.24 to 10.54) | 6.25 (5.72 to 6.83) |
| QALYs* | 18.53 (17.46 to 19.35) | 13.56 (12.78 to 14.18) | 13.51 (12.76 to 14.12) | 12.28 (11.59 to 12.83) | 11.22 (10.63 to 11.73) | 10.17 (9.6 to 10.68) | 9.44 (8.87 to 9.93) | 6.89 (6.27 to 7.39) | 6.01 (5.39 to 6.6) | 3.8 (3.39 to 4.24) |
| QALYs** | 18.47 (17.44 to 19.27) | 13.49 (12.76 to 14.11) | 13.44 (12.72 to 14.03) | 12.22 (11.58 to 12.76) | 11.12 (10.55 to 11.62) | 10.07 (9.52 to 10.58) | 9.33 (8.79 to 9.8) | 6.8 (6.19 to 7.31) | 5.9 (5.28 to 6.49) | 3.72 (3.31 to 4.16) |
| Total Cost (£) | 94,770 (47,219 to 140,903) | 81,676 (58,823 to 104,885) | 80,665 (56,938 to 105,151) | 55,964 (37,658 to 73,948) | 49,409 (34,656 to 63,823) | 44,552 (33,195 to 55,889) | 64,708 (56,217 to 73,723) | 53,591 (47,784 to 59,809) | 72,628 (65,926 to 79,557) | 46,051 (41,137 to 51,152) |
| CVD Specific Cost (£) | 56,717 (17,046 to 94,607) | 50,479 (31,563 to 69,733) | 50,994 (31,442 to 71,139) | 36,131 (20,675 to 51,117) | 33,378 (21,088 to 45,544) | 30,436 (20,859 to 40,115) | 48,913 (41,574 to 56,703) | 37,327 (32,307 to 42,633) | 54,873 (49,624 to 60,397) | 32,597 (29,054 to 36,334) |
| CHD Specific Cost (£) | 34,485 (3,034 to 64,166) | 33,981 (18,939 to 49,234) | 35,054 (19,429 to 50,946) | 23,763 (11,499 to 35,597) | 27,437 (17,659 to 37,111) | 25,156 (17,483 to 32,847) | 35,007 (29,125 to 41,280) | 27,972 (23,854 to 32,253) | 39,535 (35,430 to 43,770) | 25,705 (23,009 to 28,568) |
| Discounted Life Years | 16.31 (16.22 to 16.4) | 13.15 (13.01 to 13.28) | 13.07 (12.94 to 13.21) | 12.22 (12.06 to 12.38) | 11.45 (11.32 to 11.6) | 10.57 (10.34 to 10.78) | 9.99 (9.74 to 10.21) | 8.84 (8.6 to 9.08) | 7.85 (7.4 to 8.3) | 5.24 (4.84 to 5.66) |
| Discounted QALYs* | 11.85 (11.24 to 12.34) | 9.55 (9.06 to 9.97) | 9.53 (9.03 to 9.93) | 8.9 (8.45 to 9.28) | 8.34 (7.92 to 8.69) | 7.69 (7.3 to 8.05) | 7.26 (6.86 to 7.61) | 5.4 (4.94 to 5.78) | 4.79 (4.32 to 5.22) | 3.19 (2.87 to 3.53) |
| Discounted QALYs** | 11.82 (11.23 to 12.3) | 9.52 (9.04 to 9.93) | 9.49 (9.01 to 9.89) | 8.87 (8.43 to 9.25) | 8.28 (7.88 to 8.63) | 7.63 (7.24 to 7.99) | 7.19 (6.81 to 7.53) | 5.34 (4.89 to 5.72) | 4.71 (4.25 to 5.15) | 3.13 (2.81 to 3.47) |
| Discounted Total Cost (£) | 50,584 (24,303 to 75,854) | 51,903 (37,916 to 66,162) | 51,327 (36,516 to 66,462) | 36,142 (24,488 to 47,536) | 33,044 (23,585 to 42,216) | 30,594 (23,020 to 37,973) | 46,996 (41,366 to 53,069) | 39,783 (35,838 to 43,908) | 55,840 (51,382 to 60,404) | 37,585 (33,889 to 41,411) |
| Discounted CVD Cost (£) | 29,761 (8,221 to 50,480) | 31,868 (20,286 to 43,646) | 32,299 (20,207 to 44,658) | 23,261 (13,539 to 32,797) | 22,355 (14,311 to 30,135) | 20,945 (14,628 to 27,275) | 35,745 (30,895 to 40,959) | 27,768 (24,445 to 31,302) | 42,329 (38,845 to 46,060) | 26,617 (23,951 to 29,343) |
| Discounted CHD Cost (£) | 18,416 (1,374 to 34,848) | 21,848 (12,697 to 31,162) | 22,619 (13,083 to 32,403) | 15,548 (7,815 to 23,110) | 18,950 (12,577 to 25,131) | 17,807 (12,773 to 22,833) | 25,830 (21,899 to 30,004) | 21,053 (18,329 to 23,888) | 30,662 (27,953 to 33,524) | 21,092 (19,070 to 23,216) |
| Time to first event (years) | 24.23 (23.95 to 24.51) | 17.59 (17.26 to 17.9) | 17.26 (16.93 to 17.58) | 15.72 (15.36 to 16.09) | 14.29 (13.98 to 14.57) | 12.89 (12.43 to 13.31) | 11.84 (11.37 to 12.27) | 10.44 (10.02 to 10.85) | 8.69 (7.97 to 9.44) | 5.27 (4.7 to 5.85) |
| MI as primary endpoint (%) | 3.6 (3.05 to 4.2) | 3.7 (3.22 to 4.2) | 5.24 (4.66 to 5.82) | 5.62 (4.9 to 6.32) | 7.18 (6.56 to 7.89) | 7.44 (6.57 to 8.39) | 7.6 (6.72 to 8.54) | 6.84 (6.19 to 7.53) | 10.42 (9.12 to 11.73) | 12.35 (11.5 to 13.19) |
| Ischaemic stroke as primary endpoint (%) | 3.63 (3.03 to 4.2) | 4.38 (3.7 to 5.06) | 4.4 (3.82 to 5.04) | 3.94 (3.34 to 4.53) | 3.64 (3.18 to 4.13) | 3.89 (3.18 to 4.65) | 6.7 (5.59 to 7.88) | 5.02 (4.44 to 5.59) | 7.1 (6.02 to 8.17) | 4.52 (4.03 to 5.07) |
| Haemorragic stroke as primary endpoint (%) | 0.67 (0.44 to 1.02) | 0.69 (0.51 to 0.93) | 0.75 (0.59 to 0.95) | 0.8 (0.65 to 0.96) | 0.84 (0.7 to 0.98) | 0.83 (0.7 to 0.98) | 0.93 (0.79 to 1.1) | 0.95 (0.79 to 1.11) | 0.88 (0.71 to 1.07) | 0.68 (0.53 to 0.86) |
| CVD Mortality (%) | 2.9 (2.04 to 3.97) | 4.38 (3.35 to 5.65) | 6.23 (5.13 to 7.26) | 8.66 (7.3 to 10.02) | 10.38 (8.97 to 11.43) | 12.28 (10.65 to 13.82) | 14.95 (12.77 to 17.27) | 22.57 (20.19 to 25.09) | 25.44 (21.99 to 29.88) | 36.67 (34.08 to 39.35) |
| Non-CVD Mortality (%) | 97.05 (95.99 to 97.91) | 95.61 (94.35 to 96.65) | 93.77 (92.74 to 94.87) | 91.34 (89.98 to 92.7) | 89.62 (88.57 to 91.03) | 87.72 (86.18 to 89.35) | 85.05 (82.73 to 87.23) | 77.43 (74.91 to 79.81) | 74.56 (70.12 to 78.01) | 63.33 (60.65 to 65.92) |

* 1 year decrement post event

** constant decrement post event

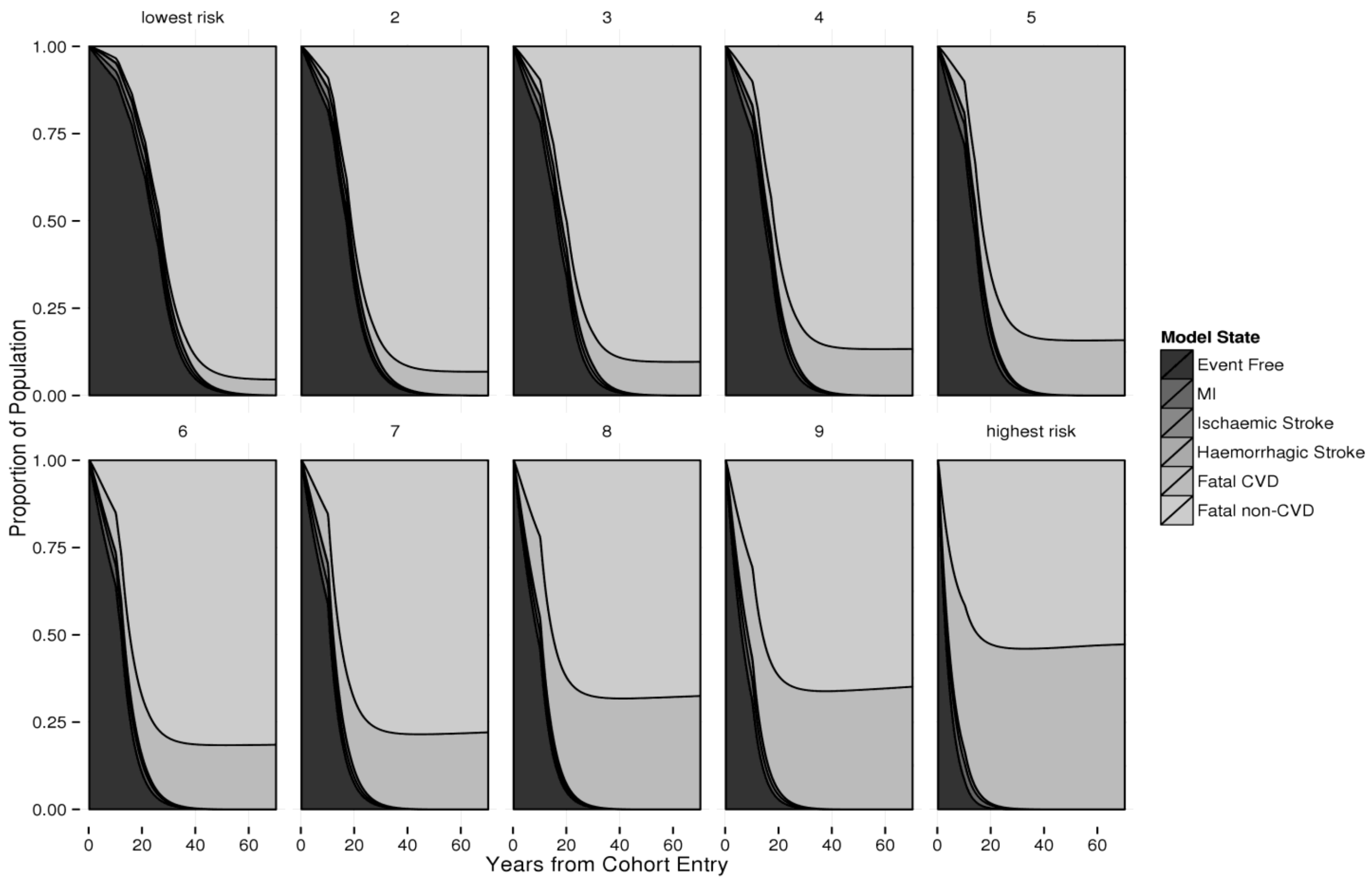

lowest risk
2
3
4
5
6
7
8
9
highest risk
1.00
0.75
0.50
0.25
0.00
0
20
40
60
Proportion of Population
Years from Cohort Entry
Model State
Event Free
MI
Ischaemic Stroke
Haemorrhagic Stroke
Fatal CVD
Fatal non-CVD

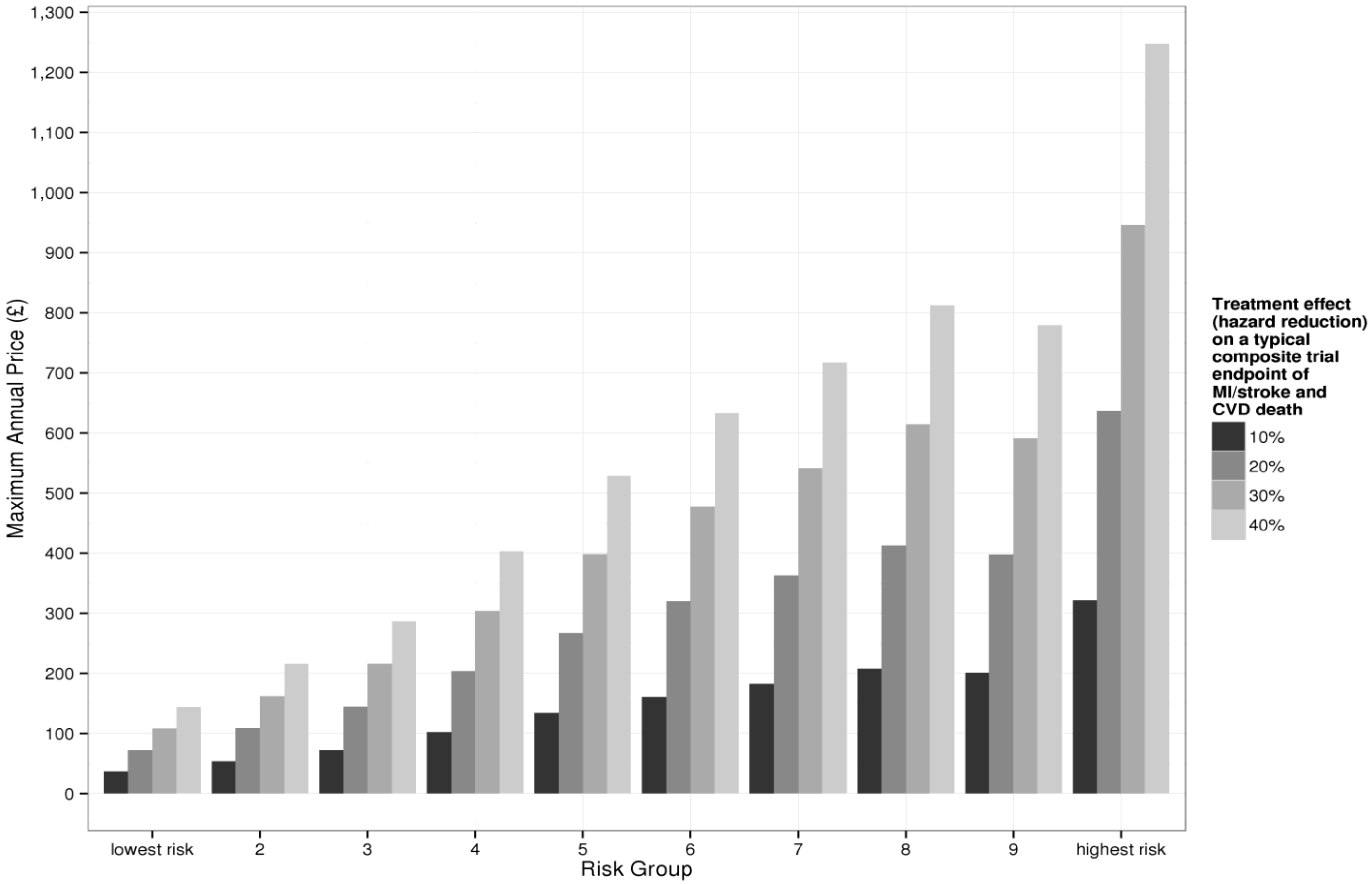

Maximum Annual Price (£)
1,300
1,200
1,100
1,000
900
800
700
600
500
400
300
200
100
0
lowest risk
2
3
4
5
6
7
8
9
highest risk
Risk Group
Treatment effect (hazard reduction) on a typical composite trial endpoint of MI/stroke and CVD death
10%
20%
30%
40%

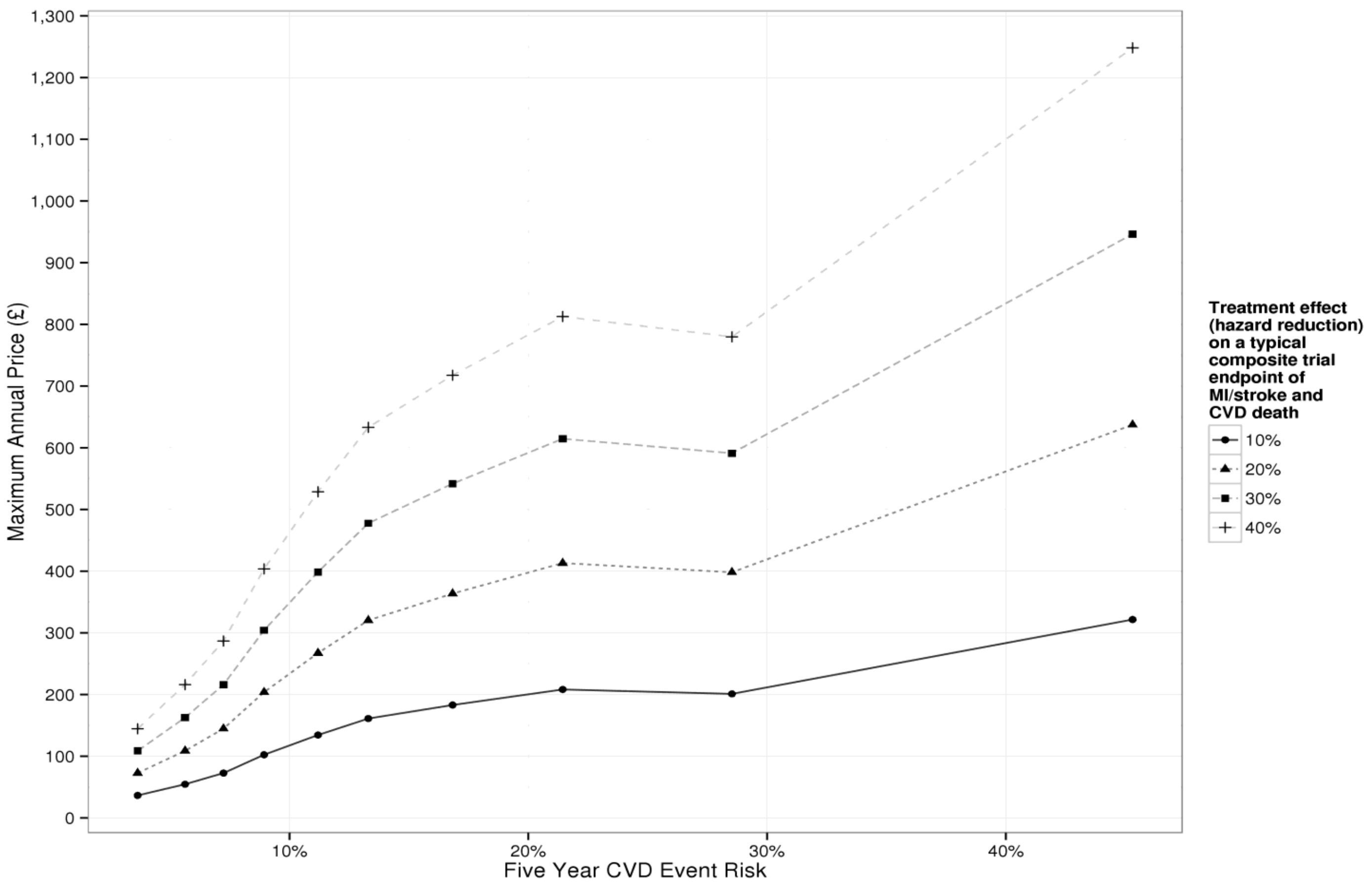

Maximum Annual Price (£)
1,300
1,200
1,100
1,000
900
800
700
600
500
400
300
200
100
0
10%
20%
30%
40%
Five Year CVD Event Risk
Treatment effect (hazard reduction) on a typical composite trial endpoint of MI/stroke and CVD death
10%
20%
30%
40%

**Section 3: Results for trial comparable patients**

| | pegasus | | | | | odyssey | | | | |
|---|---|---|---|---|---|---|---|---|---|---|
| **Scenario HR** | **1** | **0.9** | **0.8** | **0.7** | **0.6** | **1** | **0.9** | **0.8** | **0.7** | **0.6** |
| Life years | 10.97<br>(10.65 to 11.32) | 11.14<br>(10.83 to 11.47) | 11.31<br>(11.02 to 11.62) | 11.5<br>(11.22 to 11.79) | 11.7<br>(11.43 to 11.96) | 13.18<br>(12.99 to 13.43) | 13.27<br>(13.09 to 13.5) | 13.36<br>(13.19 to 13.57) | 13.46<br>(13.3 to 13.65) | 13.56<br>(13.42 to 13.73) |
| QALYs* | 7.59<br>(7.09 to 8.01) | 7.7<br>(7.21 to 8.12) | 7.83<br>(7.32 to 8.24) | 7.96<br>(7.44 to 8.37) | 8.09<br>(7.57 to 8.51) | 9.16<br>(8.63 to 9.6) | 9.22<br>(8.69 to 9.65) | 9.29<br>(8.75 to 9.72) | 9.36<br>(8.82 to 9.78) | 9.43<br>(8.88 to 9.85) |
| QALYs** | 7.44<br>(6.95 to 7.86) | 7.57<br>(7.07 to 7.99) | 7.7<br>(7.19 to 8.12) | 7.84<br>(7.34 to 8.27) | 7.99<br>(7.48 to 8.42) | 9.03<br>(8.52 to 9.45) | 9.1<br>(8.59 to 9.52) | 9.18<br>(8.67 to 9.6) | 9.26<br>(8.74 to 9.67) | 9.34<br>(8.82 to 9.75) |
| Total Cost (£) | 63,576<br>(57,426 to 69,784) | 64,058<br>(57,853 to 70,298) | 64,575<br>(58,308 to 70,873) | 65,131<br>(58,761 to 71,536) | 65,729<br>(59,306 to 72,117) | 65,510<br>(56,422 to 75,258) | 65,563<br>(56,366 to 75,362) | 65,619<br>(56,275 to 75,470) | 65,678<br>(56,223 to 75,587) | 65,740<br>(56,239 to 75,728) |
| CVD Specific Cost (£) | 45,358<br>(40,271 to 50,611) | 45,578<br>(40,412 to 50,892) | 45,815<br>(40,616 to 51,180) | 46,073<br>(40,711 to 51,439) | 46,352<br>(40,815 to 51,844) | 44,459<br>(36,587 to 52,829) | 44,397<br>(36,440 to 52,818) | 44,334<br>(36,257 to 52,805) | 44,269<br>(36,088 to 52,731) | 44,203<br>(35,976 to 52,656) |
| CHD Specific Cost (£) | 35,489<br>(31,406 to 39,666) | 35,604<br>(31,416 to 39,804) | 35,730<br>(31,448 to 39,965) | 35,866<br>(31,524 to 40,185) | 36,016<br>(31,606 to 40,388) | 31,905<br>(25,558 to 38,565) | 31,826<br>(25,394 to 38,523) | 31,745<br>(25,262 to 38,442) | 31,662<br>(25,098 to 38,363) | 31,576<br>(24,929 to 38,327) |
| Discounted Life Years | 8.61<br>(8.4 to 8.84) | 8.73<br>(8.52 to 8.94) | 8.85<br>(8.65 to 9.05) | 8.97<br>(8.78 to 9.16) | 9.1<br>(8.93 to 9.28) | 10.09<br>(9.97 to 10.24) | 10.15<br>(10.04 to 10.29) | 10.21<br>(10.11 to 10.34) | 10.28<br>(10.18 to 10.4) | 10.34<br>(10.26 to 10.45) |
| Discounted QALYs* | 5.97<br>(5.6 to 6.28) | 6.05<br>(5.68 to 6.36) | 6.13<br>(5.76 to 6.44) | 6.22<br>(5.84 to 6.53) | 6.31<br>(5.93 to 6.62) | 7.03<br>(6.65 to 7.35) | 7.07<br>(6.69 to 7.39) | 7.11<br>(6.73 to 7.43) | 7.16<br>(6.77 to 7.48) | 7.21<br>(6.81 to 7.52) |
| Discounted QALYs** | 5.86<br>(5.5 to 6.18) | 5.95<br>(5.59 to 6.27) | 6.04<br>(5.67 to 6.36) | 6.14<br>(5.77 to 6.46) | 6.24<br>(5.87 to 6.56) | 6.94<br>(6.57 to 7.25) | 6.99<br>(6.61 to 7.3) | 7.04<br>(6.67 to 7.35) | 7.1<br>(6.72 to 7.4) | 7.15<br>(6.78 to 7.46) |
| Discounted Total Cost (£) | 47,695<br>(43,550 to 51,732) | 47,954<br>(43,716 to 51,981) | 48,231<br>(43,972 to 52,314) | 48,527<br>(44,213 to 52,644) | 48,843<br>(44,533 to 53,049) | 47,262<br>(41,188 to 53,678) | 47,269<br>(41,144 to 53,706) | 47,276<br>(41,102 to 53,730) | 47,284<br>(41,028 to 53,768) | 47,291<br>(40,999 to 53,808) |
| Discounted CVD Cost (£) | 34,093<br>(30,689 to 37,556) | 34,187<br>(30,744 to 37,685) | 34,288<br>(30,801 to 37,840) | 34,398<br>(30,862 to 37,999) | 34,517<br>(30,927 to 38,155) | 32,084<br>(26,847 to 37,563) | 32,019<br>(26,724 to 37,525) | 31,951<br>(26,622 to 37,486) | 31,882<br>(26,488 to 37,426) | 31,811<br>(26,351 to 37,364) |
| Discounted CHD Cost (£) | 26,929<br>(24,194 to 29,707) | 26,966<br>(24,182 to 29,763) | 27,007<br>(24,171 to 29,834) | 27,052<br>(24,176 to 29,928) | 27,102<br>(24,170 to 30,025) | 23,272<br>(19,049 to 27,623) | 23,203<br>(18,933 to 27,575) | 23,131<br>(18,815 to 27,526) | 23,058<br>(18,694 to 27,462) | 22,983<br>(18,569 to 27,388) |
| Time to first event (years) | 9.11<br>(8.79 to 9.4) | 9.42<br>(9.11 to 9.7) | 9.74<br>(9.44 to 10.02) | 10.09<br>(9.79 to 10.35) | 10.45<br>(10.16 to 10.7) | 11.6<br>(11.47 to 11.72) | 11.82<br>(11.7 to 11.94) | 12.05<br>(11.93 to 12.16) | 12.28<br>(12.17 to 12.39) | 12.53<br>(12.42 to 12.63) |
| MI as primary endpoint (%) | 20.59<br>(19.89 to 21.36) | 19.06<br>(18.39 to 19.8) | 17.44<br>(16.79 to 18.15) | 15.71<br>(15.11 to 16.39) | 13.88<br>(13.32 to 14.5) | 10.15<br>(9.85 to 10.48) | 9.29<br>(9 to 9.59) | 8.39<br>(8.13 to 8.67) | 7.47<br>(7.23 to 7.72) | 6.51<br>(6.29 to 6.73) |
| Ischaemic stroke as primary endpoint (%) | 6.88<br>(6.4 to 7.46) | 6.4<br>(5.94 to 6.95) | 5.89<br>(5.45 to 6.41) | 5.33<br>(4.93 to 5.82) | 4.73<br>(4.36 to 5.18) | 7.7<br>(7.37 to 8.03) | 7.06<br>(6.76 to 7.37) | 6.4<br>(6.12 to 6.68) | 5.7<br>(5.45 to 5.96) | 4.99<br>(4.76 to 5.22) |
| Haemorragic stroke as primary endpoint (%) | 0.75<br>(0.66 to 0.84) | 0.77<br>(0.69 to 0.87) | 0.8<br>(0.71 to 0.9) | 0.83<br>(0.74 to 0.93) | 0.86<br>(0.76 to 0.97) | 0.73<br>(0.64 to 0.82) | 0.74<br>(0.65 to 0.84) | 0.75<br>(0.67 to 0.86) | 0.77<br>(0.68 to 0.87) | 0.78<br>(0.69 to 0.89) |
| CVD Mortality (%) | 25.3<br>(23.14 to 28.22) | 23.59<br>(21.53 to 26.39) | 21.75<br>(19.8 to 24.38) | 19.76<br>(17.98 to 22.21) | 17.61<br>(16 to 19.84) | 14.92<br>(13.15 to 16.44) | 13.72<br>(12.09 to 15.14) | 12.48<br>(10.98 to 13.78) | 11.17<br>(9.83 to 12.36) | 9.81<br>(8.63 to 10.87) |
| Non-CVD Mortality (%) | 74.7<br>(71.78 to 76.86) | 76.41<br>(73.61 to 78.47) | 78.25<br>(75.62 to 80.2) | 80.24<br>(77.79 to 82.02) | 82.39<br>(80.16 to 84) | 85.08<br>(83.56 to 86.84) | 86.28<br>(84.86 to 87.91) | 87.52<br>(86.22 to 89.02) | 88.83<br>(87.64 to 90.17) | 90.19<br>(89.13 to 91.37) |

* 1 year decrement post event
** constant decrement post event

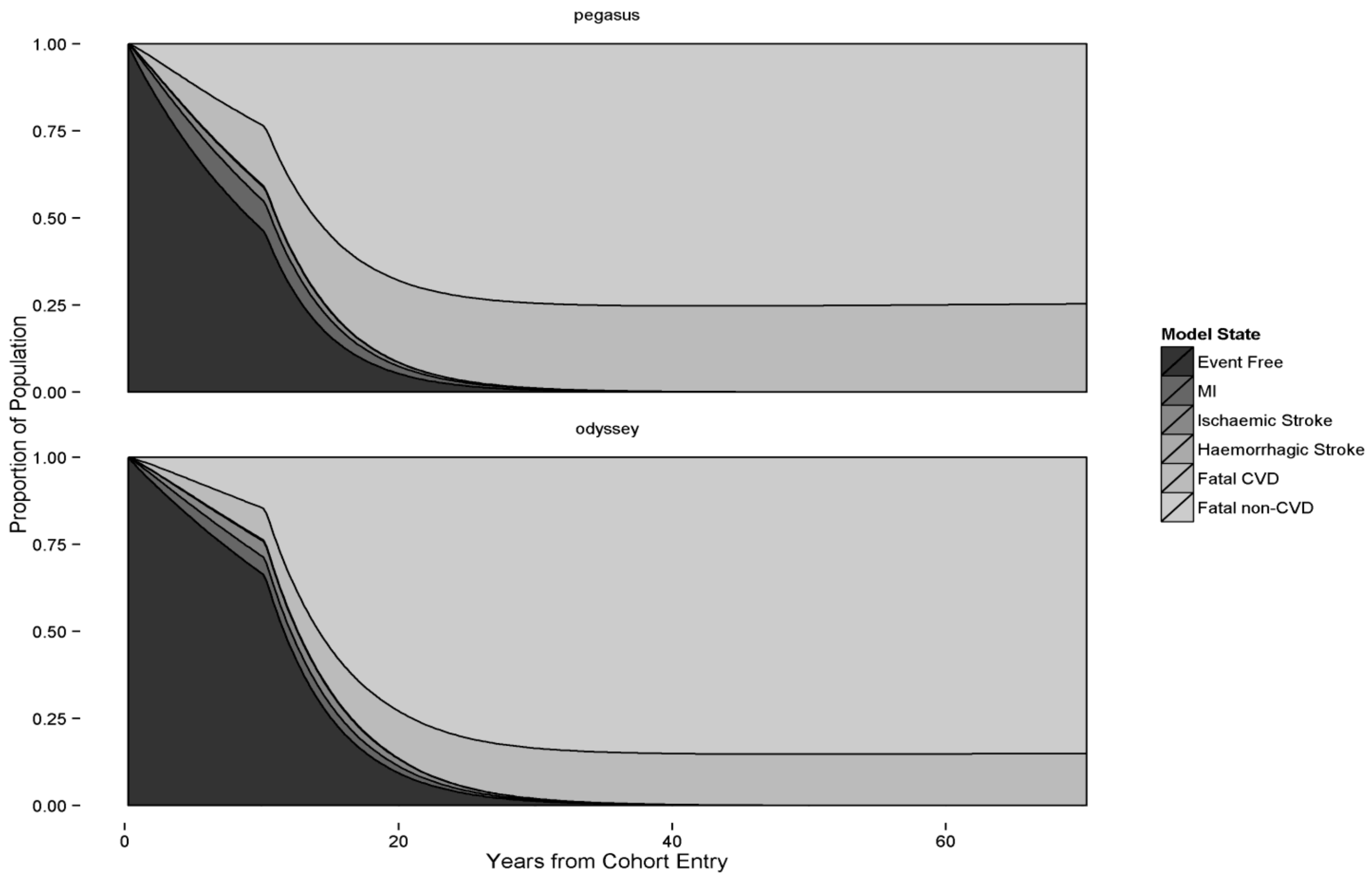
pegasus
odyssey
1.00
0.75
0.50
0.25
0.00
Proportion of Population
0
20
40
60
Years from Cohort Entry
Model State
Event Free
MI
Ischaemic Stroke
Haemorrhagic Stroke
Fatal CVD
Fatal non-CVD

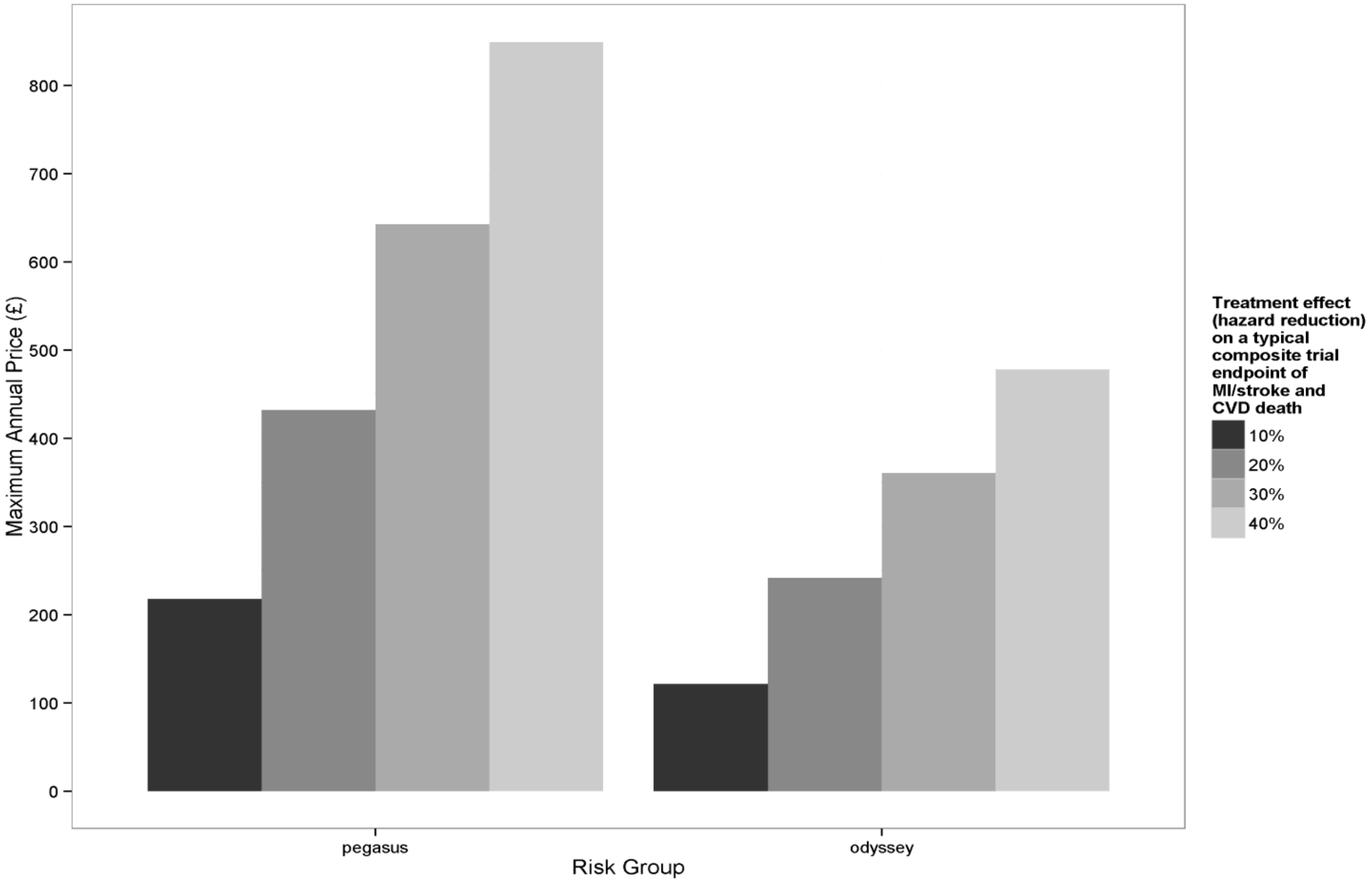
Maximum Annual Price (£)
800
700
600
500
400
300
200
100
0
pegasus
odyssey
Risk Group
Treatment effect (hazard reduction) on a typical composite trial endpoint of MI/stroke and CVD death
10%
20%
30%
40%
